# Dissecting cross‐lineage tumourigenesis under p53 inactivation through single‐cell multi‐omics and spatial transcriptomics

**DOI:** 10.1002/ctm2.70461

**Published:** 2025-08-31

**Authors:** Xinru Wang, Yuqing Mei, Xueyi Wang, Hanyu Wu, Renying Wang, Peijing Zhang, Guodong Zhang, Jiaqi Li, Mengmeng Jiang, Xing Fang, Lifeng Ma, Yuan Liao, Danmei Jia, Haofu Niu, E Weigao, Haide Chen, Lei Yang, Shuang Zhang, Tingyue Zhang, Yincong Zhou, Qi Zhang, He Huang, Hongwei Ouyang, Ming Chen, Tingbo Liang, Jinrong Peng, Jingjing Wang, Guoji Guo, Xiaoping Han

**Affiliations:** ^1^ Bone Marrow Transplantation Center of the First Affiliated Hospital and Center for Stem Cell and Regenerative Medicine Zhejiang University School of Medicine Hangzhou Zhejiang China; ^2^ Zhejiang Provincial Key Lab for Tissue Engineering and Regenerative Medicine, Dr. Li Dak Sum & Yip Yio Chin Center for Stem Cell and Regenerative Medicine Hangzhou Zhejiang China; ^3^ Liangzhu Laboratory Zhejiang University Medical Center Hangzhou Zhejiang China; ^4^ College of Life Sciences Zhejiang University Hangzhou Zhejiang China; ^5^ Department of Hepatobiliary and Pancreatic Surgery The First Affiliated Hospital, School of Medicine, Zhejiang University Hangzhou Zhejiang China; ^6^ Institute of Hematology Zhejiang University Hangzhou Zhejiang China; ^7^ MOE Key Laboratory for Molecular Animal Nutrition, College of Animal Sciences Zhejiang University Hangzhou Zhejiang China

**Keywords:** cross‐lineage tumourigenesis, knockout cell landscape analyses, p53 regulatory network, single‐cell multi‐omics

## Abstract

**Background:**

Tumour suppressor genes, exemplified by *TP53* (encoding the human p53), function as critical guardians against tumourigenesis. Germline *TP53*‐inactivating mutations underlie Li‐Fraumeni syndrome, a hereditary cancer predisposition disorder characterised by early‐onset pan‐tissue malignancies. However, the context‐dependent tumour‐suppressive mechanisms of p53 remain incompletely elucidated. This study aims to investigate the disruption of cellular homeostasis and tumourigenic mechanisms following p53 inactivation across distinct cell lineages.

**Methods:**

*Trp53* (encoding mouse p53) knockout mouse model was employed to study molecular alterations under p53‐deficient conditions. Multi‐omics analyses – including single‐cell transcriptomics, single‐cell ATAC‐seq, spatial transcriptomics, whole genome sequencing, and CUT&Tag – were integrated to construct a *Trp53* functional cell landscape. Deep learning‐based gene network models were employed to reconstruct p53 regulatory networks and simulate in silico perturbations caused by p53 loss.

**Results:**

Our analyses revealed transitional dynamics in immune, stromal, and epithelial cells from normal physiology to p53‐deficient states and subsequent tumourigenesis. These transitions implicated critical pathways such as cell cycle regulation, stress response, metabolic reprogramming, and immune modulation, displaying both lineage‐conserved and lineage‐specific features. Tumour‐prone cell populations exhibiting elevated differentiation plasticity were identified across lineages within tumourigenic trajectories. Spatial transcriptomic profiling confirmed the emergence of thymic tumour‐initiating T‐cell clusters characterised by deterministic chromatin architectural disruptions under p53‐loss pressure. Notably, we uncovered a recurrent upregulation signature of ribosomal protein genes as an early pivotal molecular event preceding malignant transformation in p53‐deficient oncogenesis. Finally, we decoded the p53 downstream regulatory network and computationally evaluated the perturbation effects of genetic inactivation at single‐cell resolution.

**Conclusions:**

Our results elucidate the multiscale consequences of p53 inactivation while providing valuable resources for understanding tumour predisposition associated with p53‐inactivating mutations and developing clinical interception strategies.

**Key points:**

- Construction of a *Trp53* functional cell landscape utilising single‐cell multi‐omics and spatial omics technologies.
- Reconstruction of p53 downstream regulatory relationships with lineage heterogeneity via machine learning‐based gene network modelling.
- Dissection of shared and lineage‐specific features during cross‐lineage tumourigenesis under p53 deficiency.

## Graphical abstract

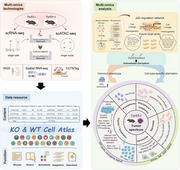

## 1 BACKGROUND

As a critical tumour suppressor gene, the evolutionarily conserved p53 has been intensively studied since its discovery in 1979.^1^ P53 exhibits effective functions in promoting DNA repair, cell‐cycle arrest, senescence, and apoptosis.^2^ *TP53* gene, which encodes human p53 protein, is mutated in over 50% of all human cancers,^3^ with p53 signalling pathway disruptions observed in over 80% of tumour samples, profoundly impacting malignancy, metastasis, therapeutic resistance, and clinical prognosis.^4^ Notably, inactivating germline *TP53* mutations define the diagnostic hallmark of Li‐Fraumeni syndrome (LFS), a prototypical hereditary cancer predisposition disorder.^5^ These observations underscore p53's pan‐tumour suppressive roles and indispensable function in maintaining cellular integrity. Furthermore, the causal link between p53 activity attenuation and tumourigenesis highlights its central role in homeostatic regulation in response to a range of common but moderately intense conditions.^6^

Functioning as a master sequence‐specific transcription factor (TF) with potential binding sites distributed throughout mammalian genomes,^7^ p53 governs complex gene regulatory networks.^8^, ^9^ Its transcriptional activity is essential for tumour suppression.^10^ While some p53‐responsive elements exhibit species‐specific evolutionary divergence, core network architectures remain highly conserved.^2^ Moreover, to execute chromatin‐modifying functions, p53 recruits diverse transcriptional cofactors including chromatin remodellers and modifying enzymes that serve as context‐dependent co‐activators or co‐repressors.^11^ Numerous studies have focused on identifying p53 downstream targets,^12^ analysing the upstream signalling pathways,^13^ and elucidating the co‐activators.^14^ These efforts have revealed p53's multifaceted roles in canonical tumour suppression, homeostasis maintenance, and emerging biological processes spanning epigenetics, senescence, metabolism, immunity, development, regeneration, ferroptosis, and neurodegeneration.^14^, ^15^, ^16^, ^17^, ^18^, ^19^, ^20^, ^21^, ^22^ Recent discoveries are reshaping the conventional view of p53 as primarily pro‐apoptotic, gradually deciphering its precise regulatory roles in maintaining cellular homeostasis across diverse biological contexts.

Mounting evidence demonstrates that p53's transcriptional output displays remarkable cell type specificity and contextual dependency.^11^ Rather than following a universal response program, p53 dynamically adjusts its binding sites and downstream effects according to lineage‐specific chromatin landscapes.^23^ However, current understanding of p53's lineage‐common versus lineage‐specific regulatory mechanisms remains fragmented. While foundational insights into p53 biology have emerged from prior investigations, existing paradigms have been constrained by analytical fragmentation: bulk tissue analyses that masked cellular heterogeneity, immortalised cell lines lacking physiological context, or isolated pathway/tissue examinations unable to capture systemic crosstalk.^24^, ^25^, ^26^ Given recent advances in single‐cell technology and the cell‐atlas strategy,^27^, ^28^, ^29^, ^30^, ^31^, ^32^, ^33^, ^34^, ^35^, ^36^ it would be ideal to perform an integrative analysis of the cell landscape from a whole organism upon perspective *Trp53* perturbation. Genetically engineered mouse models provide unique platforms for dissecting p53 functional networks.^37^ Clinically, *TP53* loss of function (and associated dominant‐negative effects) is the main mechanism that will impair its tumour suppressive function.^38^, ^39^ Consistent with human pathology, *Trp53* knockout (KO) mice develop spontaneous multi‐lineage tumours with high penetrance, offering an ideal system to investigate p53's multifaceted regulatory roles under basic expression conditions. Longitudinal tracking of cellular features across tumourigenic stages in p53‐deficient models will elucidate cell‐intrinsic drivers of homeostatic dysregulation and trans‐lineage oncogenesis, while providing critical insights for developing LFS clinical interception strategies.

Here, we used multi‐omics approaches to decode the altered molecular program during tumourigenesis after *Trp53* knockout. A cross‐lineage comparative framework was implemented: cells were systematically classified into discrete lineages like epithelial, immune and stromal to enable direct comparison of conserved versus lineage‐specific responses. Our study unveiled the transitional changes in the molecular signatures of immune, stromal, and epithelial cells from normal to p53‐deficient tumour states. We pinpointed tumour‐prone cells exhibiting high differentiation potential phenotypes across different lineages and tumour‐initiating cells with deterministic increased mutational burden. Moreover, by integrating KO and wild‐type (WT) datasets for comparative analysis, we found that p53 deficiency induces molecular alterations across virtually all cell types, including those lineages where capturing overt tumour phenotypes remains challenging. We identified the conserved upregulated ribosomal protein gene (RPG) cluster across diverse cell types following p53 loss preceding tumourigenesis. We have also developed a deep learning‐based strategy to decipher the intricate regulatory architecture of p53. Overall, we provided the very first pilot example of a KO atlas study to characterise the multi‐lineage tumourigenesis with p53 loss of function at the organism‐scale and single‐cell resolution.

## 2 METHODS

### 2.1 Experimental animals

The C57BL/6‐*Trp53* ^−/−^ mice were ordered from Biocytogen Pharmaceuticals (Beijing) Co., Ltd. The Wild‐type C57BL/6 mice were ordered from GemPharmatech Co., Ltd. To generate sufficient F1 C57BL/6‐*Trp53* ^+/−^ mice, the male C57BL/6‐*Trp53* ^−/−^ mice were crossed with the female wild‐type C57BL/6 mice (The female C57BL/6‐*Trp53* ^−/−^ mice have serious reproductive disorders). To obtain more homozygous *Trp53* knockout mice and wild‐type mice from the same generation, the male C57BL/6‐*Trp53* ^−/−^ and C57BL/6‐*Trp53* ^+/−^ mice were crossed with heterozygous female mice and the genotypes of the F2 mice were identified by PCR. Genders were not considered. All the mice were housed and bred at Zhejiang University Laboratory Animal Center. Mouse experiments in this study were approved by the Animal Ethics Committee of Zhejiang University; experiments conformed to the regulatory standards at Zhejiang University Laboratory Animal Center. The specific number of mice used for each omics analysis is detailed in Table S1.

### 2.2 Tissue collection

For multi‐omics sequencing, tissues were harvested from C57BL/6‐Trp53–/– mice and wild‐type C57BL/6 mice. The number of tissue samples utilised per omics modality is specified in Table S1.

### 2.3 Fabrication of microwell device

The diameter of the microwells used in updated Microwell‐seq was 28 µm and the depth is 35 or 30 µm, depending on different cell sizes, with the diameter 32µm and the depth 45 µm used in Microwell ATAC‐seq to accommodate more nuclei. The new silicon plate was ordered from Suzhou Cchip Scientific Instrument Co., Ltd. A reusable polydimethylsiloxane (PDMS) plate, featuring micropillars identical in number to those of a silicon microwell template, was fabricated using the silicon plate as a mould. For experimental use, a disposable agarose microwell plate was prepared immediately beforehand by casting a 5% agarose solution over the surface of this PDMS plate. Both the original silicon template and the resultant PDMS replica are designed for repeated use.

### 2.4 Synthesis of barcoded beads

The prior protocol was enhanced for efficient three‐segment molecular chain synthesis. Carboxyl‐coated magnetic beads (20 µm for updated Microwell‐seq, 28 µm for Microwell ATAC‐seq; Suzhou Knowledge & Benefit Sphere Tech) underwent three split‐pool cycles for surface oligonucleotide barcoding. All the sequences used for the beads in updated Microwell‐seq beads and Microwell ATAC‐seq were listed in Tables S2 and S3.

For the first batch of bead synthesis, 300–350 µL carboxyl beads (50 mg/mL) were washed with 0.1 M MES, suspended in 635 µL MES, mixed with 3.08 mg EDC, and aliquoted (6.2 µL/well) into a 96‐well plate. Each well received 2.5 µL amino‐modified oligonucleotide (50 µM in MES). After vortexing and 20 min RT incubation, 0.5 µL EDC mix (6 mg in 100 µL MES) was added twice (20 min RT incubation after each addition, 80 min after second). Beads were pooled in 1 mL PBS‐Tween (0.02%), centrifuged, supernatant removed, and washed twice with 1 mL TE (pH 8.0).

In the second split‐pool, beads washed with 10 mM Tris‐HCl (pH 8.0) were aliquoted into a new plate. Each well received mix (1×NEBuffer, 1 mM dNTPs, 50 µM oligonucleotide; 5 µL dNTPs, 1 µL oligo/well). Oligos contained Linker 1 RC, unique barcode, and Linker 2. After 10 s at 95°C (pre‐shaken), plates were mixed (10 rpm, RT, 10‐20 min). Polymerase mastermix (1× NEBuffer 2, 200 U/mL Klenow; 2.5 µL/well) was added, followed by polymerisation (37°C, 10 rpm, 1 h). Beads were quenched on ice with 0.15 M EDTA (1.5 µL/well), pooled, washed twice with 0.1 M NaOH (30 s RT), then twice with TE‐TW and once with Tris‐HCl.

The third split‐pool procedure is identical to the second one, but oligos encoded Linker 2 RC, unique barcode, UMI, and poly‐T. Post‐synthesis, beads were washed twice with TE‐SDS (inactivate Klenow), once with TE‐TW, once with Tris‐HCl. To remove incomplete chains, beads were treated with exonuclease I mix (1× buffer, 1 U/µL; 200 µL, 37°C, 10 rpm, 15 min), stopped by TE‐SDS/TE‐TW washes. Complementary chains were removed via NaOH denaturation (as above). Beads were washed twice with TE‐TW and stored in TE‐TW (1 mL, 4°C, ≤4 weeks).

### 2.5 Updated Microwell‐seq

#### 2.5.1 Sample preparation

Organs and tissues were harvested from 3‐8 month‐old mice (C57BL/6‐Trp53–/– or wild‐type C57BL/6; see Tables S4 and S5). Following intraperitoneal anaesthesia with 1% sodium pentobarbital, animals were weighed, shaved, and perfused transcardially with 20‐30 mL phosphate‐buffered saline. Dissection proceeded in this sequence: small intestine, large intestine, spleen, pancreas, lymph nodes (inguinal, abdominal aortic, axillary, cervical), stomach, heart, lung, thymus, brain, kidney, ovary, uterus, bladder, mammary glands (fat pads 4), liver, gallbladder with bile duct, and limb bone marrow. All samples except bone marrow were immediately placed in ice‐cold Dulbecco's phosphate‐buffered saline, minced into ∼1 mm fragments, transferred to 15 mL tubes, rinsed twice with cold DPBS, and suspended in 5 mL tissue‐specific dissociation enzymes. Enzymatic treatment durations varied per tissue (Table S5). Gentle pipetting continued until tissue fragments disappeared. For erythrocyte‐rich pellets, red blood cell lysis buffer (Biolegend) was applied (5 min), quenched with 14 mL DPBS containing 2 mM EDTA. Cells were centrifuged (300 × *g*, 5 min, 4°C), suspended in 5 mL DPBS with 2 mM EDTA, filtered through 40 µm strainers (Biologix), washed twice, and resuspended at 2 × 10⁵ cells/mL in cold DPBS/2 mM EDTA.

Bone marrow was extracted from femurs and tibiae after muscle removal using Delicate Task Wipers. Bone ends were trimmed to expose marrow cavities, flushed with DPBS with 2 mM EDTA using a 1 mL syringe (26G needle), and collected in 15 mL tubes. After centrifugation (300 × *g*, 5 min, 4°C), pellets were resuspended in DPBS, incubated at 37°C for 15 min, vortexed in three 5‐s bursts, and settled on ice for 1 min. Supernatant was retained, erythrocytes lysed, and cells filtered through 40 µm strainers. Final suspension was adjusted to 2 × 10⁵ cells/mL in DPBS with 2 mM EDTA.

#### 2.5.2 Single‐cell collection and lysis

Cell concentration was optimised (∼200 000/mL; ∼10% microwell occupancy) using a haemocytometer; bead concentration was ∼1 000 000/mL (single bead/well). Single cells from each mouse tissue were isolated into individual microwells. An evenly distributed cell suspension (∼500 µL) was loaded onto the array. Doublets were minimised microscopically and via pipetting. After cell settling, excess supernatant was aspirated. Bead suspension (∼500 µL) was added, the plate magnetised, beads shaken into wells, and excess washed away. Cold lysis buffer [0.1 M Tris‐HCl pH 7.5, 0.5 M LiCl, 1% SDS, 10 mM EDTA, 5 mM DTT] was applied for 15 min. Beads were collected (∼90 000), washed sequentially with 1 mL 6× SSC, 500 µL 6× SSC, and 200 µL 50 mM Tris‐HCl pH 8.0.

#### 2.5.3 Reverse transcription

In this procedure, 20 µL RT mix [200 U SuperScript II, 1× Superscript II buffer (Takara), 40 U Murine RNase inhibitor (Vazyme), 1 M betaine, 6 mM MgCl_2_, 2.5 mM DTT, 1 mM dNTP, 1 µM TSO LNA primer] was added. Incubation: 42°C, 90 min, 10 rpm. Reverse transcriptase was inactivated by TE‐SDS wash. The sequence information for the primers was included in Table S2.

#### 2.5.4 Exonuclease I treatment

Beads were washed (TE‐TW, Tris‐HCl pH 8.0), resuspended in 100 µL exonuclease I mix [1× buffer, 50 U enzyme (NEB)], and incubated (37°C, 60 min, 10 rpm) to remove unused oligos.

#### 2.5.5 Second strand synthesis

mRNA‐cDNA hybrids were denatured (2× 0.1 M NaOH, 30 s RT). Washes: 2× TE‐TW, Tris‐HCl pH 8.0. Beads were resuspended in 20 µL dn‐TSO oligo mix [5 mM oligo (Table S2), 10 mM Tris‐HCl pH 8.0, 3 mM MgCl_2_], heat‐denatured (95°C, 30 s), and bound (RT, 10 min, 10 rpm). After aspiration/Tris‐HCl wash, beads were combined with 50 µL mastermix [1× RT buffer (Thermo), 12% PEG8000, 1 mM dNTPs, 6.25 U Klenow exo‐ (Vazyme)] for second‐strand synthesis (37°C, 1 h, 10 rpm).

#### 2.5.6 cDNA amplification

Beads were transferred to a PCR tube with 25 µL PCR mix [1× Kapa HiFi HotStart Readymix, 0.4 µM TSO‐PCR primer (Table S2)]. Program: 95°C 3 min; 3 cycles (98°C 20 s, 65°C 45 s, 72°C 6 min); 72°C 5 min; 4°C hold. Pooled products received 50 µL fresh PCR mix [same reagents] and amplified: 95°C 3 min; 12 cycles (98°C 20 s, 67°C 20 s, 72°C 3 min); 72°C 10 min; 4°C hold. Finally, the cDNA library underwent purification with 0.9X VAHTS DNA Clean beads (Vazyme).

#### 2.5.7 Transposase fragmentation, selective PCR and sequencing

Purified cDNA was fragmented using customised transposase (TruePrep Homo‐N7 Kit, Vazyme) per manufacturer. Selective PCR used platform‐specific primers (Illumina: P5 + VAHTS Adapters set 3–6; MGI: P5 + Indexed P7 primers – sequences Table S2) to amplify 3' transcript ends. Program: 72°C 3 min; 98°C 1 min; 5 cycles (98°C 15 s, 60°C 30 s, 72°C 3 min); 72°C 5 min; 4°C hold. Purification: 0.9X VAHTS beads. A second PCR (25 µL mix: 1× Kapa HiFi HotStart, 0.2 µM platform‐specific P5 and P7 primers) used: 95°C 3 min; 5 cycles (98°C 20 s, 60°C 15 s, 72°C 15 s); 72°C 3 min; 4°C hold. Size selection (0.55‐0.15X VAHTS beads) yielded fragments (400–700 bp, Agilent 2100). Sequencing used Illumina HiSeq or MGI DNBSEQ‐T7. For MGI: VAHTS Circularization Kit and custom Read1 X‐linker primers (Table S2) were applied.

### 2.6 Microwell ATAC‐seq

#### 2.6.1 Tn5 tagging plate preparation

Two microlitres of 100 µM primer A (in annealing buffer) and 2 µL of 100 µM indexed primer B (dissolved in annealing buffer; harbouring unique well‐specific barcodes) were dispensed into each well of a nuclease‐free 96‐well plate. Primer sequences are documented in Table S3. Annealing proceeded under this thermal profile: 95 degrees Celsius for 3 min; cooling to 75°C (10 min), 60°C (10 min), 50°C (10 min), and 40°C (10 min) at −0.1°C/s; 25°C for 30 min; final hold at 4°C. Separately, 100 µL TruePrep Tagment Enzyme (Vazyme, S111‐01/02) was combined with 360 µL coupling buffer; 4.3 µL of this mixture was aliquoted per well into a secondary plate. Post‐annealing, 1 µL of annealed adapter mix was transferred from each original well to corresponding wells of the secondary plate. Incubation occurred at 30°C for 60 min in a thermal cycler. Subsequently, 20 µL storage buffer was introduced per well, and the final mixture was subdivided (2 µL/well) into fresh plates. Plates were sealed and stored at −20°C (≤1 year).

#### 2.6.2 Sample preparation

Organs were harvested from C57BL/6‐Trp53–/– or wild‐type C57BL/6 mice (3–8 months old; Table S6). Following intraperitoneal anaesthesia with 1% sodium pentobarbital, mice underwent weighing, shaving, and transcardial perfusion with 20–30 mL phosphate‐buffered saline. Dissection sequence: small intestine, large intestine, spleen, pancreas, lymph nodes (inguinal, abdominal aortic, axillary, cervical), stomach, heart, lung, thymus, brain, kidney, ovary, uterus, bladder, mammary glands (fat pads 4), liver, gallbladder with bile duct, salivary gland, and bone marrow (protocol as previously described). Non‐marrow tissues were immediately immersed in ice‐cold Dulbecco's phosphate‐buffered saline, washed, and pulverised under liquid nitrogen using mortar and pestle. Pulverised material was distributed into pre‐cooled, labelled nuclease‐free tubes (storable at –80°C). Tissue powder was suspended in 1 mL Nuclear Permeabilisation Buffer for 3 min to liberate nuclei. Bone marrow suspensions were centrifuged (350 × *g*, 5 min); pellets were directly resuspended in 1 mL Nuclear Permeabilisation Buffer (5 min). Suspensions were transferred to 15 mL tubes prefilled with 5 mL s wash buffer, filtered through 100 µm strainers, and centrifuged (600 × *g*, 5 min, 4°C). Nuclei were resuspended in 3 mL phosphate‐buffered saline and crosslinked via dropwise addition of 1 mL 4% formaldehyde (gentle agitation; final 1% concentration). After 10 min room temperature incubation (tubes inverted gently every 90 s), crosslinking was terminated with 200 µL 2.5 M glycine per tube. Solutions were mixed by inversion, incubated 5 min (room temperature), then 15 min (ice). Nuclei were filtered (40 µm strainers), pelleted (600 × *g*, 5 min), and resuspended in 1 mL PBS‐B buffer. Fixed nuclei were quantified by haemocytometer and adjusted to ∼1 000 000 nuclei per 1.5 mL tube in 1 mL freezing buffer containing 1× protease inhibitors and 5 mM DTT. Aliquots were flash‐frozen in liquid nitrogen and stored at –80°C (≤1 week).

#### 2.6.3 Tn5 tagging of nuclei

Fixed nuclei stored at –80°C were thawed on dry ice, followed by brief warming (37°C water bath, 0.5–1 min). Nuclei were pelleted by centrifugation (600 × *g*, 5 min, 4°C), washed twice with PBS‐B buffer, and resuspended in minimal PBS‐B volume. Concentration was adjusted to 200 000 nuclei/mL using haemocytometer quantification under fluorescence microscopy with 1% Ultra GelRed DNA dye (Vazyme). Pre‐chilled Tn5 plates received 6 µL tagmentation buffer mix (2.5 µL DEPC‐treated water, 2.5 µL 4× TD buffer, 1 µL 0.1% Digitonin) per well, centrifuged (3000 × *g*, 5 min, 4°C) for mixing. Two microliters of nuclei suspension were added per well with source documentation. To prevent cross‐contamination, nuclei from distinct tissues were processed in separate wells. Tagmentation proceeded at 55°C for 30 min (rotary mixer, 10 rotations per minute). Reactions were terminated with 10 µL 40 mM EDTA per well (37°C, 10 min, 10 rotations per minute). Osmotic pressure was adjusted with 20 µL wash buffer per well. Tagmented nuclei were pooled per tissue into 15 mL tubes, washed with 3 mL cold RSB buffer, and processed separately thereafter.

#### 2.6.4 SDS stripping and exonuclease I treatment

Nuclei were suspended in 1.5 mL RSB‐SDS for 10 min at ambient temperature to expose terminal deoxyribonucleotidyl transferase binding sites. Reactions were quenched with 8.5 mL RSBT‐BD buffer per tube, followed by centrifugation (600 × *g*, 5 min, 4°C). Pellets were washed twice with 1 mL RSBT‐BD buffer and resuspended in 100 µL exonuclease I mix [1× buffer, 50 units enzyme (NEB)]. Suspensions were distributed equally (12.5 µL/well) into nuclease‐free 8‐strip tubes and incubated on ice for 5 min. Exonuclease treatment: 37°C (10 min), 45°C (5 min), 50°C (5 min), 55°C (5 min + hold). Tubes were immediately chilled on ice (3 min), supplemented with 12.5 µL fresh exonuclease I mix per well, and subjected to identical thermal cycling. Nuclei were recombined into 15 mL tubes and washed sequentially with cold WB‐T and RSBT‐B buffers.

#### 2.6.5 Adding A‐tail to DNA fragments

Nuclei were quantified (haemocytometer, 1% Ultra GelRed) and standardised to ∼1 000 000 nuclei per 1.5 mL tube. After centrifugation (600 × *g*, 5 min), supernatants were meticulously aspirated. Pellets were resuspended in 50 µL TdT mix [1× TdT buffer, 5 mM CoCl_2_, 100 µM dATP, 400 units terminal deoxyribonucleotidyl transferase (Roche)] and incubated (37°C, 20 min, 10 rotations per minute). Reactions were halted with 50 µL 40 mM EDTA.

#### 2.6.6 Beads preparation

Bead aliquots (∼500 000 beads per microwell) were treated twice with 200 µL 0.1 molar sodium hydroxide (30 s, ambient temperature). Subsequent washes included: two washes with 1 mL TE‐TW, two washes with 1 mL PBS, and final suspension in 500 µL PBS.

#### 2.6.7 Nucleus collection and lysis

Nuclei (∼400 000/mL; quantified via haemocytometer with 1% Ultra GelRed) in suspension (∼500 µL) were pipetted onto microwells. Centrifugation (300× *g*, 15 s, 4°C) enhanced loading density. After supernatant removal, beads (∼500 µL) were loaded, magnetically captured, shaken into wells, and washed. Lysis proceeded for 30 min at 37°C with 150 µL buffer [1% SDS, Vazyme Blue buffer (1×), Vazyme PCR Enhancer (1×), 5% PEG8000, 10 mM EDTA, Sangon protease K (1 mg/mL)]. Beads were harvested in 2× SSC, washed sequentially with 2× SSC (500 µL) and 10 mM Tris‐HCl pH 8.0 (200 µL).

#### 2.6.8 Exonuclease I treatment and DNA strands extension

Following magnetic clearance, beads were treated with 50 µL exonuclease I mix [1× buffer, 50 U enzyme (NEB)] for 15 min (37°C, 10 rpm). Washes: TE‐SDS, TE‐TW, 10 mM Tris‐HCl pH 8.0. DNA extension used 50 µL mix [NEBuffer (1×), Vazyme PCR Enhancer (1×), 5% PEG8000, 1 mM dNTP, Klenow (6.25 U, NEB)] incubated 30 min (37°C, 10 rpm). Terminated by TE‐SDS wash, followed by TE‐TW (2×) and Tris‐HCl washes.

#### 2.6.9 cDNA amplification, library construction and sequencing

Amplification initiated with 50 µL PCR mix [Kapa HiFi HotStart (1×), ATAC‐F/R primers (0.4 µM each; Table S3)], distributed (12.5 µL/well). Cycling: 95°C 3 min; 2 cycles (98°C 30 s, 65°C 60 s, 72°C 60 s); 4 cycles (98°C 30 s, 65°C 30 s, 72°C 60 s); 72°C 3 min; 4°C. Post‐magnetic separation, supernatant purified (0.9× VAHTS beads), eluted in 21 µL DEPC H_2_O. Second PCR: 29 µL/well mix [Kapa HiFi (1×), P5‐ATAC/indexed P7‐ATAC primers (0.4 µM; Table S3)]. Cycling: 95°C 5 min; 2 cycles (98°C 30 s, 65°C 60 s, 72°C 60 s); 8 cycles (98°C 30 s, 65°C 30 s, 72°C 60 s); 72°C 3 min; 4°C. Size‐selected (0.4–0.5× VAHTS beads). MGI DNBSEQ‐T7 sequencing employed VAHTS Circularization Kit and custom R1 X‐linker 1 and 2 primers (Table S3).

#### 2.6.10 WGS (whole genome sequencing)

Genomic DNA was isolated from tissues of C57BL/6‐Trp53–/– or wild‐type C57BL/6 mice (including bone marrow, spleen, lymph nodes, thymus, small intestine, mammary gland, liver, intraperitoneal masses, subcutaneous masses, mesenteric masses, and large intestine lumps) using the FastPure Blood/Cell/Tissue/Bacteria DNA Isolation Mini Kit (Vazyme) per manufacturer protocols. Comprehensive sample details appear in Table S7. DNA concentrations were quantified via Qubit fluorometer (ThermoFisher Scientific) to determine input amounts. Sequencing libraries were constructed with the TruePrep DNA Library Prep Kit for MGI (Vazyme). Whole genome sequencing was conducted on the MGI DNBSEQ‐T7 platform, generating 150 base‐pair paired‐end reads with median 25‐fold coverage.

### 2.7 CUT&Tag(Cleavage Under Targets & Tagmentation)

The 18 organs and tissues including small intestine, large intestine, spleen, pancreas, lymph nodes (inguinal, abdominal aortic, axillary, cervical), stomach, heart, lung, thymus, brain, kidney, ovary, uterus, bladder, mammary glands (fat pads 4), liver, gallbladder with bile duct, and limb bone marrow – were harvested from 3‐month‐old wild‐type C57BL/6 mice (triplicate biological replicates; Table S8). The Hyperactive Universal CUT&Tag Assay Kit for Illumina with anti‐p53 antibody (Abcam #ab246550) was employed following standard protocols. Amplification utilised Universal P5 primers for MGI and indexed P7 primers (sequences: Table S1). Libraries were sequenced on MGI DNBSEQ‐T7 (150 base‐pair paired‐end reads) per manufacturer guidelines.

### 2.8 Spatial transcriptomics

Gene Expression (BMKMANU ST03002) and Tissue Optimization (BMKMANU ST03003) kits were implemented per manufacturer specifications. Gene expression slides feature 6.8 × 6.8 mm^2^ capture areas containing 2 200 000 barcoded spots (2.5 µm diameter; 4.8 µm centre–centre spacing), each capturing 3–6 cells. Tissues embedded in Optimal Cutting Temperature compound (OCT, Sakura Tissue‐TEK) on dry ice were stored at –80°C. Preliminary Tissue Optimization established permeabilisation parameters via fluorescent footprint imaging (Metafer Slide Scanning Platform, Pannoramic MIDI). Cryosectioning of OCT blocks (pre‐cooled cryostat; 10 µm thickness) transferred sections to oligo‐barcoded areas of BMKMANU S1000 Gene Expression Slides. Tissue‐mounted slides underwent fixation, haematoxylin and eosin staining, and imaging (Pannoramic MIDI microscope; 40× magnification), with tile scanning for image merging. Library construction followed BMKMANU ST03002‐34 protocols. TruSeq Illumina libraries were sequenced on NovaSeq (Illumina) by Integragen (Evry) at minimum 150 000 read‐pairs per spot using: Read 1 (28 cycles), i7 index (10 cycles), i5 index (10 cycles), Read 2 (50 cycles), generating 300–400 million reads.

Thymus permeabilisation optimisation employed BMKMANU S1000 Tissue Optimization reagents (BMKMANU, China). Subsequent gene expression analysis used 10 µm sections with sequential staining: 1 min isopropanol, 3 min haematoxylin, 30 s eosin (1:20 dilution). Permeabilisation durations: 25 min (wild‐type), 27 min (knockout). Comprehensive parameters are catalogued in Table S9.

### 2.9 Haematoxylin and eosin and immunohistochemistry staining

Wild‐type and p53 knockout tissue specimens underwent fixation in 4% paraformaldehyde (Sangon Biotech, E672002‐0500) for 48 h prior to paraffin embedding. Five‐micrometre sections were prepared from paraffin blocks for haematoxylin and eosin staining or immunohistochemical analysis.

For immunohistochemistry, deparaffinisation was achieved through graded xylenes (45 min) followed by graded ethanol (20 min). Antigen retrieval employed citrate buffer pH 6.0 (G1202, Servicebio) for 3 min. Slides were subsequently submerged in phosphate‐buffered saline (pH 7.4) with gentle rotation for 15 min. Blocking procedures included 0.3% hydrogen peroxide (25 min) and 3% bovine serum albumin (30 min). Primary antibodies (CD3 GB11014‐100, CD19 GB11061‐1‐100, NK‐p44 GB11615‐100, Col1a1 GB11022‐100, Fn1 GB114491‐100; Servicebio) were applied overnight at 4°C. Following PBS washes, slides were incubated with horseradish peroxidase‐conjugated Goat Anti‐Rabbit IgG (H+L) (GB23303, Servicebio) at ambient temperature for 50 min. Visualisation utilised Dako REAL™ DAB+ Chromogen with Dako REAL™ Substrate Buffer (K5007, DAKO) under light microscopy (XSP‐C204, CIC). Counterstaining with haematoxylin solution proceeded for 3 min. Final slide imaging was performed using a Pannoramic MIDI scanner.

### 2.10 EdU‐based cell proliferation assay in vivo and PI staining

The proliferation of cells was detected using BeyoClick™ EdU Cell Proliferation Kit with Alexa Fluor 488 (Beyotime, C0071S) and PI staining kit (Beyotime, C1052) according to the manufacturer's instructions. Dissolve the EdU powder (Beyotime, ST067) in PBS to prepare a stock solution of 3 mg/mL, and store it at –20°C. Administer the EdU solution to both WT and p53‐KO mice through intraperitoneal injection, with a dosage of 30 mg/kg, administered at 24‐h intervals for a total of three injections. On the fourth day, harvest the thymus, lymph nodes, and spleen tissues from the mice, and isolate all immune cells through tissue disaggregation. After washing with PBS, cells derived from various tissues were individually fixed in 4% paraformaldehyde solution for 15 min and subsequently permeabilised with 0.3% Triton X‐100 (Sigma, 93443) for an additional 15 min. For the detection of EdU, cells were incubated with the click‐reaction mixture for 30 min at room temperature in darkness (The additional negative control groups lacking the CuSO_4_ component was included). Following this, the reaction mixture was centrifuged, and the cells were washed twice with PBS and resuspended. PI staining was performed by resuspending fixed cell pellets in the PI staining solution and incubating them at 37°C for 30 min. Next, the samples were filtered through the 40µm filters prior to flow cytometric analysis. Three independent experiments were conducted, and gating and data fitting were performed using Kaluza software to determine the proliferation ratio of cells entering the S phase and the distribution ratio of the instantaneous cell cycle for each sample.

### 2.11 *TP53* sequencing of human cancer tissues

All patients gave their written informed consent for scientific evaluations. The study was approved by the Ethics Committee of The First Affiliated Hospital and The Second Affiliated Hospital, Zhejiang University School of Medicine. Surgical specimens of malignant and adjacent tissues were acquired from patients, preserved at –80°C, and documented in Table S10.

Genomic DNA extraction employed Vazyme's FastPure Blood/Cell/Tissue/Bacteria DNA Isolation Mini Kit. Amplification of TP53 coding/regulatory regions utilised 100–300 ng cDNA in 50 µL polymerase chain reaction mixture containing: 1× EVO Buffer (with 10 mM magnesium chloride), 1× PCR Enhancer, 1 mM deoxynucleotide triphosphates, 0.4 µM Primer_F, 0.4 µM Primer_R, and 1 unit Phanta EVO HS Super‐Fidelity DNA Polymerase (Vazyme). Thermal cycling parameters: 95°C (3 min); 25 cycles of 95°C (20 s), 63°C (20 s), 72°C (5 min); 72°C (7 min); 10°C hold. Primer sequences are catalogued in Table S10. Libraries were prepared using Vazyme's TruePrep DNA Library Prep Kit for MGI. Sequencing on MGI DNBSEQ‐T7 generated 150 base‐pair paired‐end reads with median 50‐fold coverage.

### 2.12 scRNA‐seq data analysis

#### 2.12.1 Preprocessing of scRNA‐seq data

The raw Microwell‐seq data were processed following the Drop‐seq^40^ core computational protocol (http://mccarrolllab.org/wp‐content/uploads/2016/03/Drop‐seqAlignmentCookbookv1.2Jan2016.pdf) from McCarroll Lab. Read pairs with barcode base quality of less than 10 were removed and the remaining reads were then aligned to mouse (mm10) genome using STAR (version 2.5.2a)^41^ with default parameters. Notably, barcodes that are three base‐pair change away from the whitelist were corrected before alignment.

After creation of raw UMI count matrices, further quality control was performed on each cell step by step. First, cells with <500 UMI counts were filtered. Then the background batch genes were subtracted from count matrices.^28^ Finally, potential doublets detected by DoubletFinder (version 2.0.3)^42^ as well as cells with high percentage of mitochondrial genes (>∼5–50%, depending on the dataset) were excluded. Raw sequencing data and the processed differentially expressed gene (DGE) matrix are available from the GEO repository under accession number GSE217664 (Access Token: klwfmkogjfqbful).

#### 2.12.2 Dimension reduction and clustering

The R implementation of package Seurat (version 4.0.5)^43^ was used for analysis of filtered UMI count matrices for each sample. Data was first normalised and log transformed. Then we selected the top 2000 variable genes of each datasets and scaled the expression counts of these genes. Mitochondrial gene counts were regressed out to remove its effect. Next, 50 principal components were calculated and the first 10–40 components were chosen according to ElbowPlot result. For graph‐based clustering, the resolution was set from 0.6 to 1.0, depending on the dataset. Finally, marker genes for each cluster were calculated and each cluster was annotated according to specific markers defined in other literature.

#### 2.12.3 Data integration

The gene expression matrix for all cells, as well as for cells from individual tissues or lineages based on preliminary annotations, was combined and analysed using Scanpy^44^ for clustering. First, we selected the top 2000 to 2500 highly variable genes for downstream analysis and then regressed out mitochondrial gene expressions. Next, we performed PCA and selected the top 20 to 50 principal components (PCs) for dimensionality reduction. A neighbourhood graph was constructed to represent the cellular relationships. For clustering, the Leiden algorithm was applied with a resolution of 2.0. To visualise the data, we applied t‐SNE using the scanpy.tl.tsne function. To identify marker genes for each cluster, the Wilcoxon rank‐sum test was performed using the rank_genes_groups function in Scanpy. Expression data, clustering results, and annotations for each tissue, as well as the combined data, are available on figshare (https://figshare.com/s/6f4ab181391a0c747bb6).

#### 2.12.4 Tissue‐wide differential abundance analysis

We utilised the Milo^45^ framework (https://github.com/emdann/milopy) to assess differences in cell type abundances between p53‐KO and wild‐type mice. To begin, we used milopy.core.make_nhoods with the parameter prop=0.1 to assign cells to its neighbourhoods. Next, we used the function milopy.core.count_cells to quantify the number of cells from each sample within each neighbourhood and then created a cell count matrix where rows correspond to neighbourhoods and columns represent samples. Each neighbourhood was annotated based on a majority voting system, with a neighbourhood labelled as ‘Mixed’ if the most frequent cell type made up less than 60% of the cells in that neighbourhood.

#### 2.12.5 Tissue‐wide cell population similarity analysis

To characterise the extent of response to p53 inactivation, we assessed the similarities of individual cell populations between KO‐pre‐neoplastic and WT tissues using the cosine distance of their transcriptomes.^46^ Initially, we obtained the cell‐by‐gene expression matrix at the single‐tissue level. Subsequently, highly variable genes (HVGs) were identified from each combined matrix to act as input for principal component analysis (PCA) projection. Ultimately, the low‐dimensional space defined by the top 40 principal components (PCs) was utilised to compute the cosine distance between KO‐pre‐neoplastic and WT mice.

#### 2.12.6 G2M, S score and cell cycle proportions calculation

We assigned each cell a score based on gene expression of G2/M and S phase markers by Seurat::CellCycleScoring function and calculated the proportion of each state of individual cell.

#### 2.12.7 RPG expression score, ESC‐like module score, MP module score and entropy score calculation

The RPG sets comprise an assemblage of upregulated RPGs observed across over 50 KO‐pre‐neoplastic cell types at the tissue level, as compared to WT cells. The Myc‐centred embryonic stem cell‐like (ESC‐like) module gene sets were referenced from the research findings reported by Kim et al.^47^ The cancer MP (meta‐program) gene sets were referenced from the research findings reported by Gavish et al.^48^ We used AUCell^49^ package to calculate RPG expression score, MP module score and ESC‐like module score of each cell.

In biology, the term ‘entropy score’ is an indicator that quantifies the differentiation potential of single cells. It serves as a quantified representation of internal cellular disorder and instability. We used CCAT^50^ to measure entropy score of each individual cell.

#### 2.12.8 Copy number prediction with inferCNV

We performed inferCNV (https://github.com/broadinstitute/inferCNV) analysis to extract copy number variations (CNVs) from the single‐cell RNA‐seq data. For CNV inference, we used cells from the WT sample as reference cells. For the run() function, we used the following parameter values: cutoff = 0.1, cluster_by_groups = FALSE, analysis_mode = ‘subclusters’, denoise = TRUE, HMM = TRUE, write_expr_matrix = TRUE, num_threads = 40.

To compute CNV score, we loaded the output gene expression matrix, and the gene expression of cells was re‐standardised and values were limited as –1 to 1. The CNV score of each cell was calculated as quadratic sum of re‐standardised values.^61^ Based on CNV score, we chose top 10 cells with highest CNV score and calculated mean gene expression of these top malignant cells. We used the correlation between gene expression of each cell and mean expression of malignant cells as the threshold for distinguishing malignant cells from normal cells.

#### 2.12.9 Gene Set Variation Analysis (GSVA)

We evaluated biological process activities using a predefined Gene Ontology (GO) dataset of biological processes. To estimate pathway activity for individual cells, we employed Gene Set Variation Analysis (GSVA)^51^ with standard settings, implemented in the GSVA package (version 1.30.0). Differential pathway activities were computed for each cell group downsampled to 500 cells. Significant differential pathways (*p* < .01) were visualised using heatmaps or boxplots displaying the average pathway activity scores for each cell group.

#### 2.12.10 Trajectory analysis of tumourigenesis

Pseudotime trajectory was constructed by Monocle3 R package^52^, ^53^, ^54^, ^55^ in order to reveal tumour progress in T, B, fibroblast and epithelial cells. The normalised data were utilised to construct the Monocle3 object, and UMAP coordinates, computed via the RunUMAP function in Seurat, were integrated into Monocle3. The cluster_cells function facilitated clustering with appropriate resolution. Subsequently, the learn_graph function was employed to delineate differentiation trajectories, with the parameter close_loop = F. The initiation points of these trajectories were automatically identified by Monocle3. While UMAP partially reflects cellular relatedness, excessive cell types can hinder the clustering of closely related cells due to steric hindrance. Hence, for T‐cell lineage data featuring heterogeneous tumour cells, peripheral T‐cell types were individually extracted and re‐clustered to pinpoint the starting point of the tumour development trajectory originating from peripheral T cells. The expression profiles of marker genes in each cell along each trajectory were analysed to characterise gene changes across pseudotime. Heatmaps were partitioned into three or more modules, and the correlation between gene modules and cell clusters was computed and visualised. Genes with a Moran's *I* value > 0.2 within each module were subsequently subjected to gene ontology (GO) analysis.

#### 2.12.11 Transcript factor enrichment

Based on the single‐cell RNA‐seq results, pySCENIC^49^ software was used to infer the regulatory network of transcription factors. We calculated regulons activity for KO and WT datasets separately. For each dataset, we used pseudo‐bulk expression of the normalised expression matrix as input, which compensate for the dropout of genes. Through the analysis, we discovered condition specific regulons and compared differences in the regulatory activity of transcription factors among different lineages between the two conditions.

#### 2.12.12 The ligand‐receptor analysis

CellChat package (version 2.1.0)^56^ was used to investigate the cell‐cell communication signal following the online tutorial.

#### 2.12.13 Single‐cell metabolism quantification

scMetabolism package^57^ was used to investigate the metabolic signal activities following the online tutorial.

### 2.13 scATAC‐seq data analysis

#### 2.13.1 Data preprocessing and quality control

We utilised ArchR^58^ (https://github.com/GreenleafLab/ArchR) for the preprocessing of scATAC‐seq data. Initially, sequencing reads were aligned to the mouse mm10 genome using BWA^59^ (version 0.7.15) with default parameters in single‐end mode. After removing secondary alignments and discordant reads, we used the unique fragments as input to generate Arrow files, incorporating both a TileMatrix and a GeneScoreMatrix. Given the size of the mouse genome, a TileMatrix was constructed with a bin size of 5000 bp. For quality control, single nuclei with fewer than 1000 unique fragments or a TSS enrichment score below 5 were excluded. Doublets were then identified and removed using the ‘addDoubletScores’ and ‘filterDoublets’ functions in ArchR with their default settings. Both raw sequencing data and processed results are accessible through the GEO repository under accession number GSE217664 (Access Token: klwfmkogjfqbful).

#### 2.13.2 Clustering and dimension reduction

For both the overall cell population and individual tissue cells, we performed dimensionality reduction and clustering using the standard ArchR pipeline to identify major cell types. First, we applied the latent semantic indexing (LSI) algorithm via the ‘addIterativeLSI’ function in ArchR. Next, we utilised the ‘addClusters’ function to identify distinct cell clusters, followed by the generation of a two‐dimensional representation using the ‘addUMAP’ function. To identify differentially accessible marker genes for each cluster, we used the ‘getMarkerFeatures’ function with gene scores, which serve as a proxy for gene expression, based on the weighted average accessibility around each gene. The clusters were then annotated primarily using canonical marker genes and tissue‐specific information. All files containing chromatin accessibility data, clustering results, and annotations are available for download from figshare (https://figshare.com/s/6f4ab181391a0c747bb6).

#### 2.13.3 Peak calling, iterative peak merging and DA peak identification

We performed peak calling using the MACS2^60^ callpeak command with the following parameters: –shift 100 –extsize 200 –nomodel –callsummits –nolambda –keep‐dup all. The peaks were filtered using the mm10 blacklist (accession: ENCFF356LFX). To create a union peak set, we combined peaks across all tissue clusters and extended the peak summits by 250 bp. Overlapping peaks were resolved through an iterative process. Initially, we retained the peak with the smallest *p*‐value (defined as the most significant peak) and removed any peak overlapping it. This procedure was then repeated for the next most significant peak, continuing until all peaks were either retained or discarded due to overlap with a more significant peak. To identify differentially accessible peaks, we used the getMarkerFeatures function with the argument useMatrix = PeakMatrix. Finally, these peaks were annotated to the nearest genes.

#### 2.13.4 Motif enrichment in peaks

To explore the variations in functional TFs across different cell clusters, motif enrichment analyses were performed on significant DA peaks of each cluster. Subsequently, the matchMotifs function from the motifmatchr R package (v1.22.0) (https://github.com/GreenleafLab/motifmatchr/) was employed to match motifs against the HOMER collection (http://homer.ucsd.edu/homer/custom.motifs). The enrichment score of each motif was determined by calculating the weighted average of scores across all peaks within each group, with a score of 0 assigned to peaks lacking a specific motif.

#### 2.13.5 Trajectory analysis

We also used ArchR to analyse tumour initialisation of scATAC‐seq data. Function addTrajectory was used to reconstruct cellular trajectory profiling the transition from normal T cells to Tumour cell states. Dynamically accessible regions or functional TFs along pseudo time were computed by getTrajectory function with argument ‘useMatrix = GeneScoreMatrix/ MotifMatrix’, respectively. To identify consistent transition genes between ATAC and RNA data, RNA data derived from the same tumour type were matched to ATAC dataset using function addGeneIntegrationMatrix. Dynamically expressed genes were calculated by getTrajectory with argument ‘useMatrix = GeneIntegrationMatrix’. Shared genes expressed along putative tumour initial progress were obtained using correlateTrajectories and visualised by plotTrajectoryHeatmap.

### 2.14 CUT&Tag data analysis

For Cut&Tag data analysis, we followed the online tutorial (https://yezhengstat.github.io/CUTTag_tutorial/index.html). The paired‐end reads were aligned to the mouse genome build mm10 using Bowtie2^61^ ^(p2)^. The SEACR^62^ software was used for peak identification. Since we didn't have normalised fragment counts with the E. coli read count, we set the normalisation option of SEACR to ‘norm’. R package ChIPseeker (version 1.32.0)^63^ was used to annotate and display the called peaks. Genes observed in two or more biological replicate samples are deemed to exhibit significant binding as target genes with p53 protein. Raw sequencing data and the processed data are available from the GEO repository under accession number GSE217664 (Access Token: klwfmkogjfqbful).

### 2.15 WGS data analysis

We followed MoCaSeq^64^ pipeline (https://github.com/roland-rad-lab/MoCaSeq) to analysis whole genomic sequencing data. For paired p53‐KO and wild‐type samples, we set parameter runmode*=*‘MS’ to compute copy number variation and set parameter Mutect2*=*‘yes’ to detect single‐nucleotide variant. WGS data can be retrieved from figshare (https://figshare.com/s/6f4ab181391a0c747bb6).

### 2.16 Spatial transcriptomics data analysis

#### 2.16.1 Preprocessing of spatial transcriptomics data

For the analysis of two KO thymus samples and one WT thymus sample, we used BSTMatrix 2.0 to process both the FASTQ files and manually aligned histological images. The results were then aligned to the genome using the STAR genome aligner (v2.5.1b). Then we used Seurat v4.0.1 to perform downstream analysis. We applied quality control by filtering out spatial spots with more than 30% mitochondrial gene expression and fewer than 300 detected genes. Additionally, genes detected in fewer than 5 spatial spots were excluded, and spots exhibiting folding were also removed from the analysis.

The intrinsic high cell density of thymic tissue makes it difficult to resolve the cellular identity corresponding to each spot on tissue sections, thereby challenging quality control. To achieve accurate delineation of thymic T‐cell subpopulations, spots were classified as T‐cell‐derived based on CD3 expression criteria (Cd3d > 2, Cd3e > 2, Cd3g > 2). Spots demonstrating low expression for all three CD3 genes (Cd3d < 1.0 and Cd3e < 1.0 and Cd3g < 1.0) were defined as non‐T cells, representing the thymic stromal compartment. More details are listed in Table S9. Raw sequencing data and the processed data are available from the GEO repository under accession number GSE217664 (Access Token: klwfmkogjfqbful).

#### 2.16.2 Dimension reduction and clustering

Raw counts were normalised and log transformed using the “assay=spatial” parameter. Data was first normalised and log transformed. Then the 2000 most variable genes were identified, and the expression levels of these genes were scaled with mitochondrial gene counts regressed out. Next, 50 principal components were calculated and the first 20–40 components were chosen according to ElbowPlot result. For graph‐based clustering, the resolution was set from 0.4 to 1.0, depending on the dataset. Finally, marker genes for each cluster were calculated and each cluster was annotated according to specific markers defined in other literature. Files with expression data, H&E‐stained section (PNG format), clustering, and annotation can be retrieved from figshare (https://figshare.com/s/6f4ab181391a0c747bb6).

### 2.17 Multi‐omics integrative analysis

#### 2.17.1 Integrative analysis based on label transfer strategy

We performed Canonical Correlation Analysis (CCA), implemented in Seurat (v63), to align and correlate cells from scRNA‐seq and scATAC‐seq datasets. This integration procedure aligns cells from the scATAC‐seq data with those from the scRNA‐seq data by comparing the scATAC‐seq gene score matrix with the scRNA‐seq gene expression matrix. To do so, we used the union of the top 2000 most variable genes from each dataset as input for Seurat's “FindTransferAnchors()” function, applying CCA as the reduction method with the parameter k.anchor = 10. For scRNA‐seq data, we identified its nearest neighbour in the scATAC‐seq dataset by performing a nearest‐neighbour search in the joint CCA L2 space. The nearest neighbours were determined using the “FNN” R package (v1.1.3.2, https://cran.r‐project.org/web//packages/FNN/) with the “kd_tree” algorithm based on Euclidean distance. We validated the cell type assignments by comparing the transferred labels from scATAC‐seq with the initial scRNA‐seq annotations. Additionally, we generated a gene expression matrix for the scATAC‐seq cells by adding pseudo‐scRNA‐seq profiles, enabling us to carry out downstream analyses.

#### 2.17.2 Cell type‐specific TFs identification using multi‐omics data

We identified cell type‐specific TFs in whole‐organism main cell types using SCENIC+ package (version 0.1).^49^ For data preprocessing, we used pycisTopic (version 1.0.1) to assign Topics and obtain differential accessible regions (DARs) for each cell type. Then we enriched the TF motifs in the region set by pycisTarget (version 1.0.1). For the scRNA‐seq data side, we preprocessed the data using Seurat (Version 4.1.0). Taking the processed data as input, we created the pseudo‐multi‐omics cells and predicted the regulator TFs based on the concordance of accessible TF binding site, TF expression, and target gene expression.

#### 2.17.3 SNVs mutation mapping to single cells

We adapted the scVarScan^65^ tool to track cell barcode as well as molecular barcode information in scATAC‐seq BAM files. The modified tool was then employed to identify reads that support both the reference and variant alleles at each variant site for every individual cell. For mapping, we utilised high‐confidence SNVs derived from our WGS data.

#### 2.17.4 Prediction of TF‐binding sites

For each considered SNV, the SNV locus that intersects the identified peaks in scATAC‐seq to generate the allele peak set. We searched for known TFBS motifs within allele peaks and reference peaks with the command matchMotifs from the R package motifmatchr, respectively.

#### 2.17.5 Identification of TFs regulating RPGs

For identified p53 direct target genes, those satisfying the following conditions in the corresponding tissues and lineages, were selected as potential transcription factors (TFs) that can regulate RPGs: exhibiting upregulated/downregulated motif scores in scATAC‐seq, up/downregulated RNA expression level and SCENIC regulon scores in scRNA‐seq, and enrichment of the TF motifs in the promoter regions of top RPGs.

#### 2.17.6 Network inference with CellOracle

Cell‐type specific *Trp53* GRN was constructed using CellOracle^66^ according to the official tutorial. We used our single‐cell ATAC data to build base GRN, and then inferred cell‐type specific GRN with single‐cell RNA data. We kept the links between source and target genes with *p*‐value ≤ .001 and weight > 0.002 and selected genes targeted by p53. We intersected these candidate p53 target genes with CUT&TAG result to eliminate false positive results and get final highly confident direct targets.

### 2.18 MouseFormer

To elucidate context‐aware gene‐gene interactions, we employed a model architecture similar to the GeneFormer^67^ model, incorporating four encoder blocks. Each block consists of a self‐attention mechanism followed by a feed‐forward neural network. The model features an input dimension of 1024, with embedding size set to 256, and utilises four attention heads per layer. The feed‐forward network has a hidden size of 512. Self‐attention is applied densely across the entire input dimension of 1024. Key hyperparameters include a ReLU activation function, a dropout rate of 0.02 for all fully connected layers, a dropout ratio of 0.02 for attention weights, weight initialisation with a standard deviation of 0.02, and an epsilon value of 1×10^−12^ for layer normalisation. The model implementation was implemented using PyTorch.

The model was trained following the hyperparameter settings of the GeneFormer framework, with a learning rate capped at 1×10^−3^ and a linear decay schedule incorporating a warmup phase. The Adam optimiser was used and weight decay was set to 0.001. A batch size of 12 was utilised, with 10 000 steps dedicated to warmup. Pretraining was completed after three epochs, taking approximately 6 h, utilising three Nvidia A100 40GB GPUs (a total of 12 GPUs).

After pre‐training, we conducted an in silico perturbation experiment by reducing the expression of *Trp53* to zero. This allowed us to investigate the resulting changes in gene expression and their effects on gene network dynamics at the single‐cell level. The perturbation analysis was conducted following the protocol outlined in the GeneFormer in‐silico perturbation notebook (https://huggingface.co/ctheodoris/Geneformer/blob/main/examples/in_silico_perturbation.ipynb).

## 3 RESULTS

### 3.1 Construction of a *Trp53* functional single‐cell atlas

**FIGURE 1:**
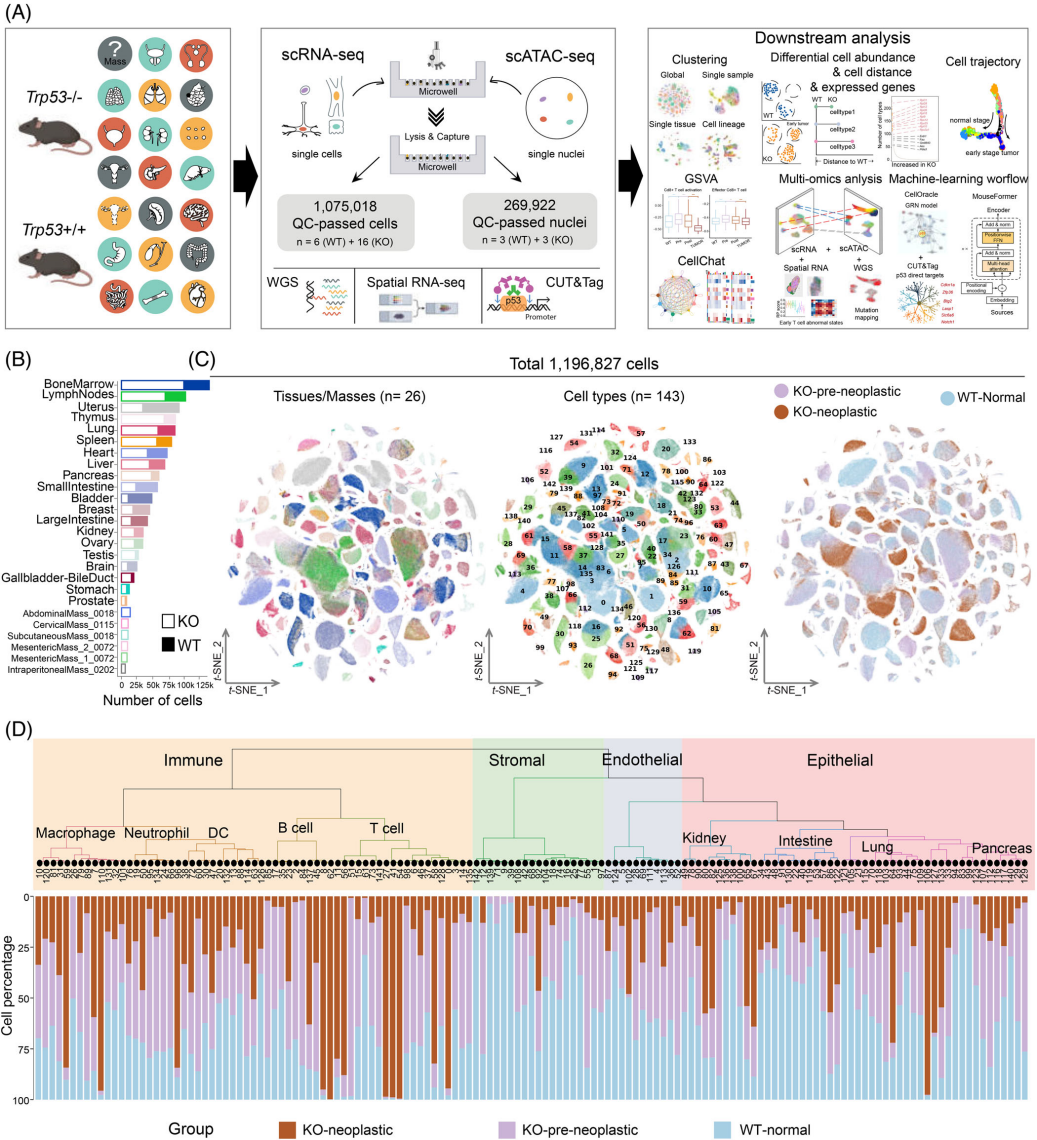
Overview of the *Trp53* functional single‐cell atlas. (**A**) Overview of study design and data analysis workflow. (**B**) Bar plot showing the number of sequenced cells per tissue/mass prepared from wild‐type samples (tissue types from WT mice, *n* = 20) and *Trp53* knockout samples (tissue types from KO mice, *n* = 26). (**C**) *t*‐SNE visualisation of 1 196 827 single cells from KO and WT samples, coloured by (left) tissues/masses, (centre) cell types, and (right) experimental groups (WT, KO‐pre‐neoplastic and KO‐neoplastic). (**D**) Hierarchical clustering dendrogram (top) showing the similarity among 143 cell types, and stacked bar plots(bottom) showing the experimental group composition of each cell type. scRNA‐seq, single‐cell RNA‐seq; scATAC‐seq, single‐cell sequencing assay for transposase‐accessible chromatin; WGS, whole‐genome sequencing; CUT&Tag, Cleavage Under Targets and Tagmentation; WT, wild‐type; KO, knockout Marker genes for each cell cluster are listed in Table S11.

To systematically reveal the function of *Trp53* in various tissues and organs, we generated a comprehensive single‐cell dataset covering the major cell types throughout the body of the *Trp53* knockout (KO) and wild‐type (WT) mice (Figure 1A). We first collected 16 KO mice and 6 WT mice for single‐cell RNA profiling (Figure 1A). Following histological evaluation (Figure S1), within the carefully selected cohort of 16 KO mice, seven individuals (aged 3–5 months) did not manifest an overt tumour phenotype, whereas the remaining nine mice (aged 6–9 months) demonstrated discernible tumours. Accordingly, all KO mice were categorised into two distinct groups: pre‐neoplastic and neoplastic (eight mice harbouring lymphoma, two mice harbouring sarcoma; and one mouse harbouring carcinoma) mice for further analysis (Table S4). This selection closely aligned with the tumour spectrum of the p53 KO model, characterised by a median tumour onset occurring at 4.5 months, with immune tumours being the predominant type, followed by stromal tumours, while epithelial tumours are rarely observed.^1^, ^68^, ^69^, ^70^, ^71^ Concurrently, healthy WT mice of the same strain, aged 3, 5, and 7 months, were selected as the control group for comparison (Table S4). Consequently, these KO and WT mice serve as exemplary research subjects for the exploration of molecular alterations under p53 deficiency and the progression of spontaneous tumourigenesis.

We conducted single‐cell RNA profiling using an updated Microwell‐seq^28^ method with increased cDNA amplification efficiency and improved library quality (Figure S2 and Table S2). The platform reproducibility assessment demonstrated robust sample reproducibility without implementing additional algorithms for batch effect removal (Figure S3). After quality control and filtration, we gathered a total of 1 196 827 single cells derived from twenty tissues and 6 mass samples, namely, small intestine, large intestine, stomach, lymph nodes, spleen, pancreas, heart, lung, thymus, brain, kidney, ovary, uterus, bladder, mammary gland, liver, gallbladder‐bile duct, bone marrow, testis, prostate, intraperitoneal mass, mesenteric mass1, mesenteric mass2, cervical mass, abdominal mass, subcutaneous mass (Figure 1B and Table S5).

Using a shared‐nearest neighbour (SNN)‐based unsupervised algorithm, the cells were grouped into 143 major clusters (Figure 1C), which were precisely annotated based on marker gene expression (Figure S4 and Table S11). Totally, 143 major clusters were divided into 9 main cell lineages, including epithelial, endocrine, stromal, endothelial, immune, erythroid, hepatocyte, neural, and germ cells. The populations within the epithelial lineage (*Krt19* and *Krt8*) encompass certain cell types endowed with secretory functions, including some ductal cells (*Clu* and *Spp1*), luminal cells (*Wfdc2* and *Calb1*), glandular cells (*Anpep* and *Padi1*), intestinal goblet cells (*Muc2* and *Zg16*), and ovarian granulosa cells (*Cyp19a1* and *Slc18a2*). The stromal lineage comprises subgroups such as fibroblasts (*Dcn* and *Col1a1*), muscle cells (*Myh11* and *Myl9*), and adipocytes (*Cfd* and *Adipoq*). The endothelial lineage encompasses conventional arterial endothelial cells (*Sox17* and *Sema3g*), venous endothelial cells (*Vwf* and *Selp*), capillary endothelial cells (*Cd36* and *Fabp4*), as well as specialised liver sinusoidal endothelial cells (*Clec4g* and *Stab2*). Immune cells consist of multiple cell clusters. Cluster 45 (C45) was defined as pre‐B cells according to *Vpreb1* and *Vpreb3*. C20, C130, C132, C138 and C142 were defined as plasma cells according to *Jchain* and *Igkc*. Finally, C2, C17, C23, C35, C84, C85 and C95 were designated as B cells exhibiting mature antigen‐presenting functionality, distinguished by *Cd79a* and *Cd19*, with elevated expression levels of *Cd74* and MHC class II molecules. T cells are also composed of multiple subclusters, including double‐positive T (DP T) cells (*Cd8a* and *Cd4*), alpha beta T cells *(Trac* and *Trbc1*), Cd8^+^ T cells (*Cd8a* and *Cd8b1*), Cd4^+^ T cells (*Cd4* and *Icos*), gamma delta T cells (*Tcrg‐C1* and *Trdc*), and T cells (*Cd3e* and *Tcf7*). Specifically, with high expression of *Mpo* and *Prtn3*, C38 and C72 were defined as granulocyte‐monocyte progenitors (GMPs). In addition, we identified five myeloid groups on the basis of their transcriptome profiles, that is, dendritic cells (*Cst3* and *Cd209a*), macrophages (*C1qc* and *Ctss*), mast cells (*Cpa3* and *Tpsb2*), monocytes (*Lyz2* and *Itgam*), and neutrophils (*S100a8* and *S100a9*). The cell‐type hierarchy tree showed that cell clusters from the same cell lineage tended to converge together beyond the functional impact of p53 deficiency, especially for immune, stromal, endothelial, and epithelial cells (Figure 1D), suggesting the absence of significant developmental anomalies in KO mice.

### 3.2 Cross‐lineage tumourigenesis and progression in response to p53 deficiency

To concentrate on the identification of tumour cell clusters and delineate the oncogenic characteristics across diverse cellular lineages, we integrated and reclustered the scRNA‐seq data at the lineage level to construct the cross‐tissue maps for independent analysis (Table S12). By cell proportion calculation under WT and KO stages in each cluster, coupled with the immunohistochemical findings from tissue specimens and copy number variation (CNV) analysis of the samples (Figure S5), we confirmed the presence of tumours in T, B, fibroblast, and mammary epithelial cells. To elucidate the alterations across distinct stages of tumourigenesis, all WT and KO cells were divided into WT‐Normal (WT), KO‐pre‐neoplastic (Pre), and KO‐neoplastic (Post) groups based on the mouse groups for further analysis. To better discern tumourigenic changes and the impact of tumour‐bearing, cells at the Post stage were split further into the neoplastic‐tumour (Post‐T) cells, and neoplastic‐nontumour (Post‐NT) cells encompassing non‐malignant cells but possibly impacted by the tumours.

**FIGURE 2:**
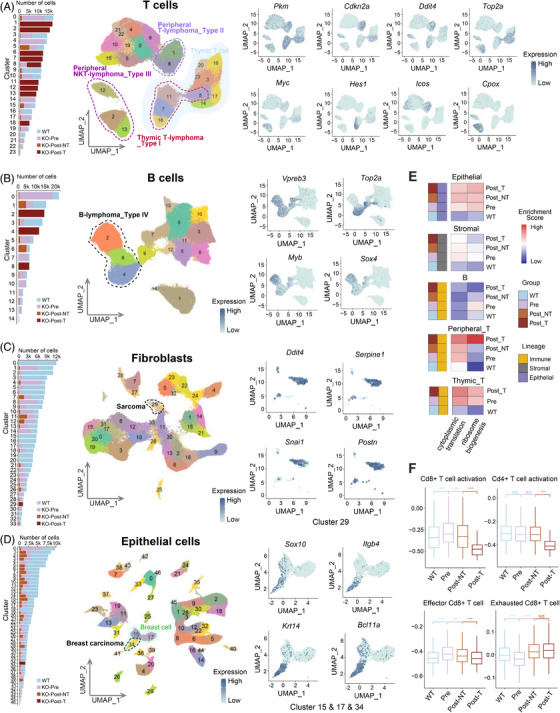
Significant changes of different cell lineages with tumourigenesis in response to *Trp53* KO. (**A**) Global clustering of T cells. (left) Bar plot of cell counts per T‐cell subcluster across experimental groups (WT, KO‐pre‐neoplastic, KO‐neoplastic‐Tumour, and KO‐neoplastic‐NonTumour). UMAP of all reclustered T cells coloured by cell subclusters (centre). Representative marker genes of distinct T‐cell lymphomas (right). (**B**) Global clustering of B cells. (left) Bar plot of cell counts cells per B‐cell subcluster across experimental groups. UMAP presentation of all reclustered B cells coloured by cell subclusters (centre). Representative marker genes of distinct B‐cell lymphomas (bottom). (**C**) Global clustering of fibroblasts. (left) Bar plot of cell counts per fibroblast subcluster across experimental groups. UMAP presentation of all reclustered fibroblasts coloured by subclusters (centre). Representative marker genes in sarcoma cells (C29) (right). (**D**) Global clustering of epithelial cells. Bar plot of cell counts per epithelial cell subcluster across experimental groups (left). UMAP presentation of all reclustered epithelial cells coloured by subclusters (centre). Representative mark genes in breast carcinoma cells (C34) (right). (**E**) Heatmap showing GSVA scores of Gene Ontology (GO) Terms (cytoplasmic translation and ribosome biogenesis) for cell lineage at different states. (**F**) Boxplots showing GSVA scores of cellular functional activity intensities of Cd8^+^ and Cd4^+^ T cells at different stages. WT, wild‐type; KO, knockout. Marker genes for each cell cluster are listed in Table S12.

In the global T‐cell atlas, we identified 23 clusters, categorised into two principal groups: thymic T cells and peripheral T cells (Figures 2A and S6A and B). Following copy number analysis with inferCNV, we found that 9 cell clusters at Post stage showed enriched predicted aneuploidy cells (Figure S6C). These clusters exhibited three distinct tumourigenesis expression signatures, each corresponding to a subtype of T‐cell lymphoma. C8, C11, C7, and C18 were proximal to double‐positive T cells, representing thymic lymphoma characterised by elevated expression levels of genes like *Notch1*, *Hes1* and *Desi1*,^72^, ^73^ along with markers associated with thymic T‐cell development such as *Rag1/2*, *Xrcc6* and *Dntt* ^74^ (Figures 2A and S6B). Conversely, C1 and C6 denoted peripheral T‐cell tumours displaying heightened expression of *Icos* and *Swi5* ^75^, ^76^ (Figures 2A and S6B). C12, C2, and C3 indicated *Cd3d* ^+^ *Klrg1* ^+^ Natural killer T (NKT)‐cell lymphoma,^77^ demonstrating tumourigenesis features including *Cpox* and *Pvt1* ^78^, ^79^ (Figures 2A and S6B). Moreover, these T‐cell tumours manifested shared molecular characteristics. The tumour suppressor gene *Cdkn2a*,^80^ DNA damage repair gene *Ddit4*,^81^ oncogene *Myc*,^82^ and metabolism‐associated gene *Pkm* ^83^ exhibited markedly heightened expression levels in these tumour cells (Figure S6B). The marker genes of T‐cell lymphomas were enriched across analogous pathways, encompassing cell cycle modulation, regulation of apoptosis, maintenance of stemness, metabolism of energy substrates and macromolecules, chromatin remodelling, and response to oxidative stress. Remarkably, p53 deficiency triggered dynamic shifts in gene expression patterns within both thymic and peripheral T cells. At the Pre stage, we found that thymic T cells showed the upregulation of pathways associated with ribosome biogenesis, assembly, and the biosynthesis and modification of biomacromolecules (Figure S6D), and exhibited downregulation of genes such as *B2m*, *Lck*, *Nfatc3*, and *Satb1*, which aligns with T‐cell developmental processes such as TCR biogenesis, positive and negative selection^84^, ^85^, ^86^, ^87^ (Figure S6E and F). At the Pre state, p53‐deficient peripheral T cells initially displayed elevated expression of ribosomal genes and T‐cell activation‐related genes including *Ifi47*, *Ifi213*, *Irf7* and *Il16* ^88^ (Figure S6F and G). Subsequently, at the Post‐NT state, these cells displayed increased synthesis of T‐cell suppressive factors such as *Tnfaip3*, *Ccl5* and *Ctla4*,^89^, ^90^, ^91^ coinciding with tumour emergence (Figure S6F).

In the global B‐cell atlas, we discerned three KO‐dominated clusters (C2, C4, and C8) that showed weak antigen‐presenting cell (APC) function and mainly highly expressed pre‐B cell receptor genes such as *Vpreb3* and *Vpreb1* ^92^ (Figures 2B and S6H and I). Through the detection of markers like *Myb* and *Sox4*,^93^, ^94^ and the calculation of CNVs, these three clusters with malignancy characteristics were identified as B‐cell lymphoma (Figure S6I and J). Moreover, these tumour cells displayed heightened expression of genes associated with early B‐cell development, including *Rag1/2*, *Endou*, and *Dntt*.^92^ Of note, other representative genes of B‐cell lymphoma, such as *Cdkn2a* and *Pkm*, *Ldha* (Figure S6I and K), mirrored the expression profiles observed in thymic T‐cell lymphomas. Furthermore, aside from the upregulation of ribosome‐related pathways, Pre‐stage‐specific peripheral B cells also manifested a substantial increase in antigen presentation activity (Figure S6L). For other immune cells such as myeloid cells, the predominant response involved the upregulation of pathways associated with immune cell migration and inflammatory responses (Figure S7A). Moreover, Pre‐stage‐specific macrophages exhibited aberrant hyperactivity in antigen presentation, characterised by the overexpression of key genes including *Cd74*, *H2‐Eb1*, and *Fcer1g* (Figure S7B and C).

In the global fibroblast atlas encompassing diverse organs, we identified 33 distinct clusters (Figures 2C and S7D). Notably, cluster C29, derived from mouse KO0115 and KO0018, exhibited the highest CNV score and heightened expression of genes including *Ddit4*, *Serpine1*, *Snai1*, and *Postn* ^95^, ^96^, ^97^, ^98^ (Figure S7E). Since these genes are implicated in fibroblast proliferation, collagen synthesis, angiogenesis, and pivotal signalling pathways such as Wnt and TGF‐β (Figure S7F), C29 was characterised as a cluster comprising malignant sarcoma cells. Furthermore, apart from the enrichment of genes associated with ribosomal reactions, Pre‐stage‐specific fibroblasts displayed the upregulation of genes associated with mesenchymal development and immune regulation pathways, exemplified by *Fau*, *Rpl18*, *Mdk*, and *Nr1d1* (Figure S7G and H).

The histological heterogeneity among epithelial cells was notably pronounced (Figures 2F and S7I and J). Specifically, C34 was classified as malignant breast epithelial carcinoma cells with the highest CNV score, manifesting conspicuous traits of epithelial‐mesenchymal transition (EMT) and a proclivity towards myoepithelial cell conversion (Figure S7K and L). These cells showed intense expression levels of key genes including *Sox10*, *Itgb4*, *Krt14*, and *Bcl11a* ^99^, ^100^, ^101^, ^102^ (Figure 2F). In addition, functional alterations within Pre‐stage‐specific epithelial cells predominantly involved upregulation of ribosomal pathways and perturbations in stress‐activated cascades and immune regulation‐related pathways (Figure S7M). In general, we found that the tumour cells maintained discernible characteristics across different cell lineages, primarily marked by critical genes and pathways linked to the biomolecule genesis, assembly, processing and metabolic, cell death, and cellular stress response (Figure S8A).

To further validate the aforementioned changes in response to the absence of p53 and explore the collective alterations in multisystem coordination, we integrated data from various lineages at different stages to examine variations in the activity of characteristic signalling pathways and intercellular communication. Firstly, we observed an anticipated augmentation in the activity of cytoplasmic translation and ribosome biogenesis across diverse cell lineages in p53‐deficient cells (Figure 2E). Secondly, for thymic T cells predisposed to tumourigenesis, we observed a sustained and significant downregulation of homeostatic pathways (Figure S8B), suggesting that the disruption of homeostasis undoubtedly poses a lethal risk to the thymus characterised by a high proportion of proliferating cells. Thirdly, in the realm of immune activity, there was a notable attenuation in the antigen response of thymic T cells (Figure S8C). Conversely, peripheral T, B, myeloid, stromal, epithelial, and endothelial cells demonstrated heightened engagement in antigen presentation pathways, particularly during the Pre stage (Figure S8C and D). The reinforcement of antigen receptor‐mediated immune signalling could be used to explain the activation of peripheral mature T cells and the instigation of inflammatory response (Figure S8E–G). The enhanced protein synthesis and diminution of catabolic processes during the pre‐stage could also potentially promote the activation of immune cells^103^ (Figure S8H). Upon closer examination of T‐cell alterations, it was noted that peripheral cytotoxic Cd8^+^ T cells demonstrated peak activity during the Pre stage, displaying a predilection towards effector T‐cell traits (Figure 6J). Afterwards, they exhibited a transition towards exhausted T‐cell phenotypes in the Post stage, coinciding with a progressive decline in the activity of both Cd8^+^ and Cd4^+^ T tumour cells (Figure 2F). Furthermore, the examination of cellular interactions corroborated the consistency of the results. Within the KO thymic environment, naive T cells displayed diminished antigen presentation and incoming signalling crucial for their normal developmental processes (Figure S9A), while mature T cells within lymph nodes and other parenchymal organs experienced heightened signalling through MHC molecules (Figure S9B–D). The analysis of ligand‐receptor interactions in tumour stage unveiled distinct crosstalk between T cells and myeloid cells, B cells, and tumour cells, featuring enhanced engagement of immune inhibitory or immune checkpoint synergistic pathways such as VEGF, CXCL and GALECTIN^104^, ^105^, ^106^ (Figure S9E–N).

The above results collectively indicated that for cell lineages with tumourigenesis, increased RPG expression and enhanced protein synthesis emerge as common themes of alteration, which are accompanied by attenuated molecular responses associated with thymocyte development and intensified systemic inflammatory responses orchestrated by immune and parenchymal cells. These findings implied that lineage‐common and lineage‐specific regulatory features contribute jointly to multi‐lineage tumourigenesis and progression.

### 3.3 Pseudo‐temporal dynamics of tumourigenesis in T, B, stromal, and epithelial cells

**FIGURE 3:**
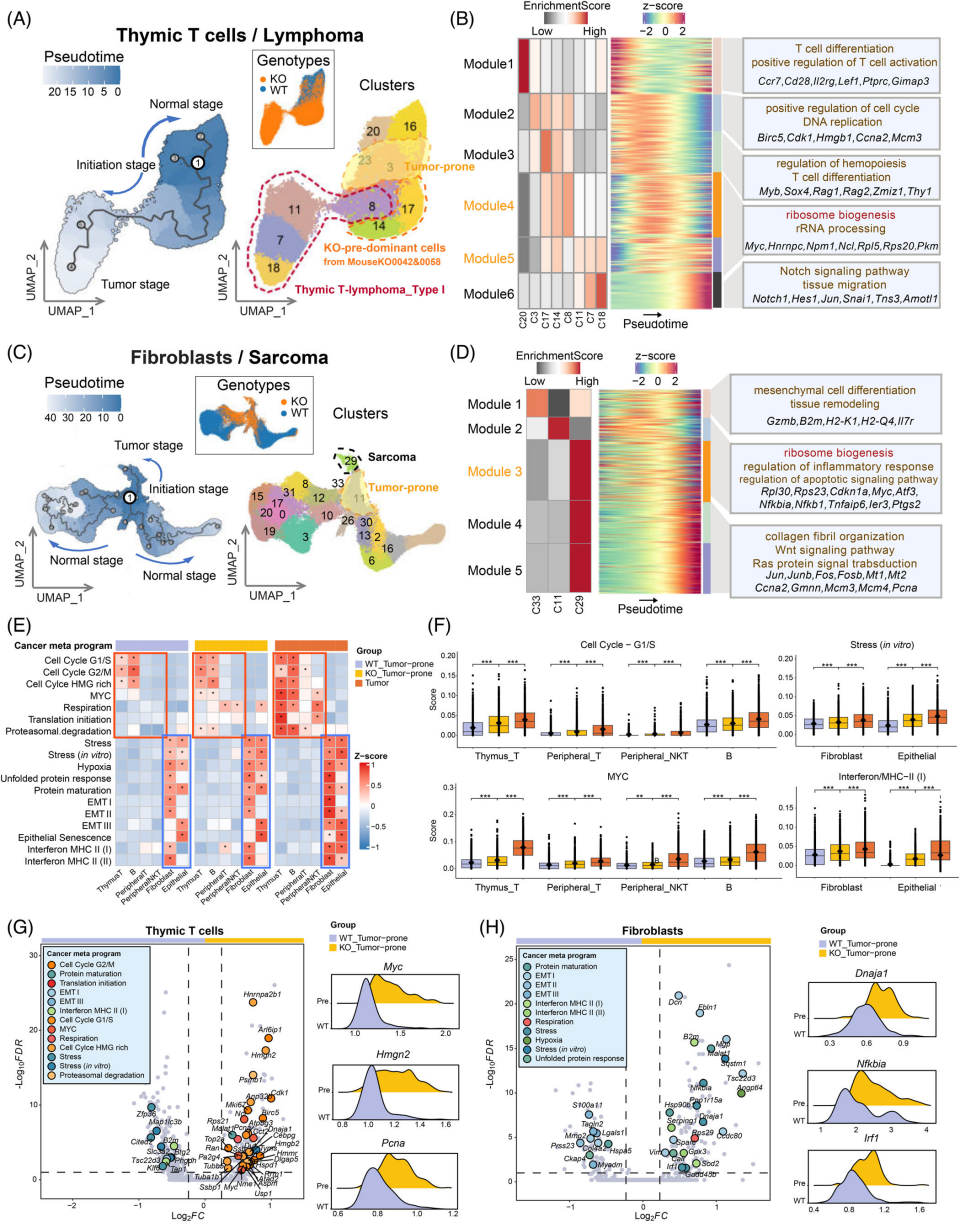
Reconstruction of the tumourigenesis trajectory following p53 inactivation. (**A**) Pseudotemporal reconstruction of the thymic T‐cell lymphomagenesis trajectory. UMAP visualisation of thymic T cells, coloured by pseudotime (bottom‐left), cell subclusters (bottom‐right) and genotypes (top), respectively. (**B**) Heatmap showing the expression scores of identified pseudotemporal gene modules for different thymic T‐cell subclusters (left) and branch‐dependent gene expression patterns in pseudotime of thymic T‐cell lymphomagenesis trajectory (middle). Marker genes and enriched GO terms of branch‐dependent genes are shown on the right panel. (**C**) Pseudotemporal reconstruction of the fibroblast sarcomagenesis trajectory. UMAP visualisation of fibroblasts, coloured by pseudotime (bottom‐left), cell subclusters (bottom‐right) and genotypes (top), respectively. (**D**) Heatmap showing the expression scores of identified pseudotemporal gene modules for different fibroblast subclusters (left) and branch‐dependent gene expression patterns in pseudotime of fibroblast sarcomagenesis trajectory (middle). Marker genes and enriched GO terms of branch‐dependent genes are shown on the right panel. (**E**) Heatmap showing the cancer meta program (MP) gene module scores of different tumour‐prone cell groups across different lineages (asterisks indicate *Z*‐scores >0.15). (**F**) Boxplot showing the cancer meta program gene module scores of different tumour‐prone cell groups across different lineages. (**G**) Volcano plot showing the differential expressed genes between WT and KO‐pre‐neoplastic thymic tumour‐prone T cells (left, coloured by enriched MP modules). Ridge plot showing accessibility scores of gene regions of WT/KO‐pre‐neoplastic cells (right). (**H**) Volcano plot showing the differential expressed genes between WT and KO‐pre‐neoplastic tumour‐prone fibroblasts (left, coloured by enriched MP modules). Ridge plot showing accessibility scores of gene regions of WT/KO‐pre‐neoplastic cells (right).

To recover the transcriptional program during multi‐lineage tumourigenesis and investigate shared regulatory dynamics across different lineages in response to p53 deficiency, we ordered cells from individual lineages employing Monocle3^52^, ^55^ (Figures 3A–D and S10).

In the case of thymus‐derived T cells extracted from global T cells (Figure S6C), thymic lymphomas manifested a discernible tumourigenic trajectory (Figure 3A) – the pathways of normal development (Figure 3B Module 2 and 1) and tumour progression (Figure 3B Module 2–6) distinctly delineated opposing directions of advancement (Figure 3A and B). At the onset, robust cellular proliferation and DNA replication were evident in thymic CD4^+^CD8^+^ double‐positive T cells (Figure 3B Module 2). In the initial phases of the deviant trajectory, there persisted an enrichment of genes associated with T‐cell development, T‐cell differentiation and haematopoiesis (Figure 3B Module 3). Subsequently, there was an enhancement of functionalities linked to ribosomal biogenesis and RNA processing (Figure 3B Module 4 and 5) observed in Pre cells from C14 and C17, and malignant cells. Finally, there was a pronounced upregulation of genes related to Notch signalling and tissue migration signals (Figure 3B Module 6).

The pre‐B cell lymphomagenesis in the bone marrow exhibited similarities to that observed in thymic T cells, displaying two distinct trajectories (Figure S10A): one for normal (Figure S10B Module 3–1) and the other for aberrant (Figure S10B Module 3–6). The initial stage of malignant cell development was marked by the enrichment in ribosome biogenesis and RNA processing (Figure S10B Module 4 and 5), ultimately leading to the emergence of invasive pathways (Figure S10B Module 6). However, the trajectories of the two non‐thymic T‐cell populations demonstrated significant tumourigenesis heterogeneity compared to thymic T cells (Figure S10C–F). Beyond the consistent dysregulation of RPGs and uncontrolled cell proliferation (Figure S10F Module 2 and 3), peripheral T‐cell lymphoma at the initial stage mainly involved the signals related to inflammation, immune response, cytokine mediation, and T‐cell activation (Figure S10F Module 1).

Sarcomas arose without tissue specificity, originating from a diverse spectrum of mesenchymal stem cells (Figure 3C). In the early stage of sarcoma development, similar to T cells, there were modest alterations in the expression of RPGs, alongside an upregulation of p53 target genes such as *Cdkn1a*, *Myc* and *Atf3* (Figure 3D Module 2–5). Concurrently, there was a notable elevation in the level of activator protein 1 (AP‐1) family genes (*Jun*, *Junb*, *Fos* and *Fosb*) and metallothionein genes (*Mt1* and *Mt2*) (Figure 3D Module 3–5). Moreover, for early sarcomagenesis, functional changes were evident in cell death, proliferation, and stress‐mediated pathways, accompanied by heightened inflammatory signalling (Figure 3D Module 2–3). Subsequently, as it progressed to the late stage, there was a robust activation of Wnt signalling, anti‐apoptotic signals, and vigorous assembly of collagen (Figure 3D Module 4–5). Carcinoma was specifically derived from secretory alveolar (AV) cells within the mammary gland, which are recognised as luminal progenitor (LP) cells^107^ (Figure S10G). Besides the lineage‐shared enhanced expression of RPGs, the initial phase of breast carcinoma engaged apoptosis, inflammatory response, and stimuli‐related signalling (Figure S10H Module 2–3). Subsequently, the later stages primarily featured the enrichment of AP‐1 family genes, and activation of Epithelial‐Mesenchymal Transition (EMT) and Wnt signalling pathways (Figure S10H Module 4–5). Notably, the diverse modifications in signalling pathways during tumourigenesis could potentially contribute to the delayed onset phenomenon observed in *Trp53* KO‐triggered stromal and epithelial tumourigenesis.

Overall, the developmental trajectories of tumours feature modules related to ribosomal pathways, which are indicative of key molecular events during tumourigenesis. By integrating pseudotime information, we can infer the initial identity of tumour cells from different lineages. Thymic T‐cell lymphomas are born from proliferating double‐positive T cells; peripheral T‐cell lymphomas derive from immune‐activated T cells and NKT cells; B‐cell lymphomas derive from proliferating pre‐B cells; sarcomas derive from pan‐tissue *Nfkbia* ^+^ fibroblasts; epithelial carcinomas are traced back to secretory AV cells in the mammary gland. We classified these initial cells as tumour‐prone populations upon p53 inactivation. Our findings revealed that the tumour‐prone cell populations across all lineages exhibited highly distinct signatures, which were associated with the oncogenic competence of these cell types to initiate cancer upon acquisition of the p53‐loss driver lesion (Figure S11A). Thymic T and B tumour‐prone cells displayed unique features of lymphocyte development, involving physiological processes such as V(D)J recombination. Peripheral T tumour‐prone cells were characterised by immune response‐related pathways. Furthermore, all tumour‐prone cells showed alterations in p53‐associated regulatory molecules compared to other cells within the same lineage. Consistent with the pseudotime analysis, peripheral T, fibroblastic and epithelial tumour‐prone cells at KO state exhibited activation of inflammation‐related pathways, including the TNFα, NF‐κB, hypoxia, and JAK‐STAT signalling pathway (Figure S11B). Epithelial KO tumour‐prone cells demonstrated a propensity for EMT (Figure S11C).

To characterise the gene transition patterns associated with p53 inactivation‐driven heterogeneity in tumours, we analysed changes in hallmark gene sets of recurrent malignancy heterogeneity previously identified in pan‐cancer samples^48^(Figures 3E–H and S12). As expected, consensus malignant cell gene modules were most enriched in tumour cells (Figure 3E and F). KO tumour‐prone cells across different lineages exhibited an intermediate transitional state between normal cells and malignant cells (Figure 3E and F), highlighting the broad influence of p53 on tumour heterogeneity across diverse cancer types. Notably, significant differences in module activity were observed between distinct cell lineages (Figures 3E and F and S12A and B). Immune cells showed stronger associations with cell cycle, proto‐oncogene targets, cellular respiration, and translation initiation modules, whereas stromal and epithelial cells were more enriched for hypoxia, stress response, interferon signalling, and EMT modules. Strikingly, the overall differences in module activity between normal and tumour cells were less pronounced than lineage‐specific variations, suggesting that much of the p53 loss‐driven tumour heterogeneity observed in cancer cells faithfully reflects the biology of their cell of origin. Further investigation revealed upregulated and downregulated tumour hallmark genes in p53‐deficient tumour‐prone cells (Figures 3G and H and S12C), which were closely linked to the establishment of malignant cellular states. Additionally, a subset of these genes exhibited coordinated changes in chromatin region openness and expression levels (Figure 3G and H), indicating that epigenetic chromatin background alterations synergistically influence transcriptional regulation.

### 3.4 Identification of characteristic thymic tumour‐initiating T cells with chromatin organisational defect

**FIGURE 4:**
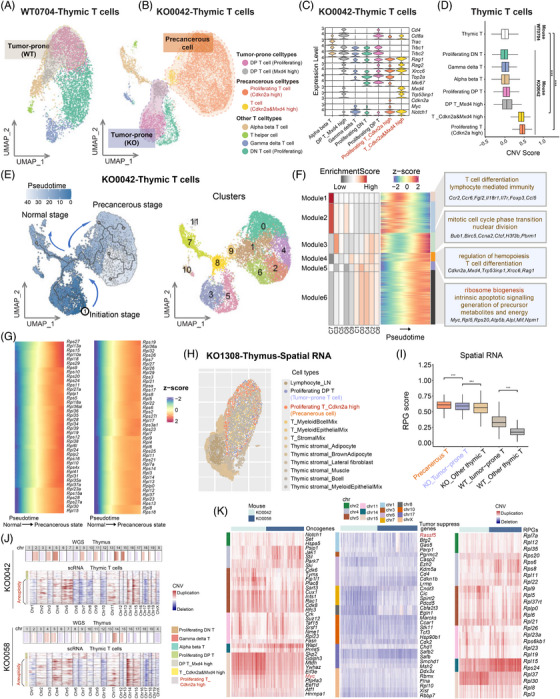
Characterisation of tumour‐prone thymic T cells under p53 inactivation. (**A**) UMAP visualisation of thymic T cells from mouse WT0704, coloured by cell subtypes. (**B**) UMAP visualisation of thymic T cells from mouse KO0042, coloured by cell subtypes. (**C**) Violin plots showing representative marker genes of thymic T‐cell subtypes from mouse KO0042. (**D**) Box plots showing copy number variation (CNV) scores of different thymic T‐cell subtypes from mouse KO0042/WT0704. (**E**) Pseudotemporal reconstruction of the thymic early T‐cell tumourigenesis trajectory of mouse KO0042. UMAP visualisation of the trajectory, coloured by pseudotime (left). UMAP visualisation of thymic T cells, coloured by cell subclusters (right). (**F**) Heatmap showing the expression scores of identified pseudotemporal gene modules for different thymic T‐cell subclusters of mouse KO0042 (left) and branch‐dependent gene expression patterns in pseudotime of early thymic T‐cell tumourigenesis trajectory (middle). Marker genes and enriched Gene Ontology (GO) terms of branch‐dependent genes are shown on the right panel. (**G**) Heatmaps showing the progressive upregulation of ribosomal protein genes (RPGs) during the early tumourigenesis of thymic T cells from mouse KO0042. (**H**) Spatial projection of KO‐pre‐neoplastic mouse thymus section, coloured by cell types. (**I**) Boxplot showing the RPG expression score of T‐cell subclusters using spatial transcriptomics data. RPGs analysed were those shown in Figure 4G. (**J**) CNV plot of thymic T cells from mouse KO0042 (top)/KO0058 (bottom) employing inferCNV. Bottom labels indicate different chromosome regions and left colour bars indicate different cell subclusters. (**K**) Heatmap showing the copy number variations of oncogenes (left), tumour suppress genes (middle) and RPGs (right) enriched in chromatin amplification or loss regions from KO0042/KO0058 mouse cells. WT, wild‐type; KO, knockout.

To further elucidate the initial oncogenic transformation induced by p53 deficiency, we investigated the nuanced early‐stage tumourigenesis of thymic T‐cell lymphomas characterised by high penetrance and fixed lesions (Figures 4 and S13). We noted that C14 and C17, which primarily originated from mouse KO0042 and KO0058 in the Pre group, exhibited functional enrichment modules similar to the malignant cells (Figure 3B Module 4), serving as the crucial connection between the WT and lymphoma cell clusters within the tumour progression trajectory. We hypothesised that C14 and 17 might represent a transitional precancerous state between normal and malignant cells. Naturally, we performed trajectory analysis on T cells in the thymus from mouse KO0042 and KO0058 respectively, aiming to elucidate the timing and mechanisms underlying the divergence of T cells into distinct trajectories and delineating the genetic contributions (Figures 4E and F and S13A–C). Expectedly, T cells from the two mice displayed markedly similar differentiation patterns. Consistent with WT mice (Figure 4A), the transition from proliferating CD4^−^CD8^−^ double‐negative T (DN T) cells to CD4^+^CD8^+^ double‐positive T cells progressed in a typical manner along the normal developmental path (Figure 4B and C). We found that the double‐positive T‐cell subgroup acted as a pivotal branching point, characterised by elevated expression levels of the homeostatic maintenance genes *Mxd4* and *Trp53inp* associated with V(D)J rearrangement essential for T‐cell receptor (TCR) generation^74^ (Figure 4B and C). Along the other abnormal branch, upon the upregulation of DNA recombination‐related genes such as *Xrcc6*, *Rag1* and *Rag2*.^108^, ^109^ growth‐proliferation cell cycle‐associated genes such as *Myc* and *Notch1*,^110^ cancer‐associated genes such as *Cdkn2a* and *Alpl*, absence of TCR genes such as *Trac* and *Trbc1/2* (Figure 4B) and elevated RPG expression, a distinct subset of precancerous T cells emerged (Figures 4B–F and S13A–D). Moreover, this population of precancerous cells shows elevated expression of tumour suppressor genes such as *Cdkn2a*, alongside upregulation of apoptotic pathways, suggesting a cellular response to the stresses associated with p53 loss during the early stages of tumourigenesis (Figure 4F Module 6 and Figure S13C). We identified a cohort of 80 RPGs that were upregulated synchronously with tumour progression in a pseudotime‐shifted manner, indicative of their role as initiating features in tumourigenesis (Figures 4G and S13D). As expected, these 80 RPGs exhibited the highest expression levels within this *Cdkn2a* ^+^ precancerous cell population (Figure S13E).

To spatially depict the early carcinogenic predispositions triggered by *Trp53* KO and characterise the dynamics of gene expression and cellular interactions, we conducted spatial transcriptomic analyses on thymus samples from KO‐pre‐neoplastic and WT mice (Table S9). We obtained 11 841 cells from the KO sample with a mean of 1555 genes per spot and a mean of 2633 UMI per spot with 27 µm resolution, and 30 269 cells from the WT sample with a mean of 977 genes per spot and a mean of 1371 UMI per spot. In the thymus spatial transcriptome landscape, the main cell types were identified, including T cells and thymic stromal cells (B cells, myeloid cells, epithelial cells, fibroblasts, muscle cells and erythroid cells) (Figures 4H and S13F and G). Of note, spatial transcriptome data supported *Trp53* KO‐induced tumour initiation programs. Consistently, we noted the *Cdkn2a* ^+^ precancerous T‐cell subset exhibited elevated expression scores of the aforementioned identified 80 RPGs and heightened expression of *Xrcc6*, *Dntt* and *Rag1* in the KO samples (Figures 4I and S13G). The observed aggregation of this atypical T‐cell subset predominantly residing in the peripheral zone of the thymus was suggestive of an immature functional development. This observation underscored a notable resemblance to the anomalous clusters identified through scRNA‐seq analysis (Figure S13H). In situ analysis of cellular communication also concurred with the findings of scRNA data. For example, the enhanced pathways like MK, PARs, MIF, and PTN, attenuated LCK‐coordinated TCR biogenesis pathways,^85^ along with decreased activities of MHC class I and II molecule presentation (Figure S13I–K), exhibited a remarkable alignment with scRNA data (Figures S6F and S13K) and the preceding Gene Set Variation Analysis (GSVA) and CellChat analysis (Figures S8 and S9). Collectively, spatial transcriptomic data characterise the spatial distribution of the precancerous T‐cell subsets and confirm the aberrant activity of RPGs and other molecular pathways.

Relative to tumour‐prone cell populations, this cohort of tumour‐initiating cells exhibits a markedly increased mutational burden, displaying features of aneuploidy. The chromosomal aberration patterns in thymic T cells from MouseKO0042 and MouseKO0058 display notable homogeneity, characterised by gains on chromosomes 4, 5, 14, and 15, as well as losses on chromosomes 6, 10, and 17 (Figure 4J). Furthermore, these chromatin‐altered regions encompass amplifications of key oncogenes such as *Myc* and deletions of tumour suppressor genes such as *Rassf5* (Figure 4K), thereby furnishing additional mutational drivers for tumourigenesis. These alterations indicate the ordered and deterministic nature of cancer genome evolution following p53 inactivation.^111^ Moreover, increased copy numbers of RPGs on amplified chromosomes were observed (Figure 4K), suggesting that chromatin structural variations synchronously influence gene expression.

### 3.5 Epigenetic signatures of p53 loss‐mediated transformation

To determine the contribution of epigenetic transformation in response to p53 loss, we constructed an optimised Microwell single‐cell sequencing assay for transposase‐accessible chromatin (scATAC‐seq)^112^ (Figure S14A and Table S3). Human‐mouse cell mixing experiment indicated a minimal contamination rate on this platform, coupled with favourable sensitivity and fragment capture capabilities (Figure S14B and C). Using this novel technique, we collected chromatin accessibility data from three p53‐deficient mice and three WT mice (Figure S15 and Table S5). A total of 288 242 individual nuclei across 19 tissues and 2 mass samples passed quality control filtration (Figure S15A and B). We first performed iterative clustering with the ArchR^58^ package and classified the 288 242 nuclei into 35 major cell clusters (Figure S15B and C). Next, we annotated these cell clusters on the basis of gene activity scores compiled for chromatin accessibility across a gene's regulatory region (Figure S15D). The 35 major cell clusters covered major cell lineages including stromal, neuron, hepatocyte, erythroid, epithelial, and immune cells. To understand the correspondence between chromatin and the gene expression landscape, we used a label transfer approach^43^, ^58^ to link scATAC‐seq clusters to cell clusters in the scRNA‐seq atlas. We observed a strong correlation between gene scores and RNA expression values, as well as concordance between cell‐cluster labels (Figure S15E), further supporting our annotations. At the level of specific lineages, ATAC data also exhibited a strong correspondence with RNA data, both in terms of cellular population mapping and the marked alterations in the accessibility of genes during tumourigenesis (Figure S16).

Next, we proceeded to joint profiling of chromatin accessibility and gene expression in individual cells based on the concordance. SCENIC+^113^ analysis was performed to examine the complex relationship between the expression and chromatin dynamics of candidate cis‐regulatory elements (sCREs) and to decipher enhancer‐driven gene regulatory networks (eGRNs) (Figure S15F). We identified 122 high‐quality activator TFs across diverse cell types. sSCENIC+ recovered well‐known master regulators of T cells (Lef1 and Tcf7), B cells (Pax5), erythroid cells (Gata1 and Lmo2), myeloid cells (Irf5, Mafb and Spi1), neural cells (Pou3f1 and Sox10), and stromal cells (Ar, Hic1, Nfia and Nfic). The specific enhancer‐driven regulons of malignant cell clusters, such as Ybx1 in T‐cell lymphoma and AP‐1 family genes in sarcoma, were also demonstrated. For T‐cell lymphoma (Proliferating T cell2_Abnormal), SCENIC+ has concurrently identified Myc as well as its transcriptional co‐activator Runx3, which was consistent with a previous study that MYC transcriptionally activates RUNX3 in human T‐cell lymphoma cells.^114^

To further study the T‐cell tumourigenesis, we identified 9 KO‐specific T‐cell lymphoma clusters that shared the chromatin peaks at tumourigenesis signature loci defined in the scRNA‐seq atlas, including C1‐C5 with extensive chromatin accessibility at the *Myc* and *Pvt1* loci and C6‐C9 with chromatin accessibility primarily at the *Icos* and *Gzma* loci (Figure S16A and B). Notably, these results were supported by label transfer analysis, which associated the scATAC‐seq T‐cell subclusters with T‐cell subtypes in the scRNA‐seq atlas (Figure S16G). To study the chromatin remodelling of tumourigenesis, we performed pseudotime analysis using ArchR along the regulatory trajectory of chromatin accessibility from normal to neoplastic states. Based on accessibility‐based gene activity scores, the two distinct peripheral T tumours showed continuous developmental trajectories (Figure S17A–F). By tracing cells, identifying peak‐to‐gene links, and integrating expression signals from scRNA‐seq data, we denoted the continuous dynamics of regulatory factors along the T‐cell transition trajectory from normal to different neoplastic stages. These two types of non‐thymic T‐cell tumours exhibited similar initiation and development characteristics. The epigenetic alterations and RNA expression profiling demonstrated concordance, evidenced by the early elevation of NF‐κB pathway‐related genes (like *Nfkb1* and *Nfkb2*) and interleukin‐associated genes (like *Irf2*, *Irf4* and *Irf7*), alongside the subsequent augmentation in accessibility of genes such as *Gzma*, *Myc*, *Pvt1* and *Runx2* (Figure S17G–H). Moreover, employing motif‐related analyses, we discerned a cascade of pseudo‐temporally regulated transcription factors throughout the progression of lymphoma. Notably, inflammation‐associated factors such as Nfkb1 and Bcl11b^115^ showed activity in the initial stages, whereas Myc, Runx3 and Rxrb predominated in the tumourigenic phase (Figure S17G–H). The integrative analysis reaffirmed the origin of two non‐thymic T‐cell lymphomas and the emergence of peripheral inflammation. In contrast, in the sarcomagenesis process arising from fibroblast and endothelial cells, remarkable changes were observed in the accessibility of genes linked with AP‐1 transcription complexes (like *Jun* and *Fos*) and Wnt pathway (like *Wnt2*), alongside fluctuations in the enrichment levels of these TF‐corresponding motifs (Figure S17I–M). These observations suggested that the acquisition and loss of chromatin accessibility and TF activity in relevant regions contributed to the gene expression modulation during the progression of the tumours.

Our whole‐genome sequencing (WGS) analysis revealed a gradual increase in the number of single nucleotide variants (SNVs) – escalating from normal to Pre stage, and peaking in tumour samples among different tissues (Figure S18A). We also found that the adjacent genes linked to SNVs within promoter regulatory regions were predominantly associated with intracellular metabolism, ribosome biogenesis, and other fundamental cellular processes (Figure S18B–D). To further explore the clonal changes in regulatory elements, we integrated the DNA sequence information from scATAC‐seq and WGS data (Figure S18E and F). We mapped 7194 SNVs with high confidence from WGS data in noncoding regions to single cells. We confirmed that the proportion of detected mutant cells within each lineage progressively increases with the advancement of tumours in mice (Figure S18E), demonstrating an enhancement in genomic instability induced by p53 inactivation. Specifically, in malignant T cells, we identified the most abundant mutations within or adjacent to the promoter regions of RPGs, indicating that the robust RPG expression in T‐cell lymphoma may be closely related to mutation‐driven regulation. To identify the role of SNVs in the binding of TFs to CREs, we searched for overrepresented DNA sequence motifs in the set of alleles and corresponding reference peak regions (Figure S18F). There were 163 SNVs in the peak regions that helped to obtain the structure of TF motifs, such as the Hoxd11, Irf1, Sox4, Runx2 and Cdx1 motifs. However, 147 SNVs in the peak regions destroyed the structure of TF motifs, such as the Otx1, Gata6, Foxi3, Tbx2 and Bcl11a motifs. It is noteworthy that the activity changes of these transcription factors were also reflected in the aforementioned pseudotemporal analysis of tumourigenesis (Figures S17G and H and S17K). In conclusion, our results supported that SNVs in noncoding regions may play an important role in the cis‐regulatory network.

We identified diverse lineage‐specific differentially accessible chromatin regions between KO and WT cells, indicating significant alterations in the functional elements of the genome (Figure S19A). We found that for certain genes with significant lineage‐specific expression changes, there were consistent alterations in the accessibility of potential regulatory modules, demonstrating the influence of epigenetic factors within the p53 regulatory network. Notably, in contrast to the pronounced expression changes of specific RPGs observed in scRNA‐seq data, the differentially accessible peaks were not enriched in the regulatory regions of RPGs. To explore the epigenetic alterations of RPGs induced by p53 deficiency, we conducted a comprehensive evaluation of the accessibility scores of RPGs across various developmental stages in diverse lineages. As a comparative benchmark, we intentionally included lineage‐specific driver genes as the positive control (PC) and genes silenced in adult mice as the negative control (NC). Our findings suggested that the accessibility of RPGs remained largely stable across all stages, consistently exhibiting high accessibility levels (Figure S19B). The visualisation of scATAC‐seq tracks for RPGs also revealed conspicuous accessible peaks in promoter regions, regardless of the developmental stage (Figure S19C). These findings implied pronounced accessibility of RPGs to regulatory factors, indicating the intricacies inherent in the regulatory programs of RPGs.

### 3.6 Common and specific alterations upon *Trp53* perturbation across diverse cell types

To further investigate whether the variations at the early stage could be extended to other cell types not typically associated with captured tumour profiles, we extracted the data from WT and KO‐pre‐neoplastic groups for comparative analysis. We first assessed the transcriptional heterogeneity of 20 tissues obtained from WT and KO‐pre‐neoplastic mouse cohorts, using the Seurat independent clustering methodology.^43^ For most cell types identified in WT and p53‐deficient tissue‐wide samples, there was considerable overlap between samples and a comparable proportion of cell types. Additionally, we excluded cell types with inadequate cell counts or intercellular cross‐contamination.

**FIGURE 5:**
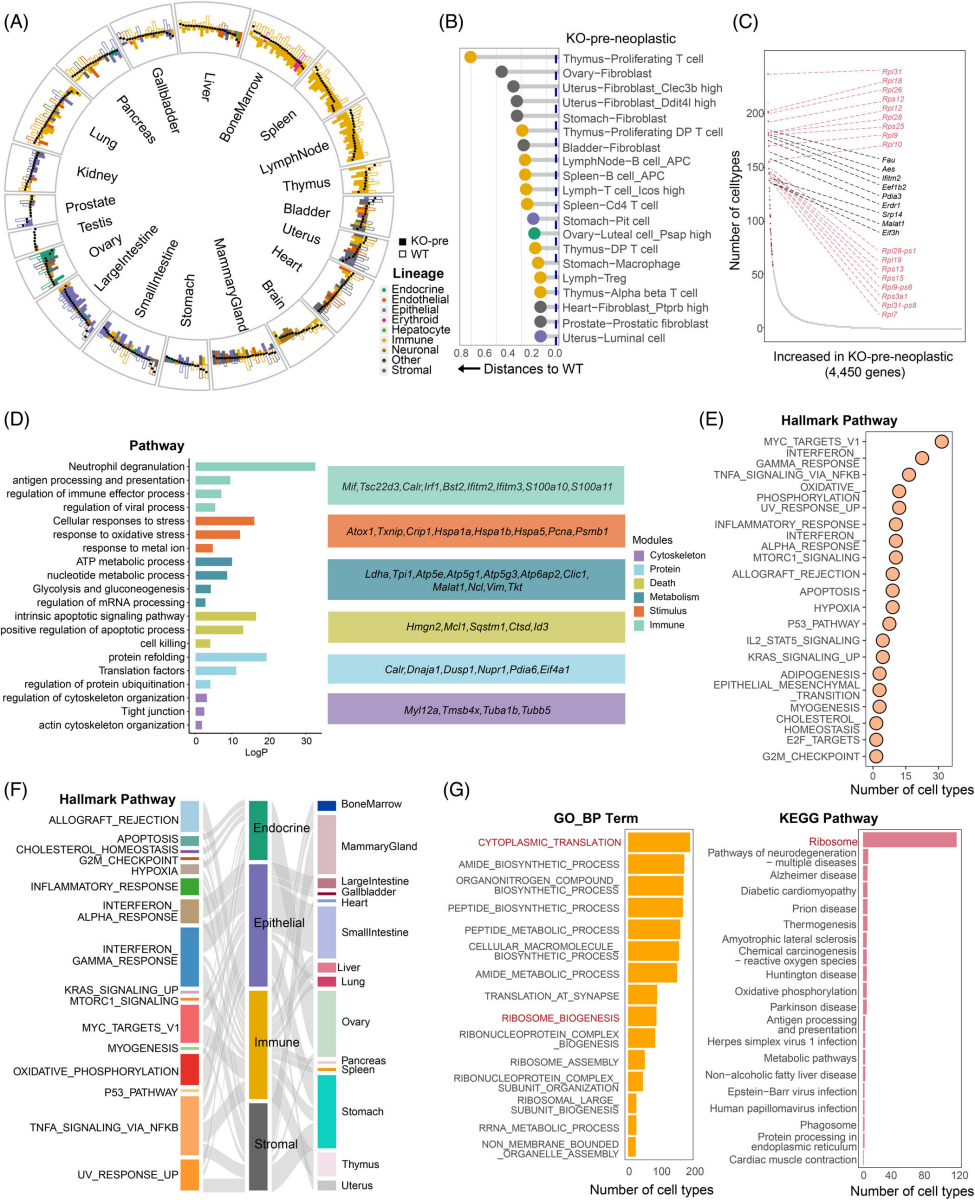
Comparison between WT and KO‐pre‐neoplastic samples. (**A**) Differential abundance plot of the changes for each cell type between WT and KO‐pre‐neoplastic samples with Milo. (**B**) A lollipop plot showing the differences in cell types between WT and KO‐pre‐neoplastic samples, measured by cosine distance. (**C**) Dot plot showing the top commonly upregulated genes (URGs) in KO‐pre‐neoplastic samples relative to WT samples. Red nodes highlight the top common upregulated 46 ribosomal protein genes (RPGs) across more than 50 cell types at KO‐pre‐neoplastic stage (top RPGs). (**D**) Bar chart showing representative signal pathways (adjusted *p* value < .01) enriched in commonly upregulated genes (observed in more than 50 cell types) at KO‐pre‐neoplastic stage. Right panel showing the marker genes in different functional modules. (**E**) Dot plot showing enriched hallmark pathways (adjusted *p* value < .01) for KO‐induced URGs. The *x*‐axis indicates the number of cell types corresponding to each pathway. (**F**) Sankey diagram showing the relationships among hallmark pathways, cell lineages, and tissues. (**G**) Bar chart showing representative GO terms (left) and KEGG pathway (right) enriched in URGs at KO‐pre‐neoplastic stage. The *x*‐axis indicates the number of cell types corresponding to the certain enriched terms or pathways. WT, wild‐type; KO, knockout.

To quantify the magnitude of alterations in cellular composition across diverse cell types upon p53 inactivation, we performed differential abundance analysis on cell neighbourhoods using Milo^45^ (Figures 5A and S20A) and calculated cosine distance between KO‐pre‐neoplastic and WT cells (Figures 5B and S20B). We found that the absence of p53 triggered a noticeable shift in the abundance of nearly all cell types across immune, stromal, epithelial, endothelial, neural, and endocrine cells. This analytical outcome substantiated the extensive and discernible transcriptomic alterations in KO samples during the transitional phase preceding tumourigenesis. Particularly, a drastic variation in immune cell alteration could be noted in immune organs (bone marrow, spleen, lymph nodes, and thymus) and some parenchymal organs, such as the liver, pancreas, lung, and kidney. T/B‐cells as well as macrophages with highly expressed RPGs were enriched in p53‐deficient samples. For the gastrointestinal system, the changes in the small intestine, large intestine, stomach, and mammary gland were mostly attributed to the epithelium with high RPG or Mt1/2 expression^116^ (Figure S20C). In brain, there was a significant increase in immune cells and a decrease in neural cells in p53‐deficient samples. Of note, the high expression of *Trem2*, *Ly86*, *Tgfb1*, and *Fcgr1* highlighted the possible role of KO microglial cells in brain inflammatory and neurodegenerative disorders^117^, ^118^, ^119^, ^120^ (Figure S20D). In addition, multiple cell types including epithelial, stromal, and endothelial cells accounted for the main components of variability in bladder, uterus, mammary gland and heart. In the uterus, upregulated genes *Cxcl17*, *Prap1*, *Arg1* and *Fxyd4* related to pregnancy and embryo implantation were accumulated in KO luminal cells (Figure S20E). These alterations underscore the functional contributions of p53 to non‐neoplastic physiological processes. Overall, while nearly all cell types from the major tissues exhibit molecular phenotypes upon *Trp53* loss, the most pronounced disparities were observed in lymphoid cells, followed by stromal and epithelial cells (Figure 5B). The lineage hierarchy revealed in these results closely aligns with the tumour spectrum observed in p53‐null mice, suggesting that the magnitude of transcriptional changes can partially reflect tumour susceptibility under p53 inactivation.

To investigate the gene sets contributing to the variation in response to p53 loss, we identified 5663 downregulated genes (DRGs) and 4450 upregulated genes (URGs) in p53‐deficient samples covering all cell types from 20 tissues (Wilcox rank‐sum test, *p* < .01 and avg.log_2_ (fold change) > 0.5) (Table S13). Among the top 50 URGs with the highest frequency, RPGs accounted for approximately 58% (29 out of 50), while there were also some genes related to transcription and translation, such as *Eif3h* and *Eef1b2* (Figure 5C). Beyond RPGs, commonly upregulated genes (observed in more than 50 cell types) were enriched in diverse biological processes, including immune response, metabolism, stress response, protein stability, cell death, and cytoskeleton organisation (Figure 5D). A subset of these genes, such as *Ldha*, *Mif*, and *Atox1*, corresponded to the aforementioned tumour heterogeneity hallmark genes, representing recurrent tumourigenic states induced by p53 loss across multiple cell types. Notably, hallmark pathway enrichment analyses aligned closely with observed shifts in cellular state abundance. URGs from cell types with significant changes in abundance and cosine distance mapped to Molecular Signatures Database (MSigDB) hallmark gene sets, including Myc targets, oxidative phosphorylation, apoptosis, hypoxia, inflammatory response, and other immune‐related pathways, which exhibited cell lineage and tissue specificity (Figure 5E and F). Collectively, these findings demonstrate that cellular sensitivity to *Trp53* loss is dynamically modulated across cell types and tissues, yet retains critical conserved features.

Remarkably, based on the cellular GO enrichment analysis at cell‐type level, ribosomal pathways were prevalent across various cell types (Figure 5G). The ribosomal protein synthesis is paramount in cellular activities, and dysregulation in ribosomal genes inevitably disrupts cellular homeostasis. To assess whether p53‐deficiency causes high levels of RPGs by affecting proliferation, we examined the proliferation rate and proportion of proliferating cells (G2/M/S phase) in the thymus, spleen, and lymph nodes of KO and WT mice using the 5‐ethynyl‐2′‐deoxyuridine (EdU) proliferation and propidium iodide (PI) staining assay (Figure S21). Correspondingly, relevant cell‐cycle indicators were computed based on transcriptomic data utilising Seurat (Figure S22). Both the experiment and bioinformatics analysis demonstrated that there was no significant difference in the cell proliferation between KO‐pre‐neoplastic and WT tissues, which were markedly different from the highly proliferating tumour or metastasis samples. It has been reported that there is also no considerable alteration in the apoptosis of thymic p53‐deficient cells.^121^ These findings indicated that p53 inactivation triggered variations in cellular abundance and gene expression prior to tumourigenesis, without affecting the progression of cell proliferation and apoptosis.

To systematically evaluate the KO effect, we identified 46 RPGs that exhibited upregulation across more than 50 cell types (top RPGs) and computed the collective expression score of these 46 RPGs using AUCell^49^ method as a representative response feature induced by p53 KO. The enhancement in RPG scores was demonstrated across diverse cell lineages (Figure S23A), devoid of discernible batch influence attributable to the origins of individual mice (Figure S23B). Our analysis also revealed a higher enrichment of these top RPGs in progenitor cells compared to those cells nearing the differentiation terminus within our dataset (Figure S23C). Concordantly, the increase in entropy values and ESC‐like module scores of p53‐deficient cells was observed (Figure S24), signifying that the elevation of the specific RPG expression represents the intensification of cellular differentiation potential upon p53 inactivation. Notably, for the aforementioned tumour‐prone clusters (Figure 3) whether derived from WT or KO samples, these cells demonstrate a notable augmentation in the RPG scores and increased potential for differentiation (Figure S25A), suggesting that the expression levels of specific RPGs could serve as quantitative indicators of tumour susceptibility for various lineages under p53 inactivation. KO tumour‐prone cells also displayed enhanced activity in glycolysis, the TCA cycle, and macromolecule synthesis pathways (lipids, amino acids, and glycans), alongside mTOR pathway activation (Figure S25B and C). These observations suggest that p53 loss of function promotes irreversible cross‐lineage tumourigenic progression by driving sustained self‐renewal, bolstered energy supply, anabolic metabolism, and oncogenic pathway activation.

To explore whether the variations in the expression pattern of RPGs due to p53 deficiency are also present in human cancers, we conducted further analysis using single‐cell transcriptomic data obtained from human cancer tissues with and without *TP53* mutations^122^ (Figure S26 and Table S10). Remarkably, samples harbouring *TP53* deleterious mutations exhibited notably elevated expression levels of RPGs and enrichment of ribosomal pathways across different cancer types. Besides, considerable overlap was observed between this subset of dysregulated RPGs and those specifically identified in the genesis of KO mouse tumour‐prone T cells. Furthermore, we observed that the transcription level of p53 appears to be negatively correlated with the transcription level of these specific RPGs. Although the sample size is insufficient, these observations provided preliminary evidence that the influence of p53 inactivation on multi‐lineage tumourigenesis might partially stem from disruptions in RPG regulation.

### 3.7 Deciphering the regulatory architecture of *Trp53*

**FIGURE 6:**
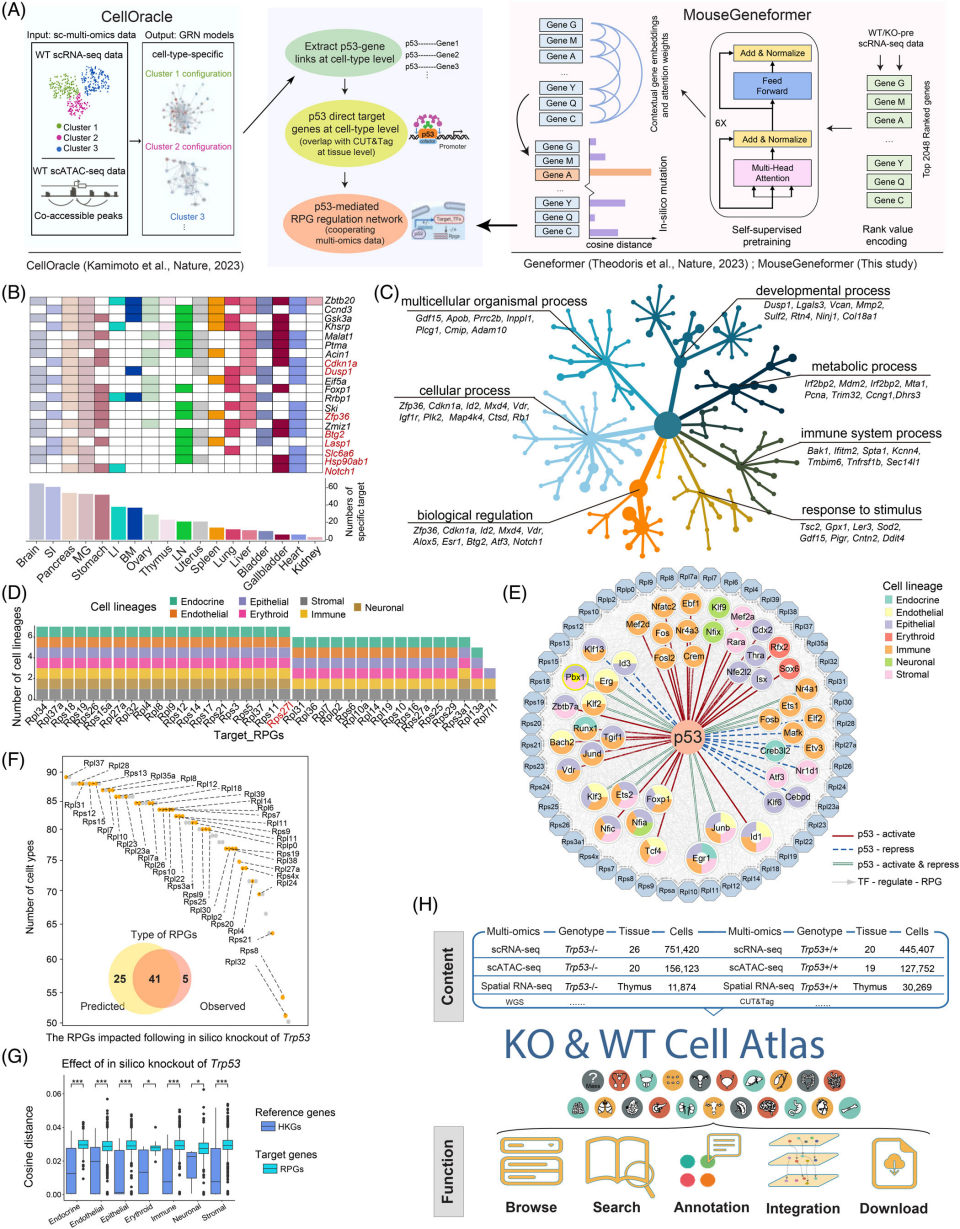
Systematic dissection of p53 regulatory program. (**A**) Overview of the analysis workflow. First, transcriptional gene regulatory networks (GRNs) were constructed by integrating scRNA‐seq and scATAC‐seq data using CellOracle (left). P53 direct targets were identified by CUT&Tag verification and putative p53 direct target genes with the capacity to regulate ribosomal protein genes (RPGs) were identified via multi‐omics analysis at the cell‐type level (middle). Finally, simulated knockout experiments were conducted using an extension model of GeneFormer, MouseFormer (right), to validate the p53‐mediated RPG regulatory network. (**B**) Grid plot showing the distribution of common p53 functional targets across various lineages (top), and bar plot showing the numbers of lineage‐specific p53 functional targets (bottom). (**C**) Tree visualisation of the Gene Ontology (GO) terms enriched among 2473 p53 target genes. The central node represents certain biological processes and the branch represents the level of the GO hierarchy. Seven major biological processes and representative target genes are labelled. (**D**) Bar plot showing the prevalence of lineage‐common p53 target RPGs. (**E**) Network illustrating the regulation of the top RPGs indirectly regulated by p53. Pie charts illustrate the cell lineage heterogeneity of transcription factor (TF) regulation. Blue dashed lines signify negative regulation, and red solid lines imply positive regulation, while green double lines indicate the coexistence of both positive and negative regulation from p53 to TFs. The grey arrows indicate the TF regulation upon RPGs. (**F**) Dot plot showing the RPGs most commonly affected in the in silico knockout of *Trp53* (across more than 50 cell types). Orange dots indicate that these RPGs can also be observed in the actual knockout of *Trp53*. Venn diagram showing the overlap of the most frequently affected RPGs in the in silico knockout and those experimentally observed to be upregulated (top upregulated RPGs across more than 50 cell types, Figure 5C). (**G**) Boxplot showing the effect of in silico knockout of p53 on top RPGs (upregulated top RPGs across more than 50 cell types in Figure 5C) and housekeeping genes in different cell lineages, measured by cosine distance. (**H**) Overview of the KO&WT cell atlas website construction. WT, wild‐type; KO, knockout.

To gain deeper insights into the modifications within the underlying gene regulatory framework following *Trp53* depletion, we employed machine learning methodologies to decode the complex single‐cell datasets and extract refined information (Figure 6A). Firstly, we utilised the CellOracle^66^ tool to elucidate the cell‐type‐specific p53 regulatory network across diverse tissues and lineages of wild‐type (WT) mice, leveraging the integration of scRNA‐seq and scATAC‐seq data. Confronted with an expansive regulatory network, our objective was to discern the presumptive direct functional targets of basally expressed p53 at the cell‐type level, facilitating the interpretation of the initial layer of regulation. We conducted CUT&Tag^123^ analysis on WT‐p53 across 58 samples from 18 tissues and selected direct target genes supported by at least 2 CUT&Tag samples at promoter regions (Table S8). To eliminate the false positive results, we intersected the putative targets from CellOracle and CUT&Tag in the corresponding tissues, and identified 2473 genes as highly confident direct target genes at the cell‐type level (Table S14).

Our research revealed that p53 targets were widely distributed in various tissues, encompassing well‐established pivotal targets of p53^124^ such as *Cdkn1a*, *Dusp1*, *Zfp36*, *Btg2*, *Lasp1*, *Slc6a6*, *Hsp90ab1*, and *Notch1* as well as numerous tissue‐specific targets of p53 (Figure 6B). The heat‐tree built with metacoder^125^ showed that p53 direct targets were enriched in the following categories of functions: metabolic process, multicellular organismal process, response to stimulus, cellular process, immune system, biological regulation, and developmental process (Figure 6C). This underscored the central regulatory role of the p53 and their potential contribution to the complexity and diversity of tissue functions. Furthermore, among the tumour hallmark genes with altered expression upon p53 inactivation, a subset comprised p53 target genes, indicating direct perturbations to p53‐mediated regulatory networks (Figure S27A). Lineage‐specific p53 targets manifested pathway enrichment profiles consistent with their lineage functions such as cognition of neural cells, immune response of immune cells and ossification of stromal cells (Figure S27B). These findings provided insight into the heterogeneity observed in p53's homeostatic regulations.

Additionally, there was a predominant enrichment of RPGs among lineage‐common target genes, notably including the well‐known target gene *Rps27l* ^126^ (Figure 6D). This finding pointed to the possibility of direct regulation of a subset of RPGs by p53. However, not all upregulated RPGs following p53 deletion were under its direct target, indicating that p53's indirect regulatory mechanisms significantly impacted RPGs as well. By integrating scRNA‐seq data (differential expression and SCENIC analysis), and scATAC‐seq data (motif enrichment in peaks, and Co‐accessibility analysis), coupled with motif scanning in the RPG promoter regions, we discerned crucial TFs among the p53 direct targets that are likely to exert regulatory influence on RPGs (Figure 6E). Moreover, based on the TF‐target gene interactions databases, there is indeed an enrichment of RPGs among the known targets of these selected TFs.^127^ Consequently, a regulatory network at the second layer was delineated.

Given the complexities and challenges of experimentally validating these intermediates, we employed computational model Geneformer to confirm the putative TF‐RPG relationships. Based on WT and pre‐neoplastic scRNA‐seq data, we developed a model designated as MouseFormer to elucidate intrinsic transcriptional regulatory relationships between TFs and genes at single‐cell resolution. By visualising the dimensionality‐reduced cell embeddings learned by MouseFormer, we were able to discern that the model had effectively captured the underlying tissue‐level information (Figure S28A and B). Besides, in silico deletion of *Trp53* triggered pronounced changes in the embeddings of RPGs and other up‐/downregulated genes at Pre state, showing a substantial agreement with scRNA‐seq findings (Figures 6F and S28C–E). These results indicated the strong predictive capacity of our model. Subsequently, we performed the same KO program on candidate regulatory TFs in corresponding Pre and WT lineages to verify the regulatory programs. In silico deletion of p53‐positive‐regulated TFs should have a more deleterious effect on RPG embeddings under the WT condition compared to the Pre state; vice versa for p53‐negative‐regulated TFs. Following this rule, we identified a series of p53‐positive/negative‐regulated TFs that met the criteria, such as *Junb*, *Egr1*, *Klf3*, *Ets2*, *Foxp1*, *Nfic* and *Jund* (Figures 6G and S28F).

In summary, our multi‐omics analysis, coupled with experimental validation, provides comprehensive insights into the multifaceted regulatory roles of p53 in controlling the expression of downstream genes with lineage heterogeneity. We have constructed an integrated online portal (Figure 6H), the Atlas resources (http://bis.zju.edu.cn/KO_Atlas/Trp53/), which provides a foundation for a large‐scale multi‐omics single‐cell atlas and serves as a powerful tool for exploring the potential molecular pathways with biological importance.

## 4 DISCUSSION

Previous p53‐related multi‐omics studies have largely been confined to specific anatomical sites or bulk tissue levels, limiting their ability to reveal the impact of p53 deficiency across diverse tissues and cell types, as well as the process of tumourigenesis. Here, we have constructed the first comprehensive KO cell landscape at the organism scale and provided an ideal resource for exploring how the loss of a tumour suppressor gene orchestrates cellular changes across tissues culminating in the emergence of multi‐lineage tumours. Our study design enabled systematic distinction between lineage‐conserved and lineage‐specific effects of p53 inactivation through an integrated cross‐lineage comparative framework. By simultaneously analysing T cells, B cells, fibroblasts, and epithelial cells under standardised p53‐deficient conditions, we identified ribosomal protein gene (RPG) upregulation as a universal early event across all lineages – a conserved molecular response preceding malignant transformation. This pan‐lineage signature was accompanied by consistent disruption of cellular homeostasis pathways. Crucially, however, this shared stress response manifested in lineage‐distinct oncogenic trajectories: T cells exhibited thymic developmental arrest through Notch1 activation and Rag1/2 suppression, culminating in spatially‐confirmed lymphoma subtypes; B cells demonstrated pre‐B receptor hyperactivation with unique antigen‐presentation alterations; fibroblasts developed sarcoma via mesenchymal pathway activation (Ddit4/Postn overexpression); while epithelial cells underwent tissue‐specific EMT programs. Spatial transcriptomics further validated these divergent paths, showing thymic T‐cell clusters with deterministic chromatin disruption versus microenvironment‐driven adaptations in peripheral tumours. The combinatorial effect of this conserved ribosomal dysregulation with lineage‐instructive molecular contexts – whether developmental programs in lymphocytes, mesenchymal signals in fibroblasts, or epithelial plasticity mechanisms – ultimately orchestrates the multi‐lineage tumourigenesis observed in p53 deficiency. Future studies will clinically anchor our discoveries by: (1) formally assessing tumour‐initiating potential of pre‐malignant cells through transplantation and genetic lineage‐tracing; (2) validating p53‐dependent RPG‐regulating TFs in human datasets to identify therapeutically viable targets for pharmacological testing; (3) leveraging lineage‐specific gene perturbations for synthetic lethal targeting in defined p53‐deficient cancers, with target prioritisation informed by computational evaluation.

Our research has thoroughly updated the understanding of the tumourigenesis in p53 KO mice and provides valuable resources for Li‐Fraumeni syndrome research (Figure S29). We systematically mapped programmed cell fate transitions and TME remodelling involving stress response, proliferative, metabolic, and inflammatory reprogramming. Notably, the oncogenic competence conferred by p53 loss exhibits lineage‐dependent heterogeneity. The intrinsic features of tumour‐prone cells under p53‐deficient conditions, along with their early KO molecular adaptations, are of critical interest, as these transitional states may retain plasticity and reversibility.^128^ Lineage‐specific p53 target networks establish a framework for mapping tumour progression dynamics and synthetic lethal drug screening. Additionally, studies conducted by different laboratories have established a correlation between p53 inactivation and changes in immune response, primarily manifested as inflammation.^129^, ^130^ Besides, changes in metabolic pathways, caused by p53 inactivation, specifically increases in anabolic metabolism and decreases in catabolic metabolism, have been observed to facilitate the activation of immune responses.^103^, ^131^ Our analysis indicated the potential connection among p53 inactivation, immune response, and metabolism. Essential observations included the inflammatory characteristics following the loss of p53, the enhancement of synthetic metabolism represented by the ribosomal pathway, the decrease of catabolism, and the early immune activation of T/NKT tumour initiation. Additionally, at the KO‐Post‐NT state, prolonged immune activation eventually culminated in the accrual of tissue damage and immune exhaustion,^132^ particularly concurrent with the emergence and metastasis of lymphoma. This exerted additional pressure and further diminished the survival niche of normal immune cells, consequently fostering a conducive environment for tumourigenesis in other lineages, such as stroma and epithelium. These findings might help us to arrive at a more conclusive understanding of the pathogenic role of p53 inactivation in exacerbating chronic inflammation and provide new insights for developing p53‐targeted immunotherapies.^133^

Beyond concentrating on the cell lineages with observable phenotypes, our analysis also broadens to include the diverse cell types covering major tissues and underscores the frequently overlooked changes prior to tumourigenesis. Interestingly, our findings demonstrate an increased expression of RPGs across different cell types including immune, stromal, epithelial, endocrine, neural, erythroid, and endothelial cells. While our current human cohort remains limited, these findings offer preliminary evidence that RPG dysregulation represents a conserved early event in p53‐driven tumourigenesis (Figure S26). This aligns with previous studies indicating that the upregulation of ribosome biogenesis isn't simply a passive consequence of tumourigenesis, but instead plays a key role in promoting this process.^134^ In addition, pharmacological inhibition of the mTOR pathway, which suppresses ribosome biogenesis, has been demonstrated to delay tumourigenesis in both *Trp53*–/– and *Trp53*+/– mice.^135^, ^136^ Such findings imply that RPG upregulation is an important early event in p53 loss‐dependent tumourigenesis, and pharmacological compensation of other RPG regulatory pathways may be an effective approach to inhibiting tumourigenesis.

Our study explored the biological significance and molecular mechanism of the upregulated RPG phenotype after p53 inactivation, but several limitations still need to be noted. The dysregulation of RPGs and other ribosomal components has been extensively linked to diverse pathological processes, encompassing cancer, cardiovascular diseases, aging, and neurodegenerative disorders.^137^ Our analysis centres on the overall effect of the upregulation of RPG expression, yet it's important to note that the ribosome‐independent functions of RPGs should also be greatly appreciated.^138^ For instance, ribosome proteins can inactivate the oncoprotein c‐Myc, such as RPL11,^139^ RPS14^140^ and RPL5.^141^ The altered expression of specific RPGs could induce a wide array of phenotypes, ranging from developmental anomalies to pathological conditions.^142^ Additionally, it is necessary to acknowledge that stable production of ribosomal proteins has previously been proposed as essential for the maintenance of proper ribosome biogenesis.^143^, ^144^ Further validation is warranted to ascertain whether the upregulation of certain RPGs results in compromised ribosome biogenesis or the emergence of specialised ribosomes such as ‘onco‐ribosomes’.^145^ Moreover, owing to the common technical constraints in mRNA polyadenylation capture, the expression status of rRNA remains uncertain, which is closely correlated with the pace of ribosome biogenesis.^143^ Evidence from prior studies indicates that p53 potentially plays a role in the repression of rRNA expression.^146^ In summary, our findings suggested the elevated expression of RPGs as a widespread and significant alteration offering oncogenic support to tumours induced by p53 deficiency; however, the intricate functional changes associated with ribosomes mediated by p53 require further exploration.

## AUTHOR CONTRIBUTIONS

X.H., G.G., and J. W. conceived the study. G.G. and X.H. supervised the study. X.H., Xinru W., Xueyi W., R.W., D.Z., X.F., L.Y., H.N., H.D., and Y.L. performed all the experiments. Y.M., J.W., Xinru W., H.W., P.Z., J.L., L.M., W.E., and Z.S. performed all computational analyses. Y.Z., and M.C. helped with website construction. M.J, D.J., and T.Z. helped with high‐throughput sequencing. Q.Z. and T.L. provided the human samples. H.H., H.O., and J.P. helped with experiment scheme. G.G., Xinru W., J. W., and Y.M. wrote the initial draft of the manuscript. All authors participated in discussions of results and manuscript editing.

## CONFLICT OF INTEREST STATEMENT

The authors declare that they have no competing interests.

## ETHICS STATEMENT

All mouse experiments performed in this study were approved by the Animal Ethics Committee of Zhejiang University. All mouse experiments conformed to the relevant regulatory standards at Zhejiang University Laboratory Animal Center. All human patients gave their written informed consent for scientific evaluations. The human study was approved by the Ethics Committee of The First Affiliated Hospital and The Second Affiliated Hospital, Zhejiang University School of Medicine.

## Supporting information



Supporting Information

Supporting Information

Supporting Information

Supporting Information

Supporting Information

Supporting Information

Supporting Information

Supporting Information

Supporting Information

Supporting Information

Supporting Information

Supporting Information

Supporting Information

Supporting Information

Supporting Information

## Data Availability

Raw data files for the mouse scRNA, scATAC, and CUT&Tag sequencing analysis reported in this paper have been deposited in the NCBI Gene Expression Omnibus under accession number GSE217664 (https://www.ncbi.nlm.nih.gov/gsssseo/query/acc.cgi?acc= GSE217664/, enter token: klwfmkogjfqbful). Files with expression data/chromatin accessible data, clustering, and annotation can be downloaded for each tissue and all data combined from https://figshare.com/s/6f4ab181391a0c747bb6. WT data can be accessed at http://bis.zju.edu.cn/MCA/, and KO data can be accessed at http://bis.zju.edu.cn/KO_Atlas/Trp53/. The raw human sequence data reported in this paper have been deposited in the Genome Sequence Archive in National Genomics Data Center, China National Center for Bioinformation/Beijing Institute of Genomics, Chinese Academy of Sciences (GSA‐Human: HRA006591; HRA004497) that are publicly accessible at https://ngdc.cncb.ac.cn/gsa‐human.The source code for reproducing our analysis and running and training the MouseFormer models is available at GitHub (https://github.com/ggjlab/MouseFormer). Custom codes and scripts used for analysis could be accessed at https://github.com/ggjlab/P53‐Analysis_pipeline/.
