# Supplementary material for: Dissecting cross‐lineage tumourigenesis under p53 inactivation through single‐cell multi‐omics and spatial transcriptomics
Source: Clin Transl Med. 2025 Aug 31;15(9):e70461. doi: 10.1002/ctm2.70461 (PMC12399789; doi:10.1002/ctm2.70461)
Supplement: Supplementary file 1 — Supporting Information [file CTM2-15-e70461-s006.pdf]

Figure S1

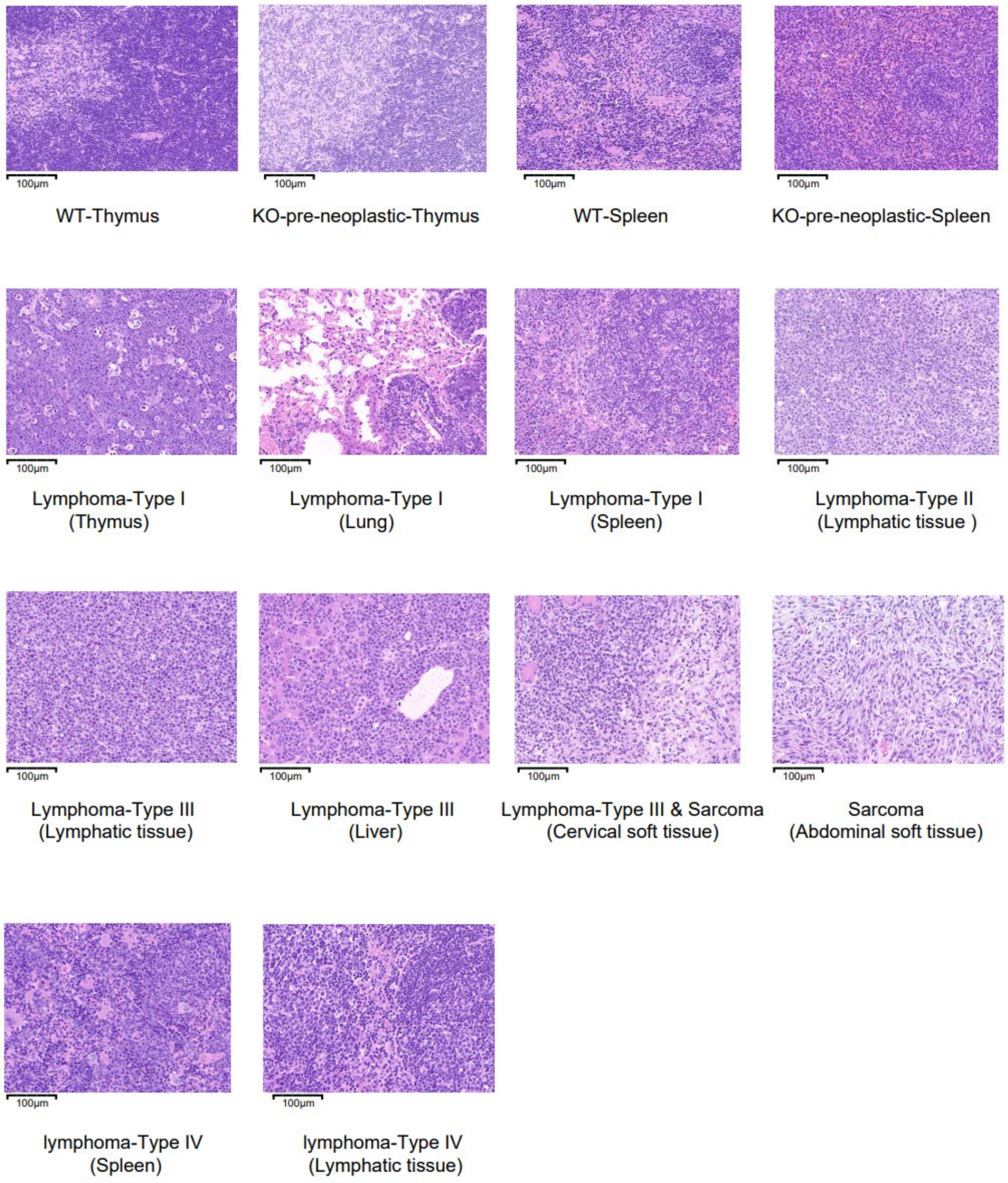


**Fig. S1. The histopathology of representative KO samples and the corresponding histophysiology of WT samples.** Four morphologically distinct lymphomas, sarcomas, mixed tumors, and pre-neoplastic stage samples with no apparent abnormalities were observed. WT, wild-type; KO, knockout.

Figure S2

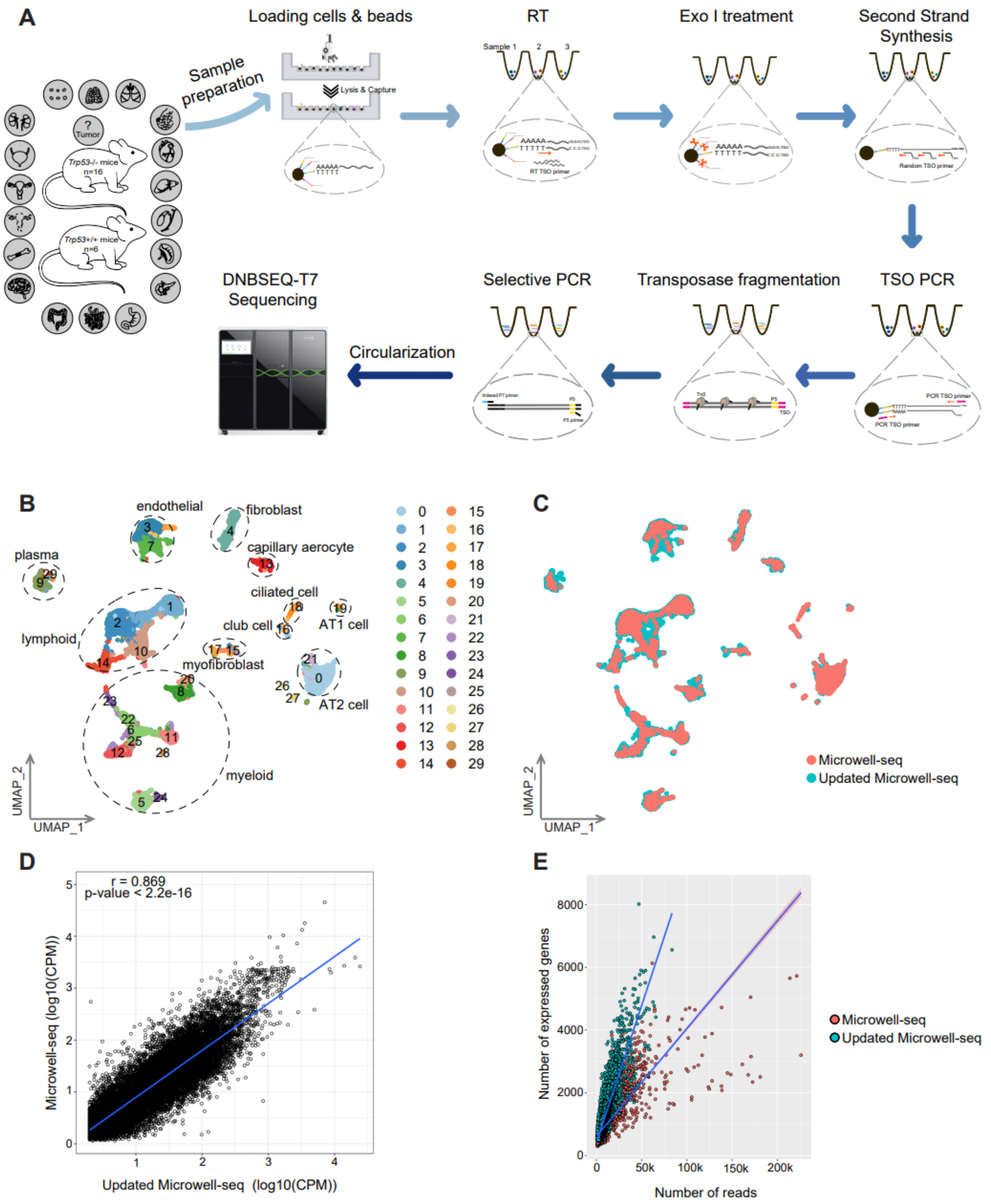


**Fig. S2. Schematic of updated Microwell-seq workflow and library quality of the platform.**

(**A**) The workflow of updated Microwell-seq. After the preparation of single-cell suspensions of fresh tissues from *Trp53*$^{-/-}$ and wild-type mice, cells and barcoded beads were loaded into microwells. For the next steps, compared to Microwell-seq, second-strand synthesis was introduced in updated Microwell-seq to improve the efficiency of

cDNA amplification, and DNBSEQ-T7 was used to reduce the cost of sequencing. For a more detailed description of key steps, see the supplementary material.

(**B**) UMAP visualization of mouse lung cells, colored by cell clusters and labeled by cell-type annotations.

(**C**) UMAP plot showing the integration of mouse lung cells using the original Microwell-seq platform and updated Microwell-seq platform.

(**D**) Dot plot showing the correlation of UMI counts of expressed genes between the original and updated Microwell-seq platform.

(**E**) Reads to gene plot of the two platforms. The updated Microwell-seq platform shows higher sensitivity and stability to detect more genes.

## Figure S3

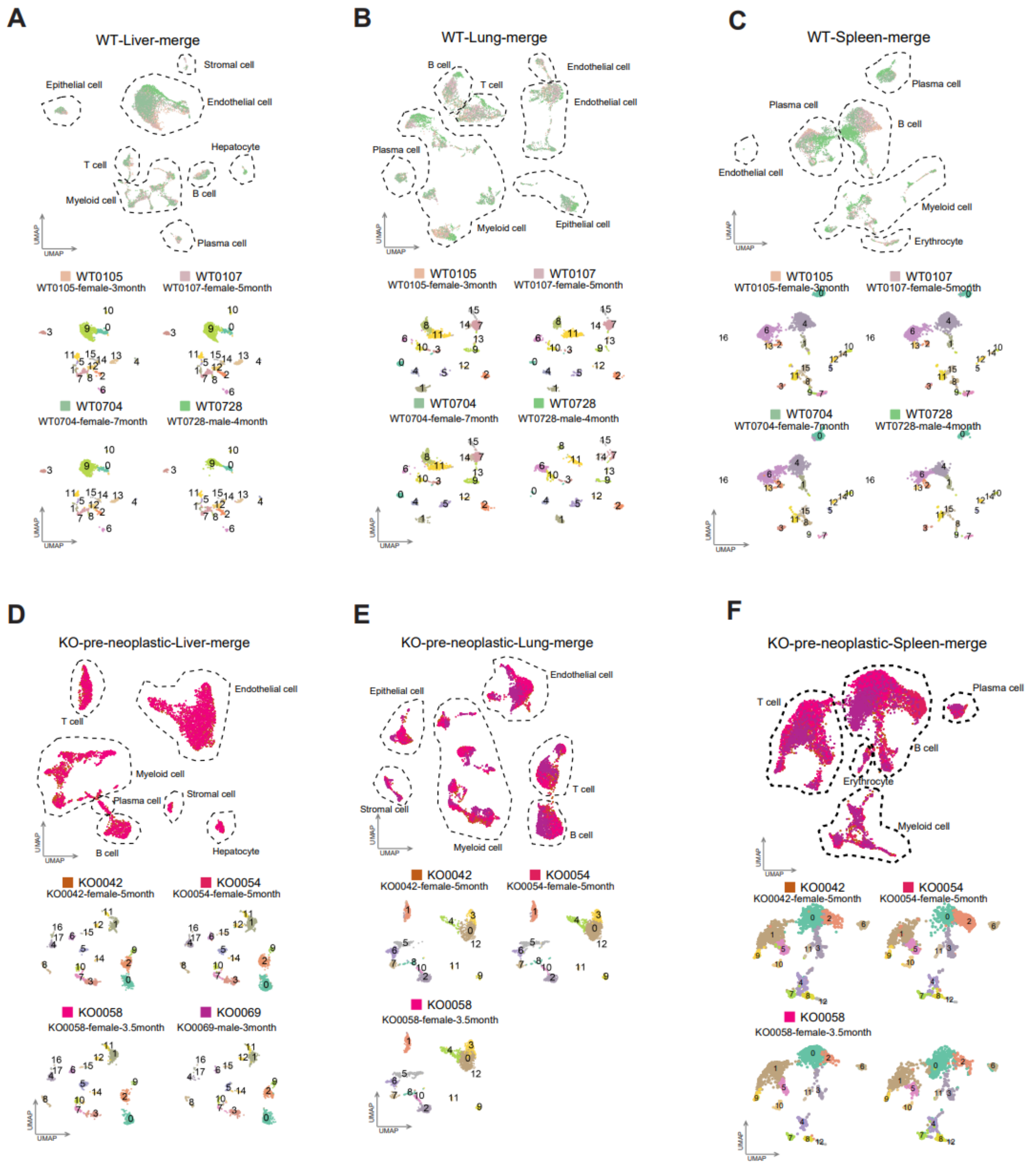


**Fig. S3. Evaluation of batch effects between wild-type and KO-pre-neoplastic mice.** (**A**) UMAP visualization of merged wild-type (WT) cells from liver, colored by cell mouse id (top). UMAP visualization of WT cells from liver split by mouse id, colored by cell clusters (bottom).

(**B**) UMAP visualization of merged WT cells from lung, colored by cell mouse id (top). UMAP visualization of WT cells from lung split by mouse id, colored by cell clusters (bottom).

(**C**) UMAP visualization of merged WT cells from spleen, colored by mouse id (top). UMAP visualization of WT cells from spleen split by cell mouse id, colored by cell

clusters (bottom).

(**D**) UMAP visualization of merged KO-pre-neoplastic cells from liver, colored by cell mouse id (top). UMAP visualization of KO-pre-neoplastic cells from liver split by mouse id, colored by cell clusters (bottom).

(**E**) UMAP visualization of merged KO-pre-neoplastic cells from lung, colored by cell mouse id (top). UMAP visualization of KO-pre-neoplastic cells from lung split by mouse id, colored by cell clusters (bottom).

(**F**) UMAP visualization of merged KO-pre-neoplastic cells from spleen, colored by cell mouse id (top). UMAP visualization of KO-pre-neoplastic cells from spleen split by mouse id, colored by cell clusters (bottom).

## Figure S4

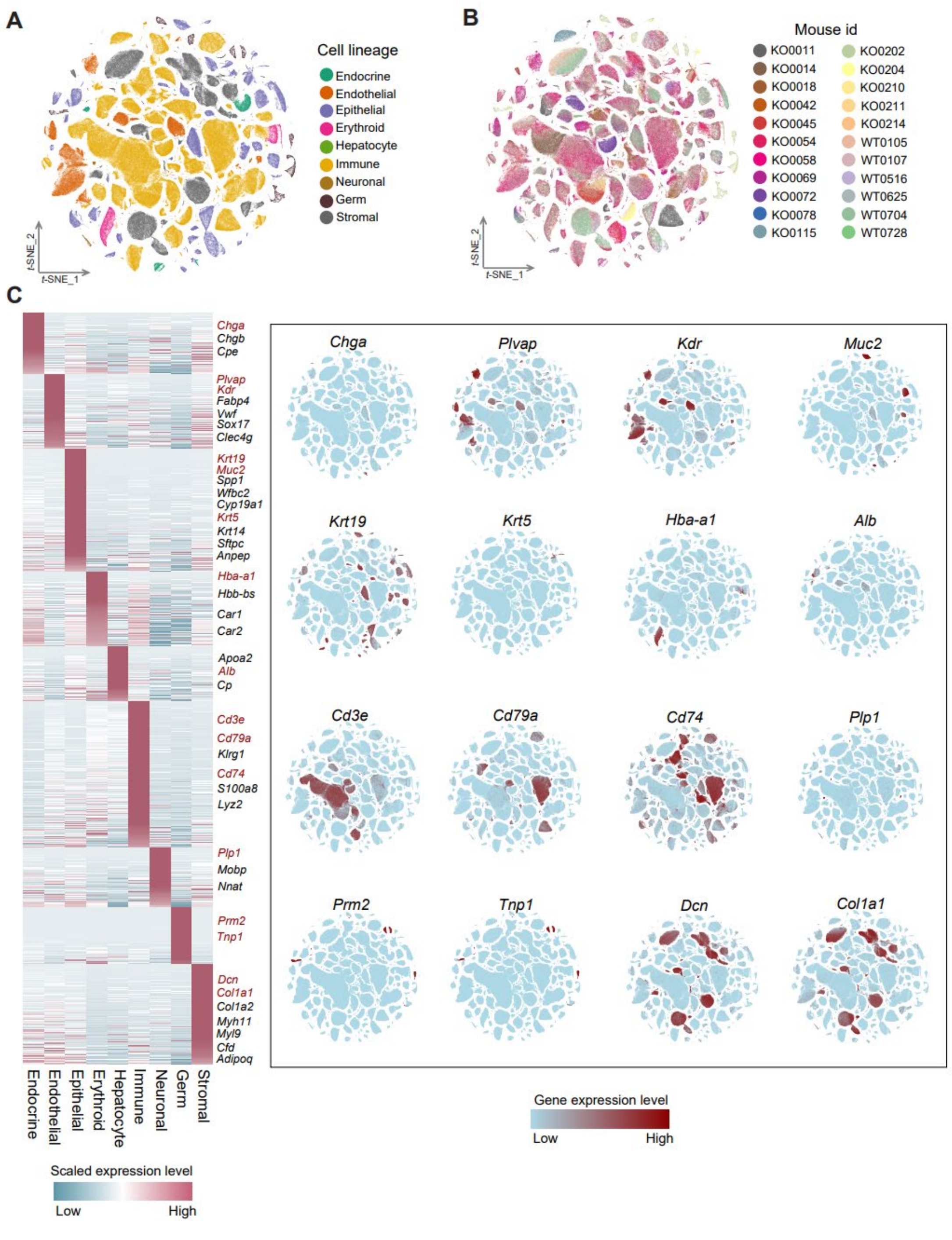


**Fig. S4. Marker genes of different cell types in the *Trp53* functional single-cell atlas.**

(**A**) *t*-SNE visualization of 1,196,827 single cells, colored by cell lineages.

(**B**) *t*-SNE visualization of 1,196,827 single cells, colored by mouse id.

(**C**) Heatmap showing the scaled average expression levels of mouse cell type-specific

marker genes (left), and relative gene expression of representative cell type-specific markers for each cell type overlaid on t-SNE plots (right).

**Figure S5**

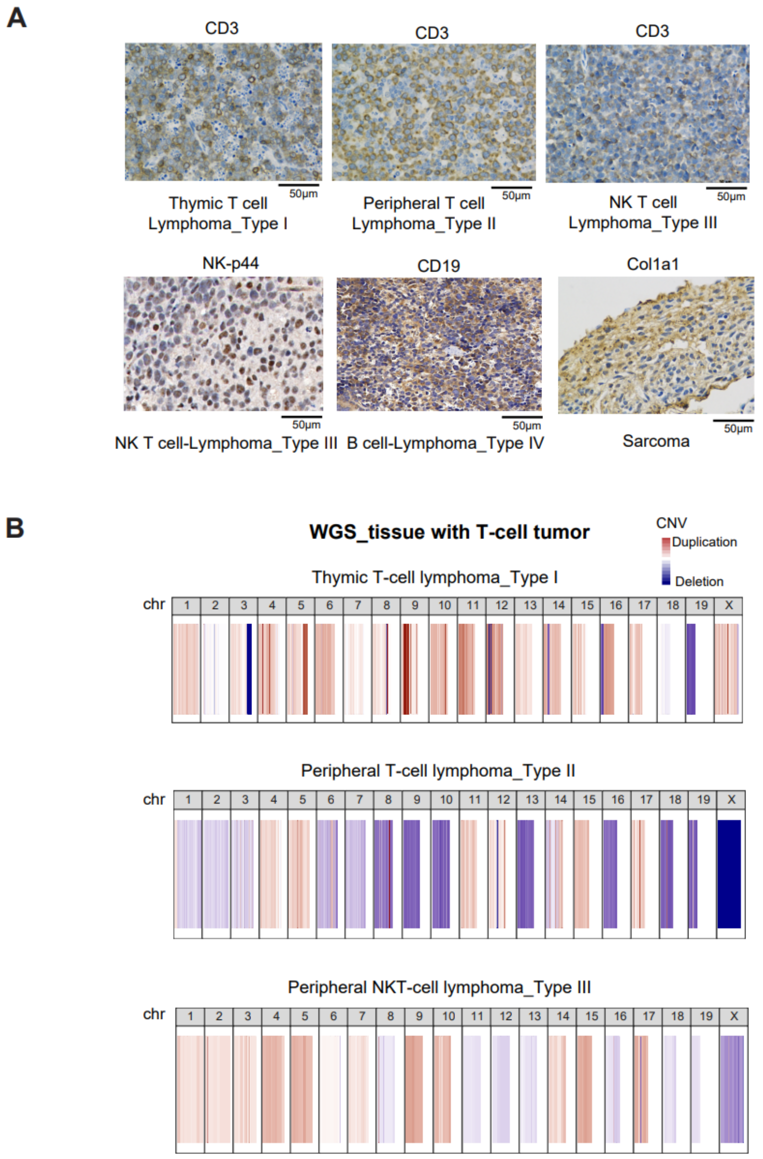


**Fig. S5. The IHC staining and inferCNV analyses of tumor samples.**

(**A**) High immunohistochemical (IHC) staining of the marker proteins of different tumor samples.

(**B**) Heatmap showing the copy number variation (CNV) in different tumor samples inferred from WGS data.

## Figure S6

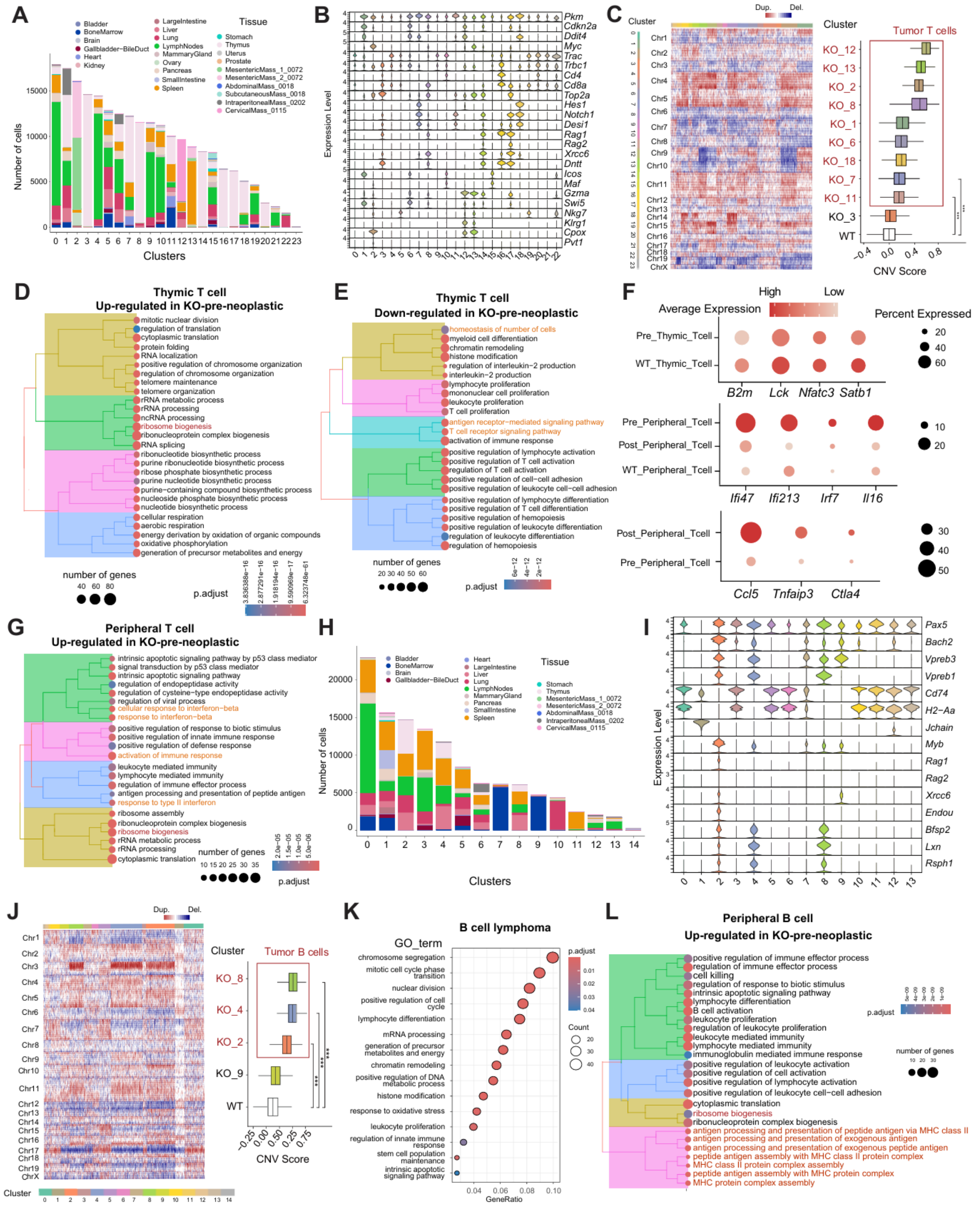

**Fig. S6. Global reclustering of T cells and B cells.**

(**A**) Bar plot showing the number of sequenced cells per T-cell subcluster prepared from different tissues.

(**B**) Violin plots showing representative marker genes of T-cell subtypes.

(**C**) CNV plot of T cells employing inferCNV. Bottom labels indicate different chromosome regions and left color bars indicate different cell subclusters (left). Box

plots showing CNV scores of different T-cell subclusters from WT/KO group (right).

(**D**) Hierarchical clustering tree showing representative GO terms enriched in URGs of KO-pre-neoplastic thymic T cells compared to WT thymic T cells. Dot size denotes the gene count and color denotes the adjusted p value.

(**E**) Hierarchical clustering tree showing representative GO terms enriched in DRGs of KO-pre-neoplastic thymic T cells compared to WT thymic T cells. Dot size denotes the gene count and color denotes the adjusted p value.

(**F**) Dot plot showing the expression of signature genes of thymic or peripheral T cells at different stages. Both color and size indicate the effect size.

(**G**) Hierarchical clustering tree showing representative GO terms enriched in URGs of KO-pre-neoplastic peripheral T cells compared to WT peripheral T cells. Dot size denotes the gene count and color denotes the adjusted p value.

(**H**) Bar plot showing the number of sequenced cells per B-cell subcluster prepared from different tissues.

(**I**) Violin plots showing representative marker genes of B-cell subclusters.

(**J**) CNV plot of B cells employing inferCNV. Bottom labels indicate different chromosome regions and color bars indicate different cell clusters (left). Box plots showing CNV scores of different B-cell subclusters from WT/KO group (right).

(**K**) Dot plot showing representative GO terms enriched in marker genes of the B-lymphoma based on functional enrichment analysis. Dot size denotes the gene count and color denotes the adjusted p value.

(**L**) Hierarchical clustering tree showing representative GO terms enriched in URGs of KO-pre-neoplastic peripheral B cells compared to WT peripheral B cells. Dot size denotes the gene count and color denotes the adjusted p value.

WT, wild-type; KO, knockout.

## Figure S7

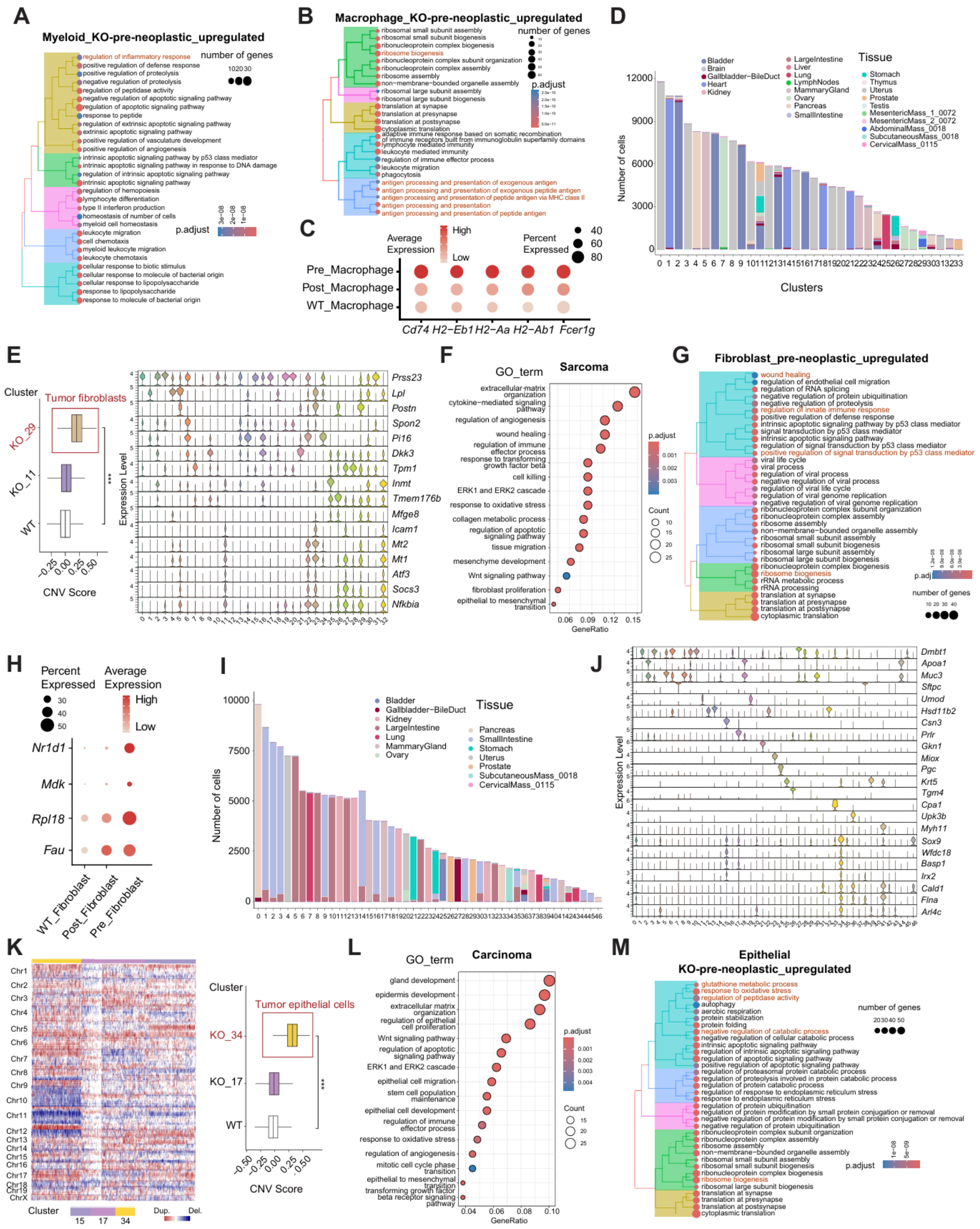

**Fig. S7. Global reclustering of myeloid, fibroblasts and epithelial cells.**

(**A**) Hierarchical clustering tree showing representative Gene Ontology (GO) terms enriched in URGs of KO-pre-neoplastic myeloid cells compared to WT myeloid cells. Dot size denotes the gene count and color denotes the adjusted p value.

(**B**) Hierarchical clustering tree showing representative GO terms enriched in URGs of

KO-pre-neoplastic macrophages compared to WT macrophages. Dot size denotes the gene count and color denotes the adjusted p value.

(**C**) Dot plot showing the expression of signature genes of myeloid cells at different stages. Both color and size indicate the effect size.

(**D**) Bar plot showing the number of sequenced cells per fibroblast subcluster prepared from different tissues.

(**E**) Box plots showing CNV scores of different fibroblast subclusters from WT/KO group (left). Violin plots showing representative marker genes of fibroblast subclusters (right).

(**F**) Dot plot showing representative GO terms enriched in marker genes of the sarcoma based on functional enrichment analysis. Dot size denotes the gene count and color denotes the adjusted p value.

(**G**) Hierarchical clustering tree showing representative GO terms enriched in URGs of KO-pre-neoplastic fibroblasts compared to WT fibroblasts. Dot size denotes the gene count and color denotes the adjusted p value.

(**H**) Dot plot showing the expression of signature genes of fibroblasts at different stages. Both color and size indicate the effect size.

(**I**) Bar plot showing the number of sequenced cells per epithelial-cell subcluster prepared from different tissues.

(**J**) Violin plots showing representative marker genes of epithelial-cell subclusters.

(**K**) CNV plot of epithelial cells employing inferCNV. Bottom labels indicate different chromosome regions and color bars indicate different cell clusters (left). Box plots showing CNV scores of different epithelial-cell subclusters from WT/KO group (right).

(**L**) Dot plot showing representative GO terms enriched in marker genes of the breast carcinoma based on functional enrichment analysis. Dot size denotes the gene count and color denotes the adjusted p value.

(**M**) Hierarchical clustering tree showing representative GO terms enriched in URGs of KO-pre-neoplastic epithelial cells compared to WT epithelial cells. Dot size denotes the gene count and color denotes the adjusted p value. WT, wild-type; KO, knockout

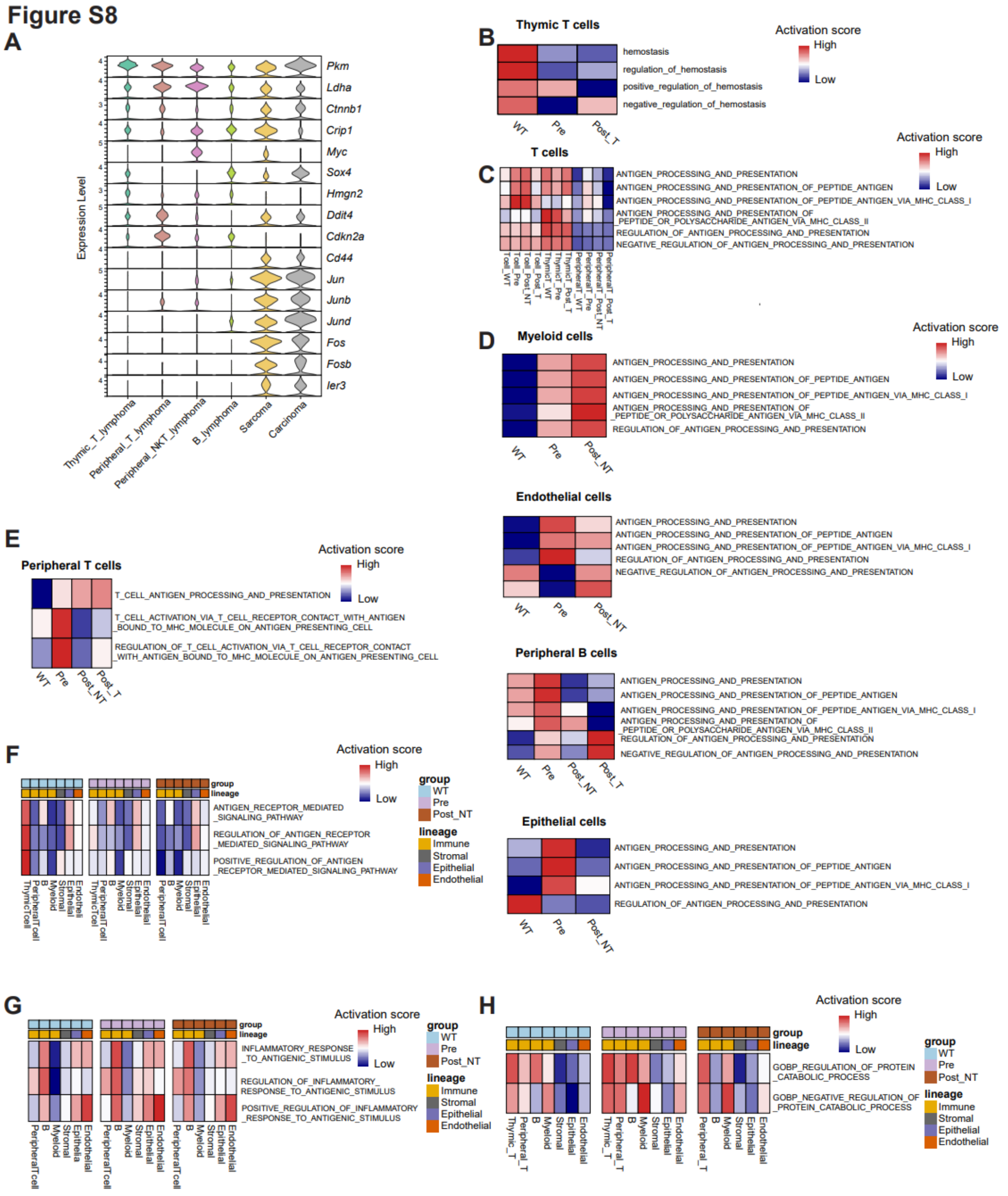


**Fig. S8. Assessment of essential pathway activity intensities in response to p53 loss.**

(**A**) Violin plots showing representative marker genes of different tumor types.

(**B**) Heatmap showing hemostasis-related pathway activity scores of thymic T cells across different states by GSVA.

(**C**) Heatmap showing the activity scores associated with antigen processing and presentation pathways of T cells across different states by GSVA.

(**D**) Heatmap showing the activity scores associated with antigen processing and

presentation pathways of myeloid, peripheral B, endothelial, and epithelial cells across different states by GSVA.

(**E**) Heatmap showing the activity scores associated with T cell antigen processing, presentation and activation pathways of peripheral T cells across different states by GSVA.

(**F**) Heatmap showing pathway activities associated with inflammatory response scored per lineage across different states by GSVA.

(**G**) Heatmap showing antigen receptor-mediated signaling pathway activities scored per lineage across different states by GSVA.

(**H**) Heatmap showing protein catabolic pathway activities scored per lineage across different states by GSVA.

## Figure S9

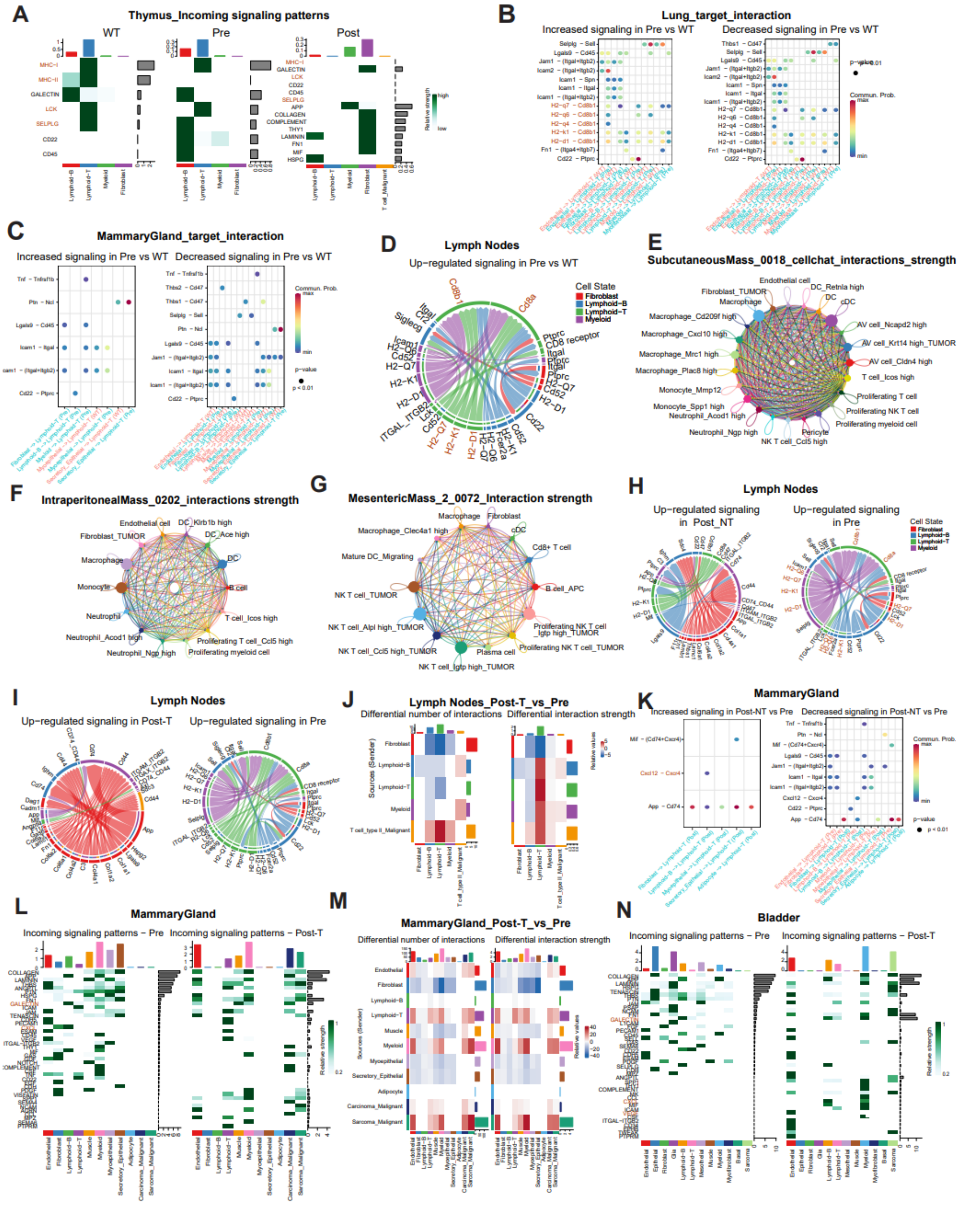


**Fig. S9. Cell-cell communication analysis across different states.**

(**A**) Heatmaps showing relative incoming signal strength across different thymic cell populations for WT (left), KO-pre-neoplastic (center) and KO-neoplastic (right) samples.

(**B**) Bubble charts showing increased (left) and decreased (right) signaling for pulmonary T cell targets at KO-pre-neoplastic states compared to WT states.

(**C**) Bubble charts showing increased (left) and decreased (right) signaling for breast T cell targets at KO-pre-neoplastic states compared to WT states.

(**D**) Chord diagram showing increased signaling for immune cell targets in lymph nodes at KO-pre-neoplastic states compared to WT states.

(**E**) Circle plot showing the strength of interactions among different cell populations of the subcutaneous mass. Edge width is proportional to the strength of interactions.

(**F**) Circle plot showing the strength of interactions among different cell populations of the intraperitoneal mass. Edge width is proportional to the strength of interactions.

(**G**) Circle plot showing the strength of interactions among different cell populations of the mesenteric mass. Edge width is proportional to the strength of interactions.

(**H**) Chord diagram showing increased (left) and decreased (right) signaling for immune cell targets in lymph nodes at KO-neoplastic-NonTumor states compared to KO-pre-neoplastic states.

(**I**) Chord diagram showing increased (left) and decreased (right) signaling for immune cell targets in lymph nodes at KO-neoplastic-Tumor states compared to KO-pre-neoplastic states.

(**J**) Heatmap showing differential number of interactions (left) and differential interaction strength (right) among different cell populations of lymph nodes at KO-neoplastic-Tumor states compared to KO-pre-neoplastic states.

(**K**) Bubble charts showing increased (left) and decreased (right) signaling for breast T cell targets at KO-neoplastic-NonTumor states compared to KO-pre-neoplastic states.

(**L**) Heatmaps showing relative incoming signal strength across different cell populations for KO-pre-neoplastic (left) and KO-neoplastic-Tumor (right) cells of mammary gland.

(**M**) Heatmap showing differential number of interactions (left) and differential interaction strength (right) among different cell populations of mammary gland at KO-neoplastic-Tumor states compared to KO-pre-neoplastic states.

(**N**) Heatmaps showing relative incoming signal strength across different cell

populations for KO-pre-neoplastic (left) and KO-neoplastic-Tumor (right) cells of bladder. WT, wild-type; KO, knockout

## Figure S10

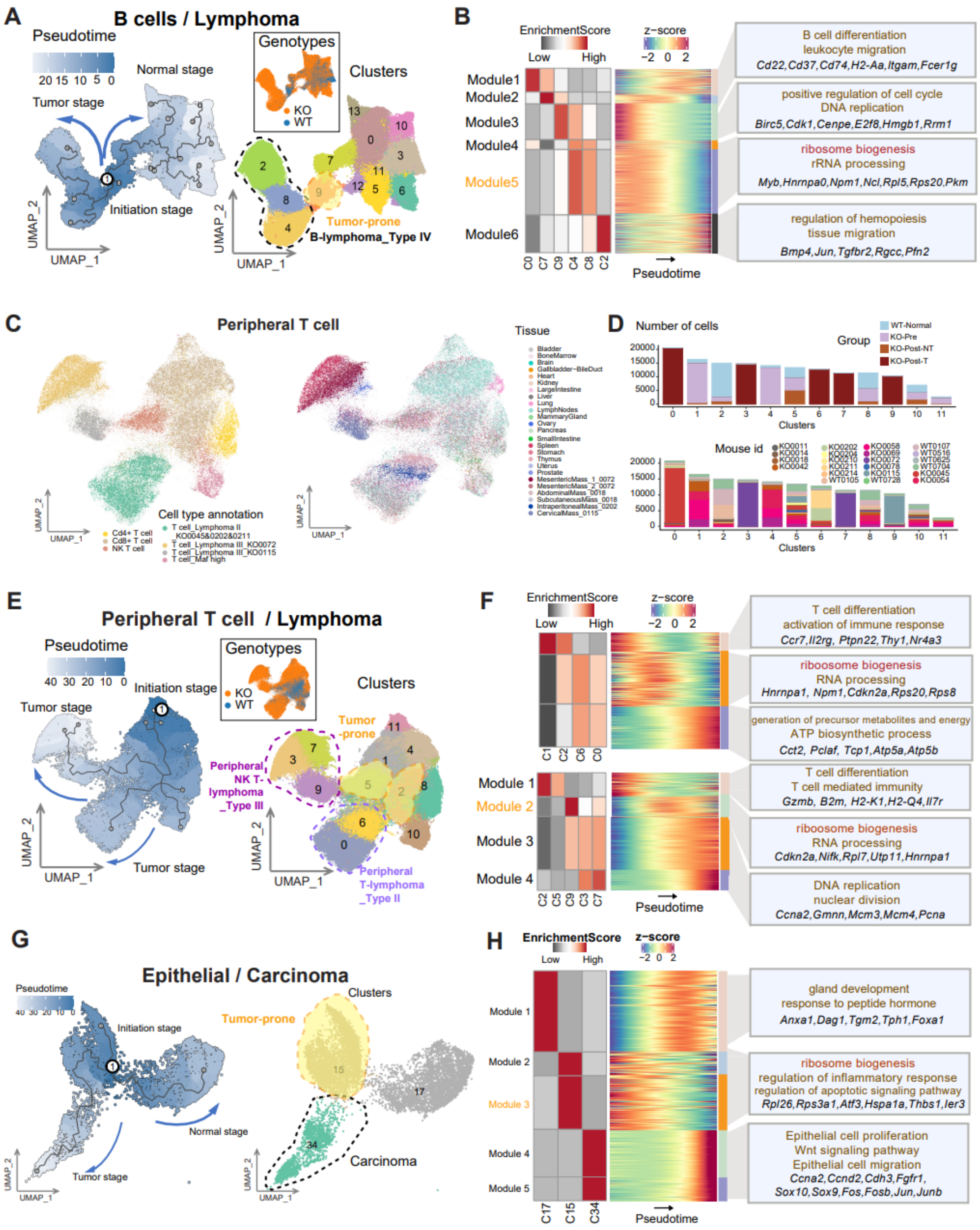


**Fig. S10. Pseudotemporal reconstruction of the tumorigenesis trajectory.**

(**A**) Pseudotemporal reconstruction of the B-cell lymphomagenesis trajectory. UMAP visualization of the trajectory, colored by pseudotime (bottom-left). UMAP visualization of B cells, colored by cell subclusters (bottom-right) and genotypes (top), respectively.

(**B**) Heatmap showing the expression scores of identified pseudotemporal gene modules

for different B-cell subclusters (left) and branch-dependent gene expression patterns in pseudotime of B-cell lymphomagenesis trajectory (middle). Marker genes and enriched GO terms of branch-dependent genes are shown on the right panel.

(**C**) UMAP visualization of the peripheral T-cell lymphomagenesis trajectories, colored by pseudotime (left). UMAP visualization of peripheral T cells, colored by cell subclusters (right).

(**D**) Bar plot showing the number of sequenced cells per peripheral T-cell subtype prepared from different tissues (top) or from WT/KO-pre-neoplastic/KO-neoplastic samples (bottom).

(**E**) Pseudotemporal reconstruction of the peripheral T-cell lymphomagenesis trajectory. UMAP visualization of the trajectory, colored by pseudotime (bottom-left). UMAP visualization of peripheral T cells, colored by cell subclusters (bottom-right) and genotypes (top), respectively.

(**F**) Heatmap showing the expression scores of identified pseudotemporal gene modules for different peripheral T-cell subclusters (left) and branch-dependent gene expression patterns in pseudotime of peripheral T-cell lymphomagenesis trajectory (middle). Marker genes and enriched GO terms of branch-dependent genes are shown on the right panel.

(**G**) UMAP visualization of the breast carcinomagenesis trajectories, colored by pseudotime (left). UMAP visualization of breast epithelial cells, colored by subclusters (right).

(**H**) Heatmap showing the correlation between identified pseudotemporal gene modules and fibroblast subclusters. (left). Heatmap of branch-dependent gene expression patterns in pseudotime of breast carcinomagenesis trajectory (center). Marker genes and enriched GO terms of branch-dependent genes are shown on the right panel. WT, wild-type; KO, knockout

## Figure S11

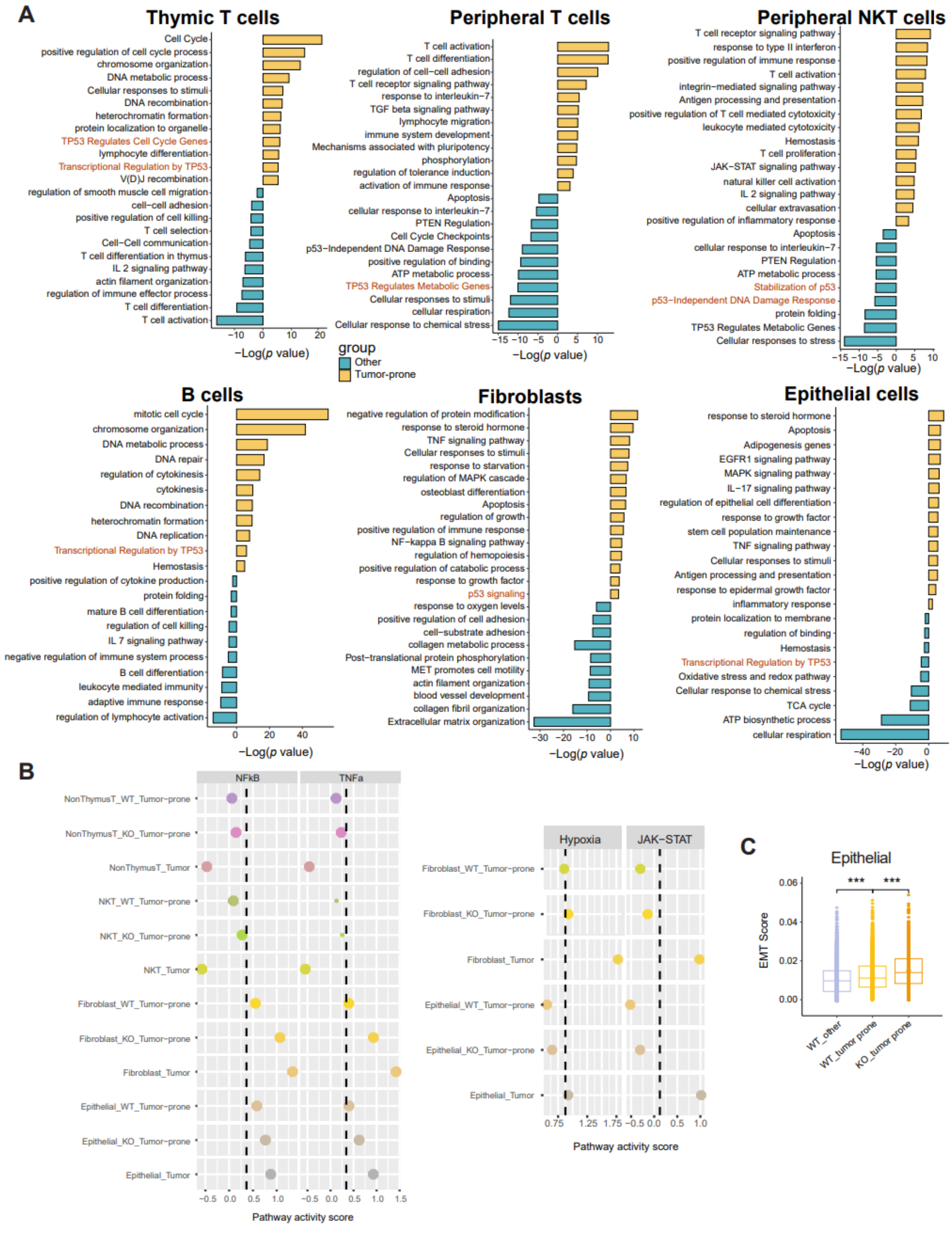


**Fig. S11. The characteristics of tumor-prone cells across lineages.**

(**A**) Barplot showing the representative signal pathways enriched in differentially expressed genes between tumor-prone cells and other cells from the same lineage.

(**B**) Average pathway activity (columns) across different cell clusters (rows). The black

dashed line represents the cluster-specific mean pathway scores.

(**C**) Boxplot showing the Epithelial-Mesenchymal Transition (EMT) module scores of different epithelial cell groups.

**Figure S12**

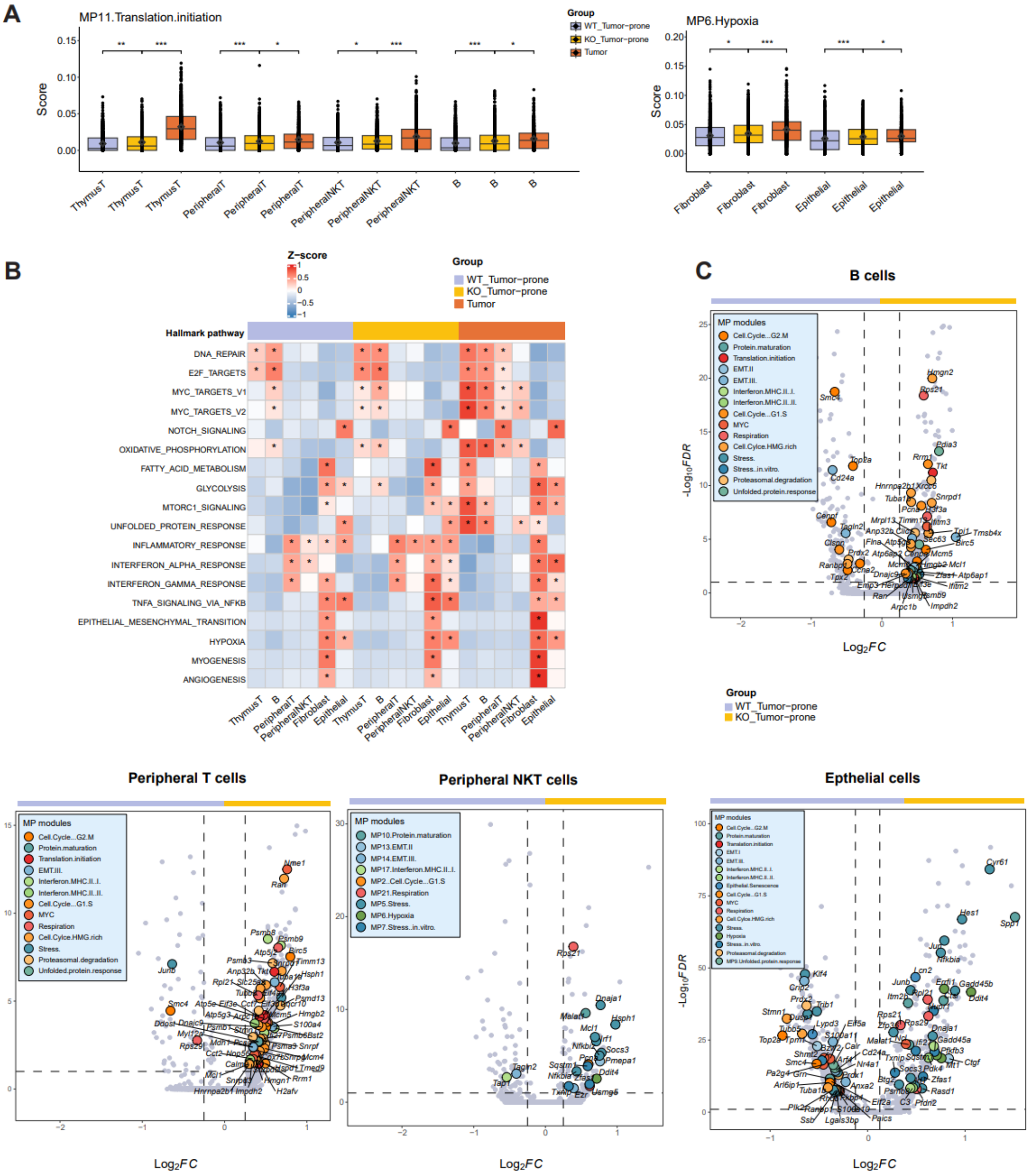

**Fig. S12. The genetic alternations of tumor-prone cells upon p53 loss across lineages.**

(**A**) Boxplot showing the MP gene module scores of different cell groups across different cell lineages.

(**B**) Heatmap showing the Molecular Signatures Database (MSigDB) hallmark gene module scores of different tumor-prone cell groups across different lineages (Z-scores above 0.15 marked with an asterisk).

(**C**) Volcano plot showing the differential expressed genes between WT and KO-pre-neoplastic tumor-prone cells (left). Colors represent the MP gene modules enriched with genes. WT, wild-type; KO, knockout

## Figure S13

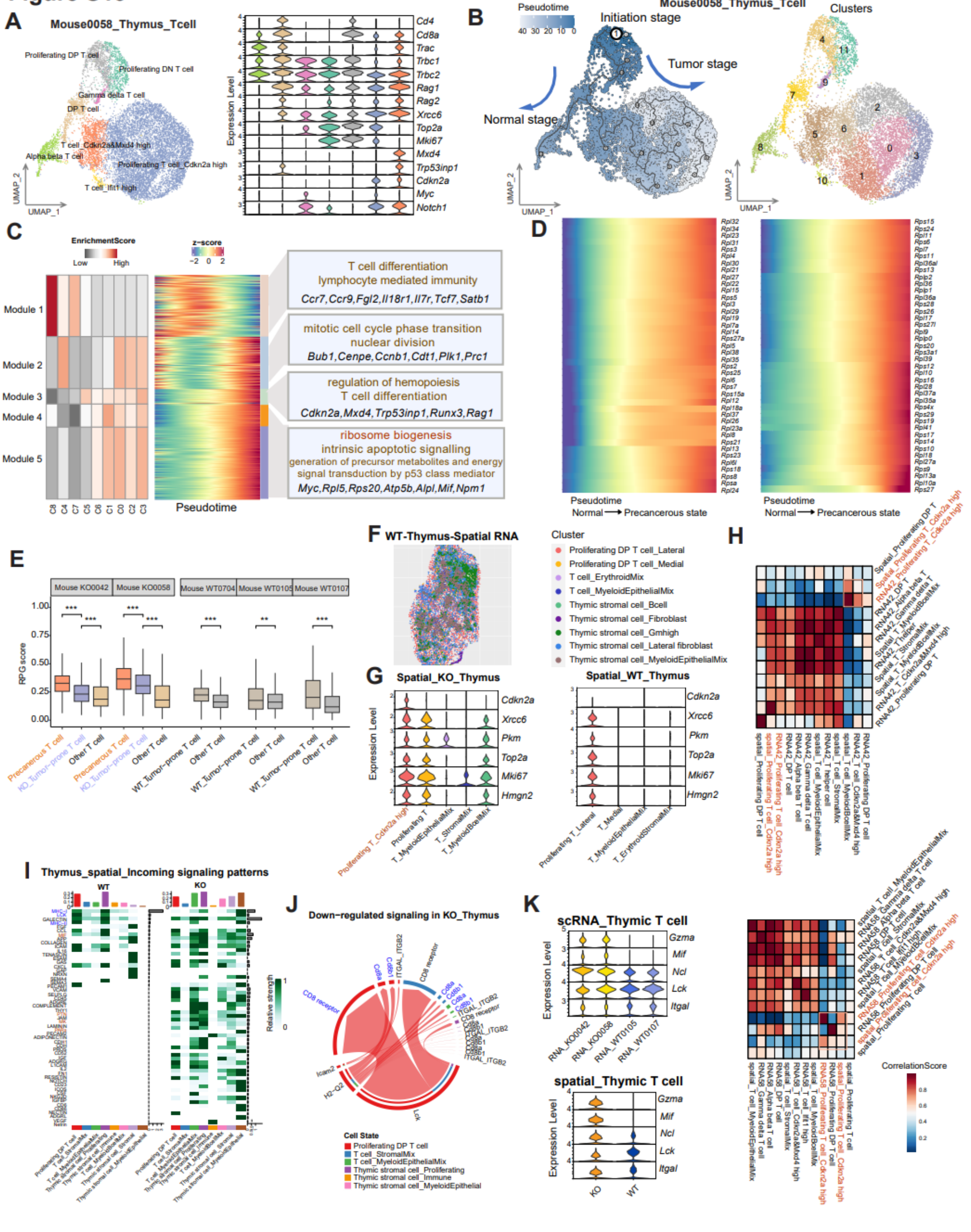


**Fig. S13. Spatial comparison of thymus transcriptomes between KO-pre-neoplastic and WT samples.**

(**A**) UMAP visualization of T cells from mouse KO0058, colored by cell types (left). Violin plots showing representative marker genes of T cell subclusters from mouse KO0058 (right).

(**B**) Pseudotemporal reconstruction of the thymic early T-cell lymphomagenesis

trajectory of mouse KO0058. UMAP visualization of trajectories, colored by pseudotime (left). UMAP visualization of thymic T cells, colored by cell subclusters (right).

(**C**) Heatmap showing the correlation between identified pseudotemporal gene modules and thymic T cell subclusters of mouse KO0058. (left). Heatmap of branch-dependent gene expression patterns in pseudotime of early thymic T-cell lymphomagenesis trajectory (middle). Marker genes and enriched GO terms of branch-dependent genes are shown on the right panel.

(**D**) Heatmaps showing the gradually increased expression levels of ribosomal protein genes (RPGs) during the early tumorigenesis of thymic T cells from mouse KO0042.

(**E**) Boxplot showing the RPG expression scores of thymic T-cell subclusters from mouse KO0042, KO0058, WT0704, WT0105 or WT0107. The RPGs that exhibited progressive up-regulation during the early tumorigenesis of thymic T cells in Fig. 4G were selected for analysis.

(**F**) Spatial projection of WT mouse thymus section colored by cell types.

(**G**) Violin plots showing representative marker genes of KO (left) and WT (right) thymic T cell subtypes from spatial RNA data.

(**H**) Heatmap displaying the correspondence between spatial RNA data from mouse KO0606 and scRNA-seq data from mouse KO0042 (top) or KO0058 (bottom) of KO thymic T cells.

(**I**) Heatmaps showing relative incoming signal strength across different thymic cell populations for WT (left) and KO (right) cells.

(**J**) Chord diagram showing decreased signaling for thymic immune cell targets at KO states compared to WT states.

(**K**) Violin plots showing representative marker genes of thymic T cells of different mice from scRNA-seq data (top) and spatial RNA data (bottom). WT, wild-type; KO, knockout

Figure S14

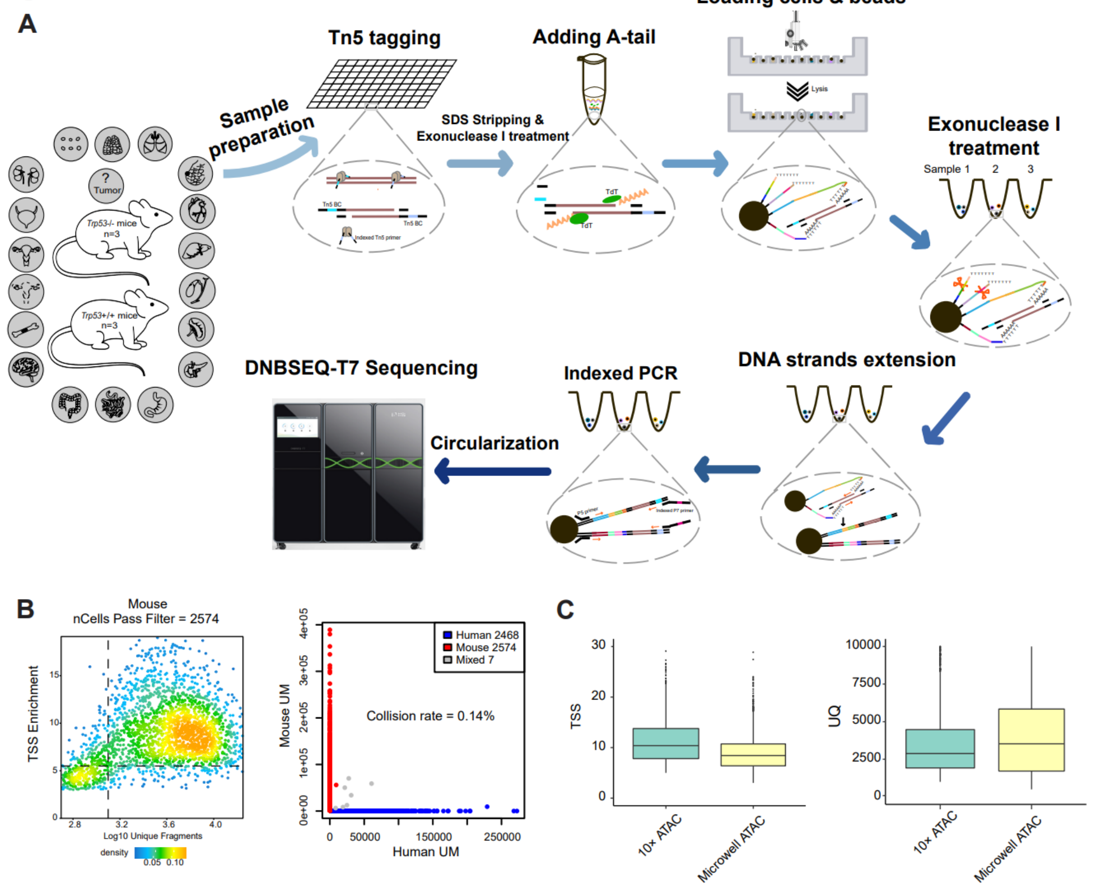


**Fig. S14. Schematic of updated Microwell ATAC-seq workflow and library quality of the platform.**

(**A**) After preparation of single-nucleus suspensions of fresh tissues from *Trp53*$^{-/-}$ and *Trp53*$^{+/+}$ mice, Tn5 transposase pre-tagging was performed in bulk and then poly A tails were added to the DNA fragments. The nuclei and barcoded beads were loaded into microwells and DNA fragments were captured. Next, through Exonuclease I treatment, DNA strands extension, DNA amplification and circularization, DNA libraries available for DNBSEQ-T7 sequencing were generated. For a more detailed description of key steps, see the supplementary material.

(**B**) ATAC-seq data quality control filters in mouse cells (3t3). Shown are the number of unique ATAC-seq nuclear fragments in each single cell (each dot) compared with TSS enrichment of all fragments in that cell (left). Dashed lines represent the filters for high-quality single-cell data (1,000 unique nuclear fragments and TSS score greater than or equal to 5). Density is given in arbitrary units (left). Human (293t) /mouse (3t3)

cell mixing experiment showing proportion of single-cell libraries with both mouse and human ATAC-seq fragments (right).

(**C**) Box plots showing the comparison of unique ATAC-seq nuclear fragments and transcription start site (TSS) score between 10×scATAC-seq and updated Microwell ATAC-seq.

## Figure S15

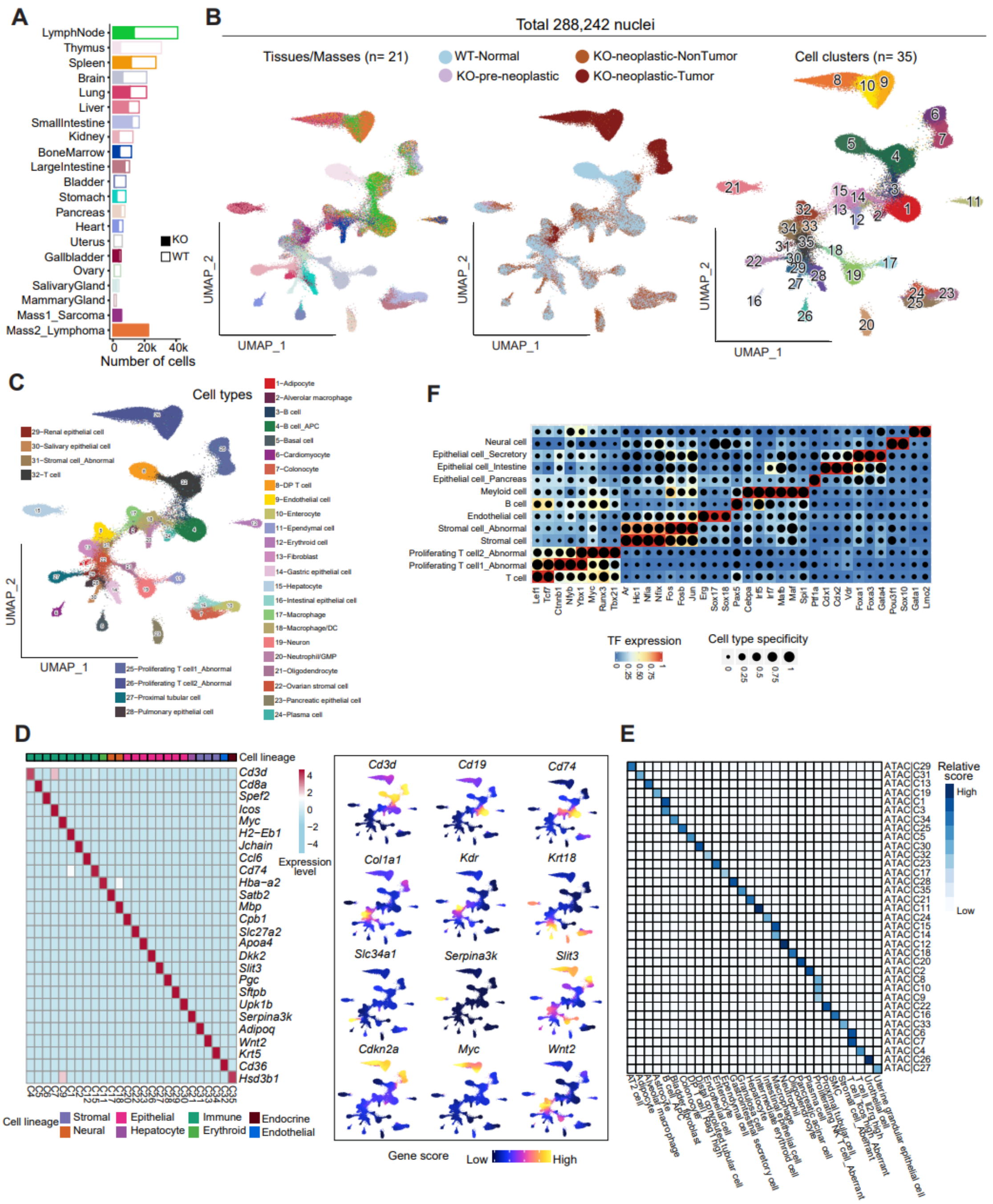

**Fig. S15. Chromatin accessibility profile of global cells.**

(**A**) Bar plot showing the number of sequenced nuclei per tissue/mass prepared from WT and KO samples.

(**B**) UMAP visualization of 288,242 nuclei from KO and WT samples, colored by tissues/masses (left), genotypes (center), and cell clusters (right), respectively.

(**C**) UMAP visualization of 288,242 nuclei from KO and WT samples, colored by cell

types.

(**D**) Heatmap showing the activity scores of cell-type-specific marker genes (left), and relative activity scores of representative markers overlaid on UMAP plots (right).

(**E**) Heatmap showing the correspondence between scRNA-seq atlas (row) and scATAC-seq atlas (column) from the label transfer analysis.

(**F**) SCENIC+ analysis of scATAC-seq and scRNA-seq for representative cell lineages and tumor cells. Heatmap-dot plot showing TF expression of enhancer-regulon (eRegulon) on the color scale and cell type specificity (RSS) of eRegulon on the size scale. WT, wild-type; KO, knockout

Figure S16

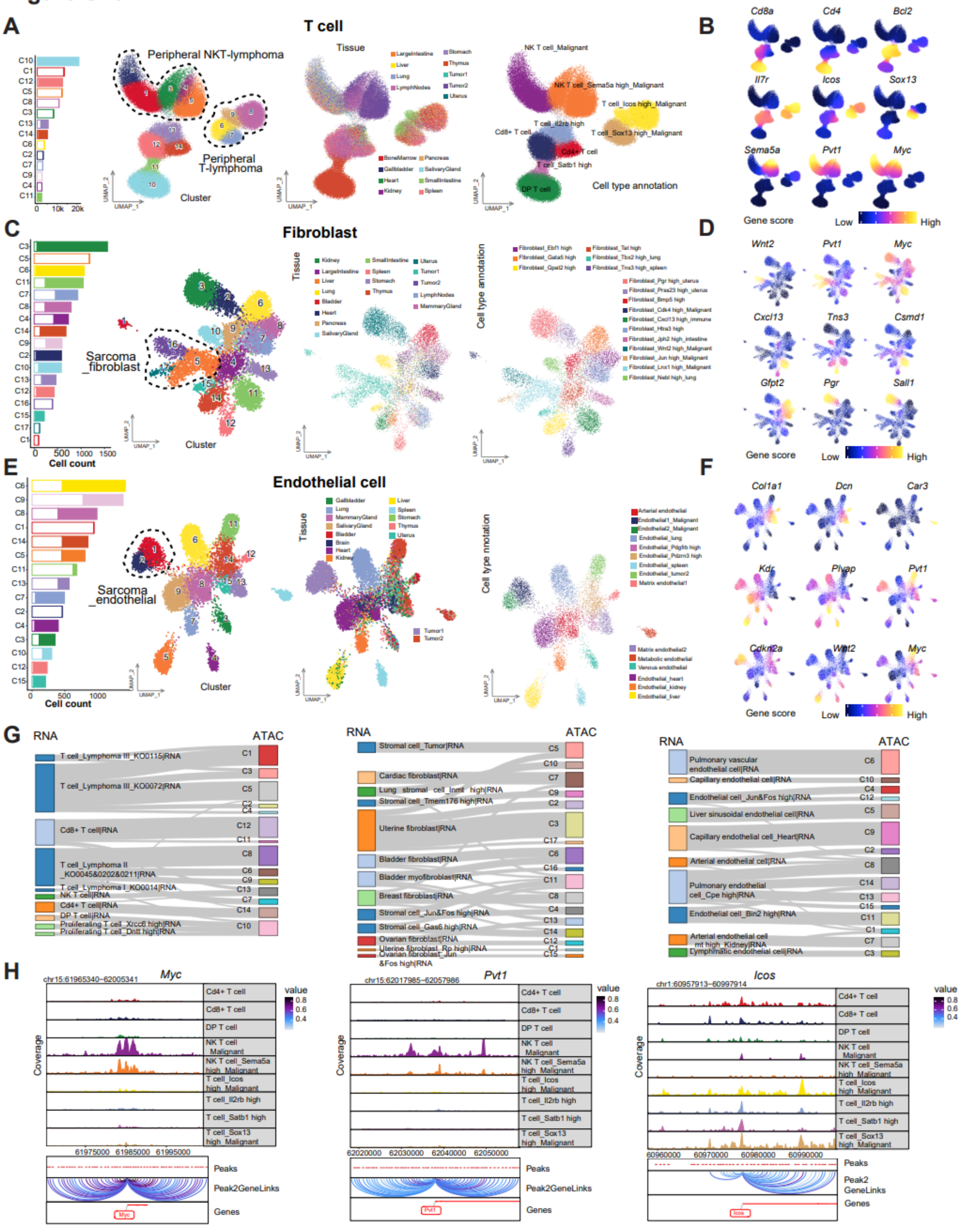

**Fig. S20. Chromatin accessibility profile of T cells, fibroblasts, endothelial cells.**

(**A**) UMAP embedding of all nuclei from the T cell lineage, colored by cell subclusters, tissues and cell-type annotations. The bar plot indicates the nuclei count of KO/WT mouse sample in each cell cluster (left).

(**B**) UMAP visualization of the marker genes for each T-cell subtype.

(**C**) UMAP embedding of all nuclei from the fibroblast lineage, colored by cell

subclusters, tissues and cell-type annotations. The bar plot indicates the nuclei count of KO/WT mouse sample in each cell cluster (left).

(**D**) UMAP visualization of the marker genes for each fibroblast subtype.

(**E**) UMAP embedding of all nuclei from the endothelial cell lineage, colored by cell subclusters, tissues and cell-type annotations. The bar plot indicates the nuclei count of KO/WT mouse sample in each cell cluster (left).

(**F**) UMAP visualization of the marker genes for each endothelial-cell subtype.

(**G**) Sankey plot showing the correspondence between scATAC-seq and scRNA-seq of T (left), fibroblast (center) and endothelial (right) cell-type assignments based on the label transfer strategy.

(**H**) Genome browser tracks of *Myc*, *Pvt1* and *Icos* in different T-cell subtypes. Peaks called on scATAC-seq data and peaks-to-gene links are shown below the tracks. WT, wild-type; KO, knockout.

## Figure S17

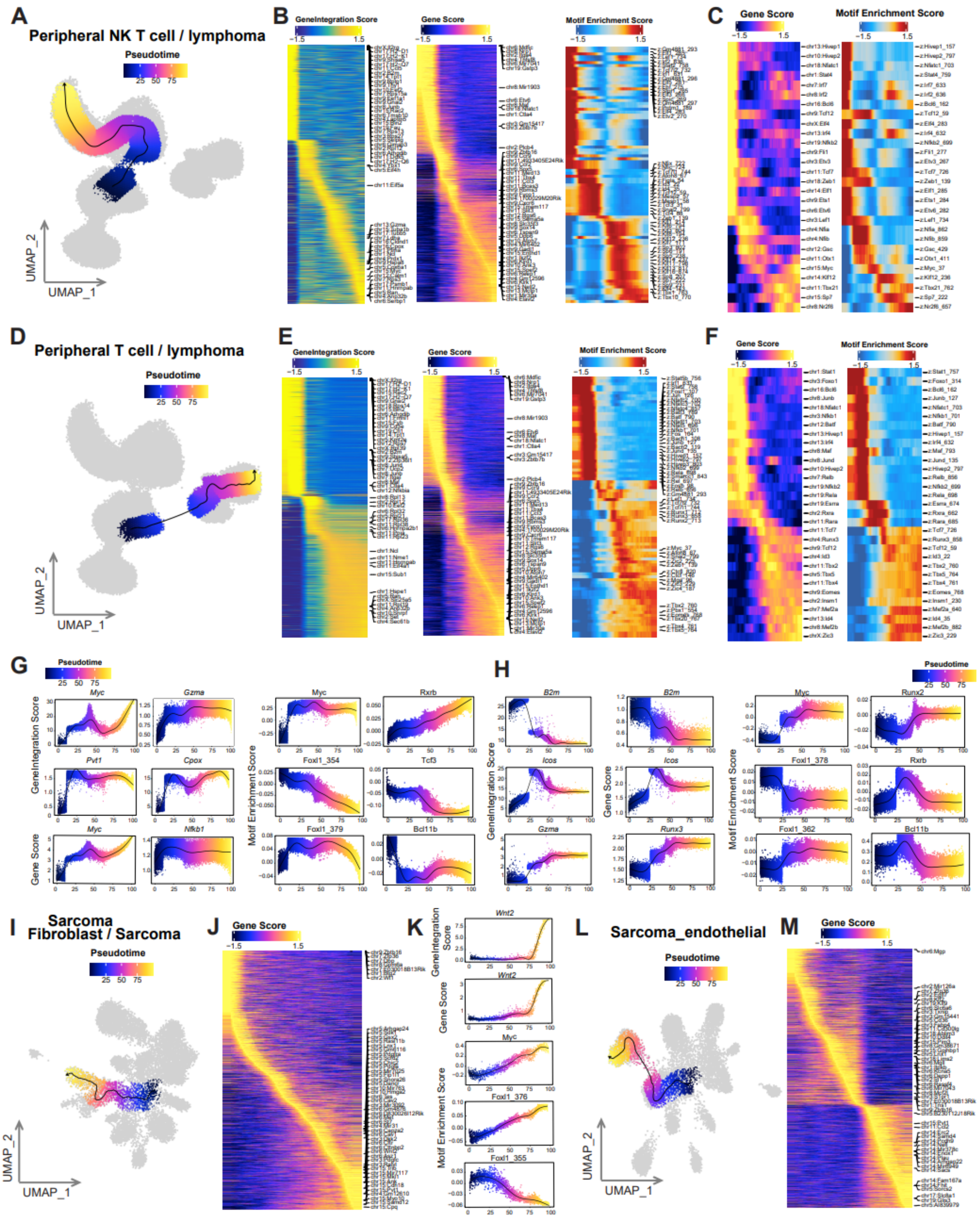


**Fig. S17. Pseudotemporal dynamics of T cells and fibroblasts.**

(**A**) UMAP visualization of the pseudotemporal trajectory of peripheral NKT-cell lymphomagenesis.

(**B**) Heatmap of gene integration scores (left), gene scores (middle) and motif enrichment scores (right) of altered genes or TFs along the constructed peripheral NKT-cell trajectory in pseudotime, respectively.

(**C**) Heatmap of the intersection of gene scores (left) and gene integration scores (right) of altered genes along the constructed peripheral NKT-cell trajectory in pseudotime, respectively.

(**D**) UMAP visualization of the pseudotemporal trajectory of peripheral T-cell lymphomagenesis.

(**E**) Heatmap of gene integration scores (left), gene scores (middle) and motif enrichment scores (right) of altered genes or TFs along the constructed peripheral T-cell trajectory in pseudotime, respectively.

(**F**) Heatmap of the intersection of gene scores (left) and gene integration scores (right) of altered genes along the constructed peripheral T-cell trajectory in pseudotime, respectively.

(**G**) Pseudotemporal trajectories of marker genes integration scores, genes scores and TF motif enrichment scores during the peripheral NKT-cell lymphomagenesis.

(**H**) Pseudotemporal trajectories of marker genes integration scores, genes scores and TF motif enrichment scores during the peripheral T-cell lymphomagenesis.

(**I**) UMAP visualization of the pseudotemporal trajectory of fibroblast sarcomagenesis.

(**J**) Heatmap of gene scores of altered genes along the constructed fibroblast trajectory in pseudotime, respectively.

(**K**) Pseudotemporal trajectories of marker genes integration scores, genes scores and TF motif enrichment scores during the fibroblast sarcomagenesis.

(**L**) UMAP visualization of the pseudotemporal trajectory of endothelial-cell sarcomagenesis.

(**M**) Heatmap of gene scores of altered genes along the constructed endothelial-cell trajectory in pseudotime, respectively.

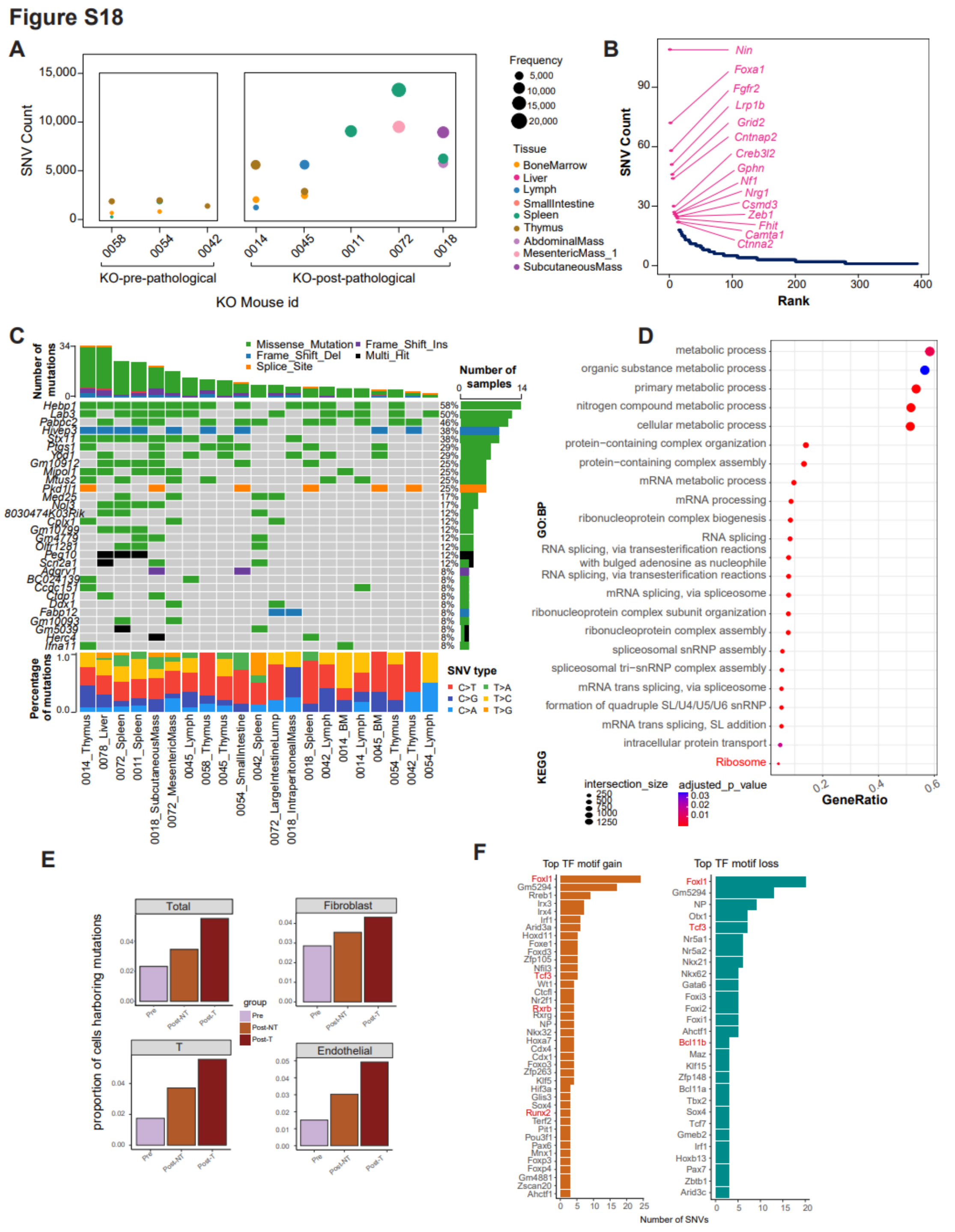

**Fig. S18. The mutation analysis by incorporating WGS and scATAC-seq data.**

**(A)** Detectable single nucleotide variant (SNV) counts in tissues from *Trp53* KO mice during tumorigenesis.

**(B)** Dot plot showing the counts of the most frequently detected SNVs in *Trp53* KO mouse for cancer-associated genes reported in COSMIC Cancer Gene Census (CGC).

**(C)** Oncoplot displaying the landscape of annotated high-confidence SNVs, which

potentially change protein stability and structure. Genes are ordered by their mutation frequency; the bar plot (right) showing the detectable high-confidence SNV counts for each gene; samples are ordered according to SNV counts; the bar plot (top) and stacked bar plot (bottom) showing the distribution of high-confidence SNV counts and mutation spectra for each sample, respectively.

**(D)** Dot plot showing enriched GO terms and KEGG pathways for detectable SNVs in promoter regulatory regions based on functional enrichment analysis.

(**E**) Bar plot showing the comparison of proportions of individual cells with mutation detection between different stages of global cells and diverse lineages (T, B, fibroblast and epithelial cells).

(**F**) Bar plots showing the top 25 gain TF motifs (left) and top 25 loss TF motifs (right).

## Figure S19

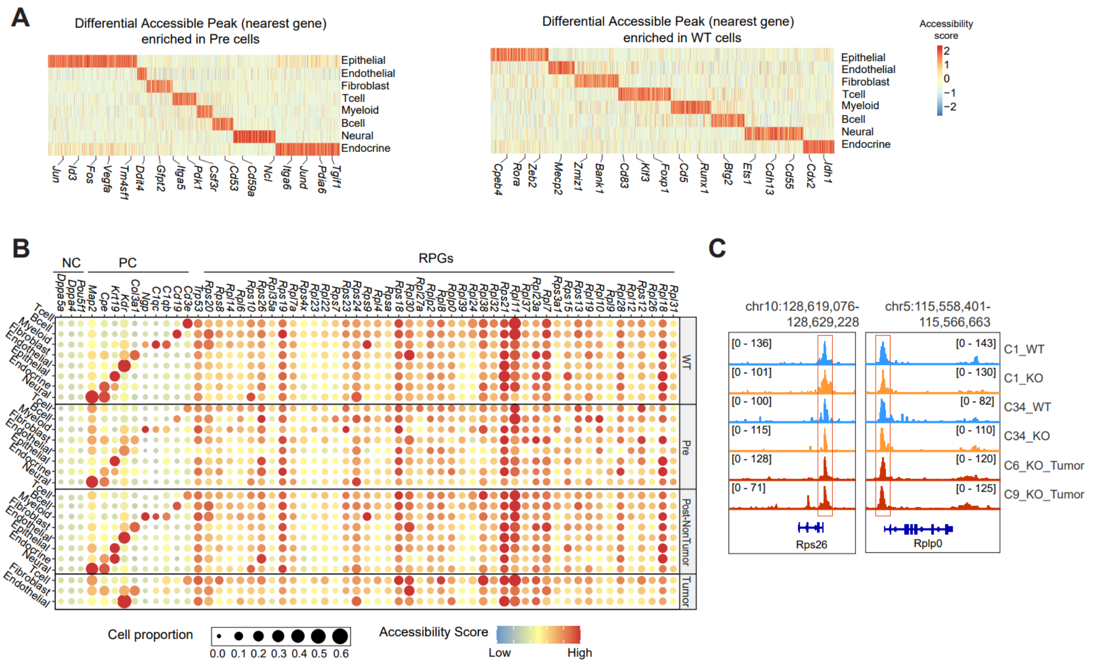


**Fig. S19. Accessible peak analysis across lineages.**

(**A**) Heatmap showing the differential accessible peaks of diverse cell lineages enriched in KO-pre-neoplastic samples compared to WT samples (left) or enriched in WT samples compared to KO-pre-neoplastic samples (right), labeled by nearest genes.

(**B**) Heatmap showing the gene accessibility scores of different cell lineages across different stages.

(**C**) Normalized sequencing tracks of scATAC-seq peaks for different cell subclusters.

NC, negative control; PC, positive control; WT, wild-type; KO, knockout.

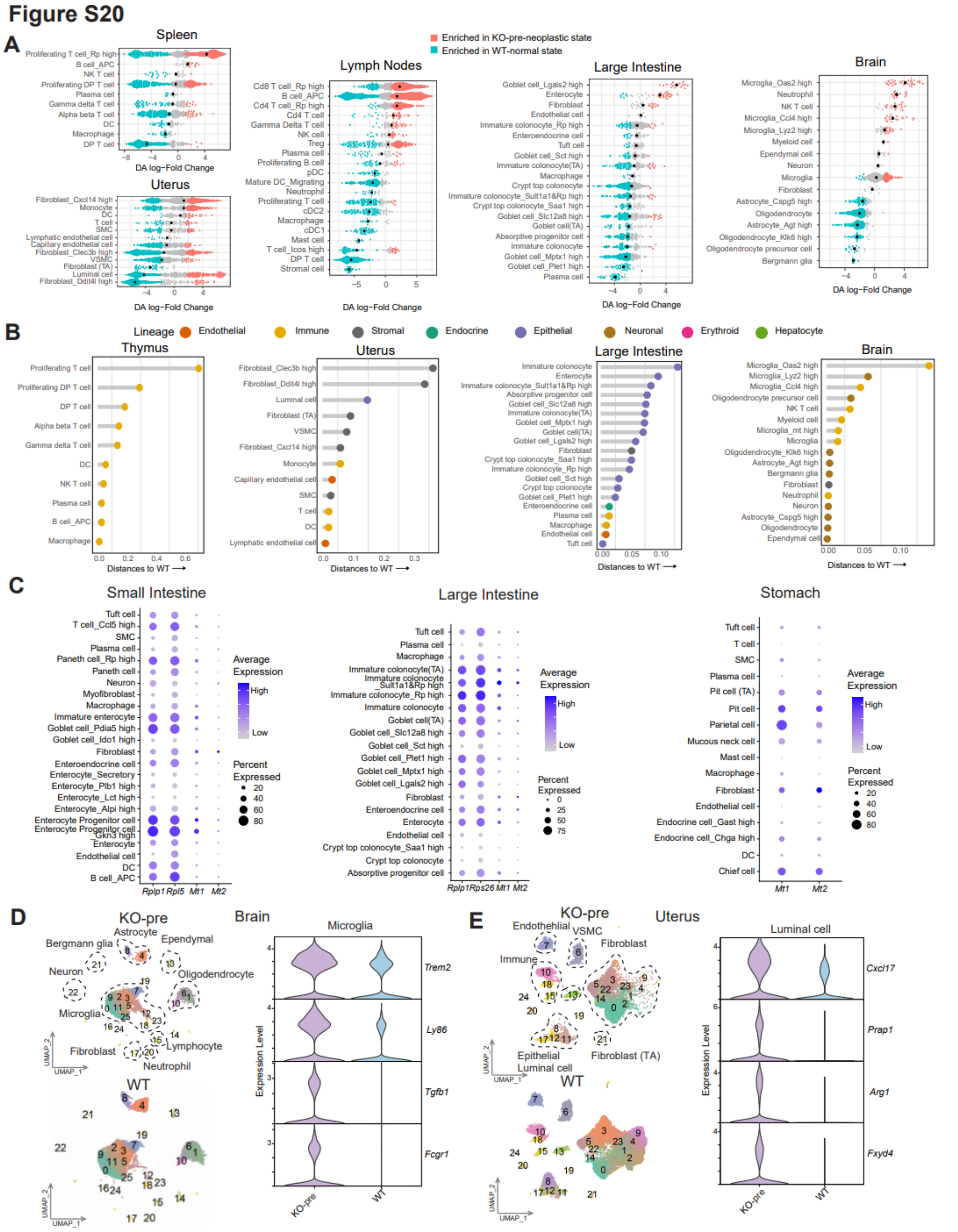

**Fig. S20. Comparative analysis between WT and KO-pre-neoplastic cell types.**

(**A**) Bee-swarm plot of log-fold change (x-axis) in cell abundance between two samples in Milo neighborhoods. Results from spleen, uterus, lymph nodes, large intestine, and brain are shown. Neighborhoods overlapping the same cell population are grouped together (y-axis) and colored if displaying significant differential abundance (DA)

(spatial FDR < 5%). Non-differential abundance neighborhoods are colored gray, otherwise red or blue.

(**B**) Lollipop plots showing the cosine distance of cell types between WT and KO-pre-neoplastic samples. Results from thymus, uterus, brain, and large intestine are shown.

(**C**) Dot plot showing the expression of signature genes of each cluster of three tissues (small intestine, large intestine, and stomach). Both color and size indicate the effect size.

(**D**) UMAP visualization of KO-pre-neoplastic and WT cell clusters split by KO/WT groups from brain (left). Violin plots showing representative marker genes of KO microglia cells versus WT microglia cells (right).

(**E**) UMAP visualization of KO-pre-neoplastic and WT cell clusters split by KO/WT groups from uterus (left). Violin plots showing representative marker genes of KO luminal cells versus WT luminal cells (right).

WT, wild-type; KO, knockout.

Figure S21

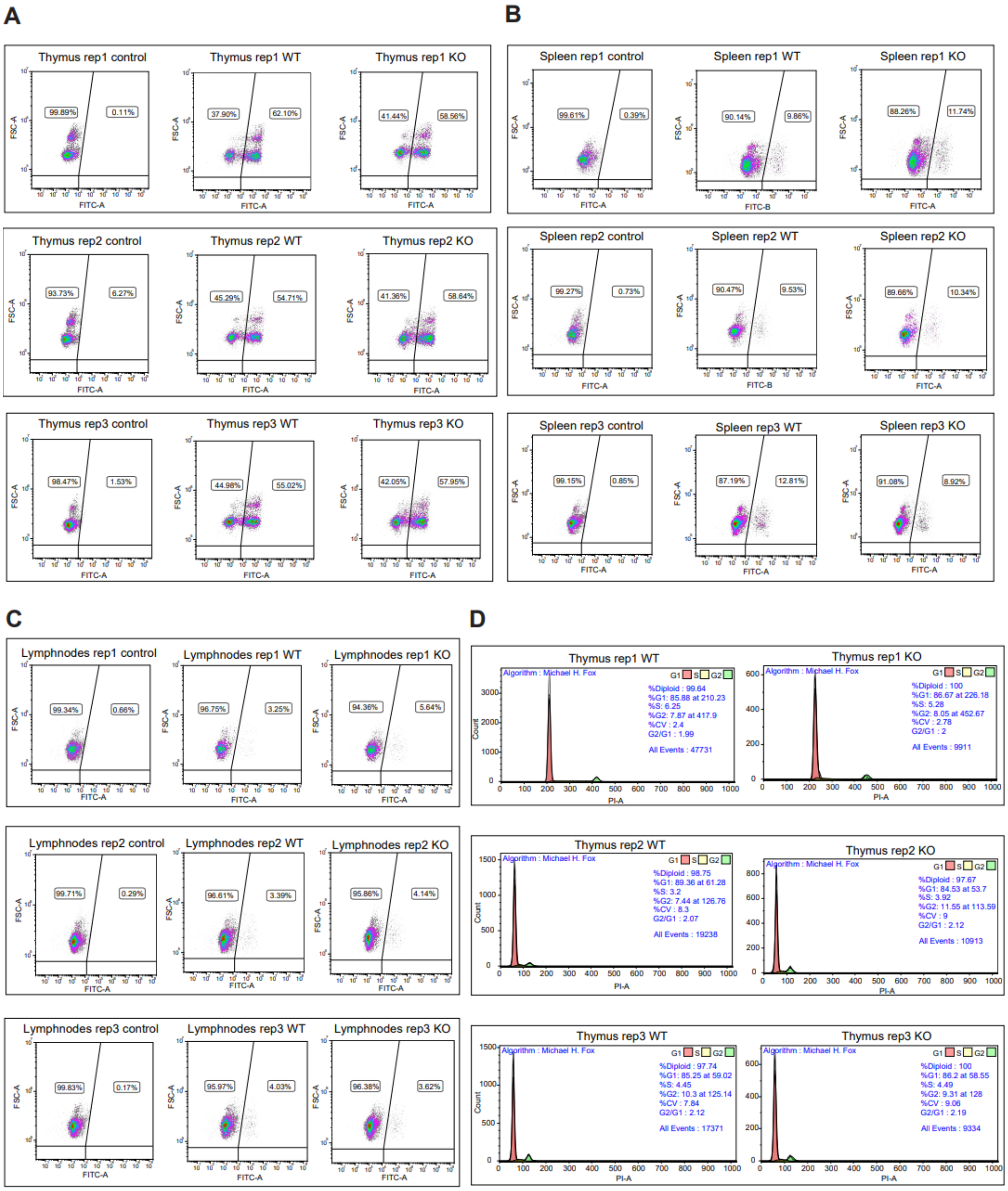


**Fig. S21. Cell proliferation analysis of KO-pre-neoplastic and WT immune cells by in vivo EdU incorporation.**

(**A**) Scatter plots showing the distribution of filtered EdU-positive and EdU-negative thymic immune cells. The results of three independent repeated experiments are displayed. Each framed group includes a negative control (left), a WT sample (middle), and a KO-pre-neoplastic sample (right).

(**B**) Scatter plots showing the distribution of filtered EdU-positive and EdU-negative immune cells of spleen. The results of three independent repeated experiments are displayed. Each framed group includes a negative control (left), a WT sample (middle), and a KO-pre-neoplastic sample (right).

(**C**) Scatter plots showing the distribution of filtered EdU-positive and EdU-negative immune cells of lymph nodes. The results of three independent repeated experiments are displayed. Each framed group includes a negative control (left), a WT sample (middle), and a KO-pre-neoplastic sample (right).

(**D**) Histograms showing the distribution of PI staining intensity for thymic immune cells. The proportions of cells at G1, G2M, and S phase are fitted using the Michael H. Fox algorithm. The results of three independent repeated experiments are displayed. Each framed group includes a WT sample (left) and a KO-pre-neoplastic sample (right). WT, wild-type; KO, knockout.

**Figure S22**

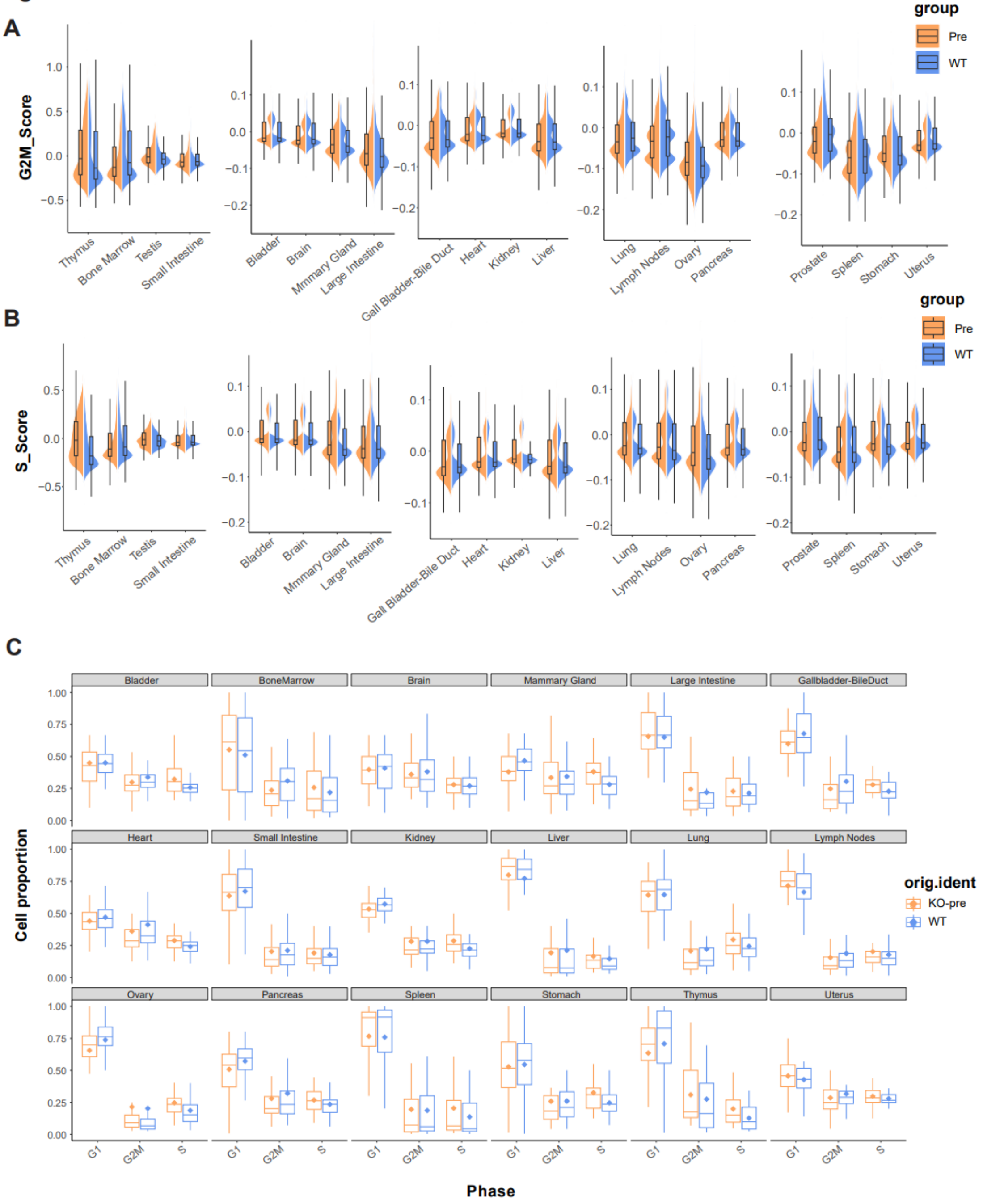


**Fig. S22. Cell proliferation and cell cycle analysis of KO-pre-neoplastic and WT tissues via scRNA-seq data.**

(**A**) Split violin plots showing the G2M scores of KO-pre-neoplastic and WT tissues.

(**B**) Split violin plots showing the S scores of KO-pre-neoplastic and WT tissues.

(**C**) Box plots showing the cell proportions at G1, G2M, and S phase of KO-pre-neoplastic and WT tissues. WT, wild-type; KO, knockout.

## Figure S23

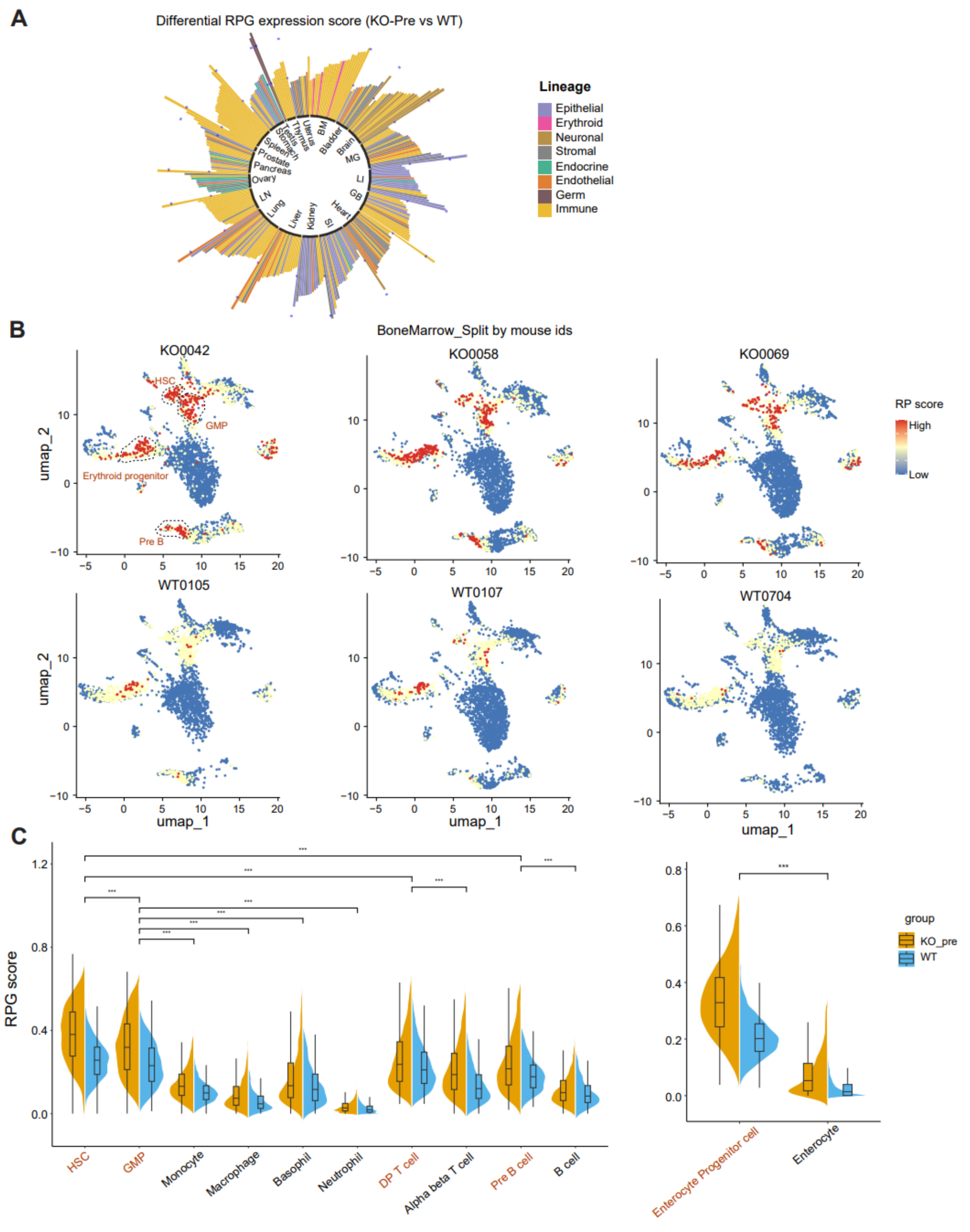


**Fig. S23. RPG expression of KO-pre-neoplastic and WT cells across diverse cell types.**

(**A**) Bar chart showing the AUCell scores of differential ribosomal protein gene (RPG) expression between KO-pre-neoplastic samples and WT samples for each cell type. Top RPGs (up-regulated top RPGs across more than 50 cell types in Fig.5C) at KO-pre-

neoplastic stage were chosen for calculation.

(**B**) UMAP visualization of the RPG scores of KO-pre-neoplastic and WT cell types split by mice from bone marrow.

(**C**) Violin plots showing the RPG scores of progenitor cells and corresponding differentiated cell types from diverse tissues.

WT, wild-type; KO, knockout.

## Figure S24

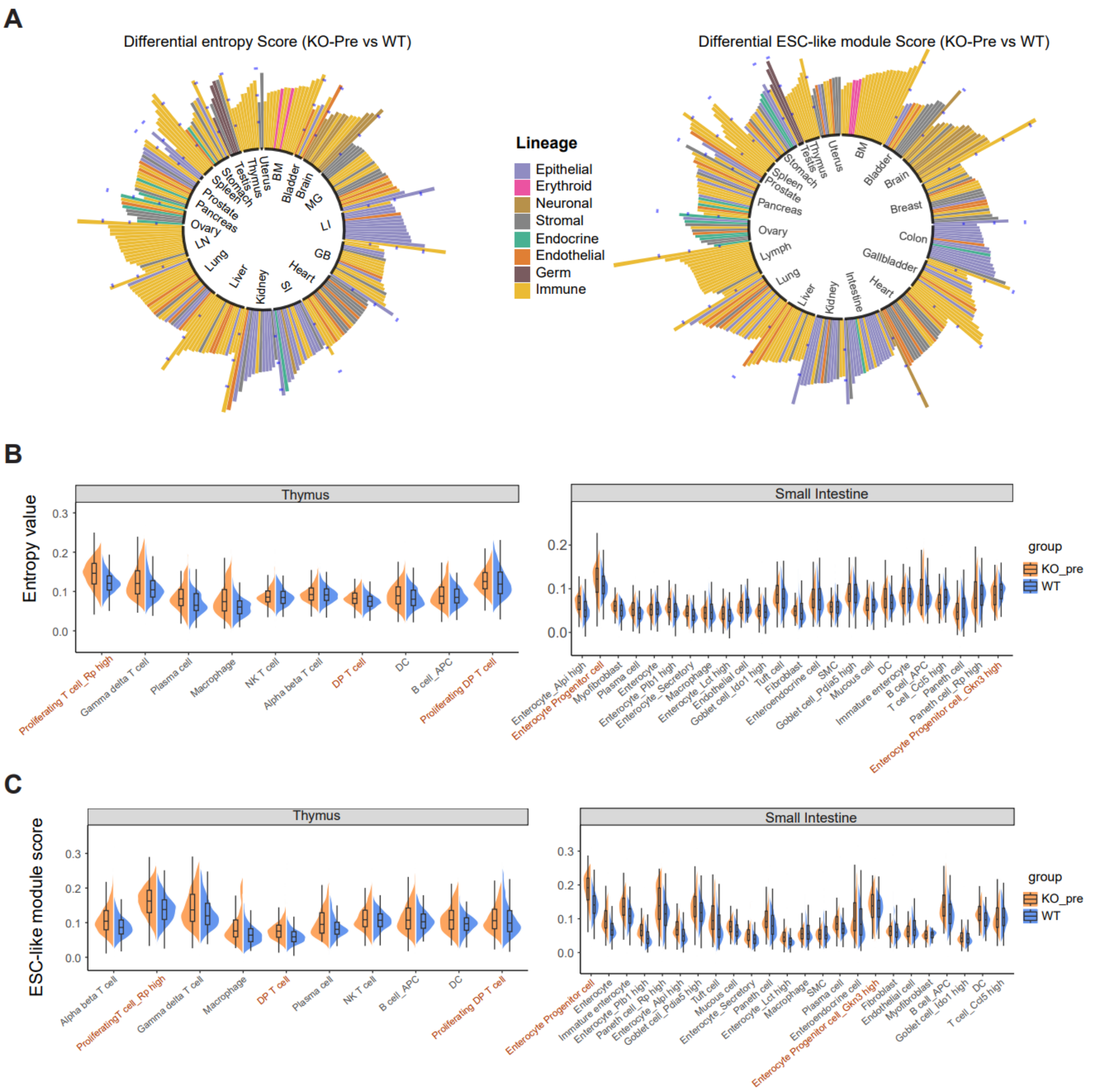


**Fig. S24. Entropy values and Myc-centered ESC-like module scores of KO-pre-neoplastic and WT cells across diverse cell types.**

(**A**) Bar chart showing the differential entropy values between KO-pre-neoplastic and WT samples for each cell type.

(**B**) Bar chart showing the differential Myc-centered ESC-like module scores between KO-pre-neoplastic and WT samples for each cell type.

(**C**) Split violin plots showing the entropy values of KO-pre-neoplastic and WT cell types from thymus and small intestine calculated using CCAT.

(**D**) Split violin plots showing the Myc-centered ESC-like module scores of KO-pre-neoplastic and WT cell types from thymus and small intestine calculated using AUCell. WT, wild-type; KO, knockout.

## Figure S25

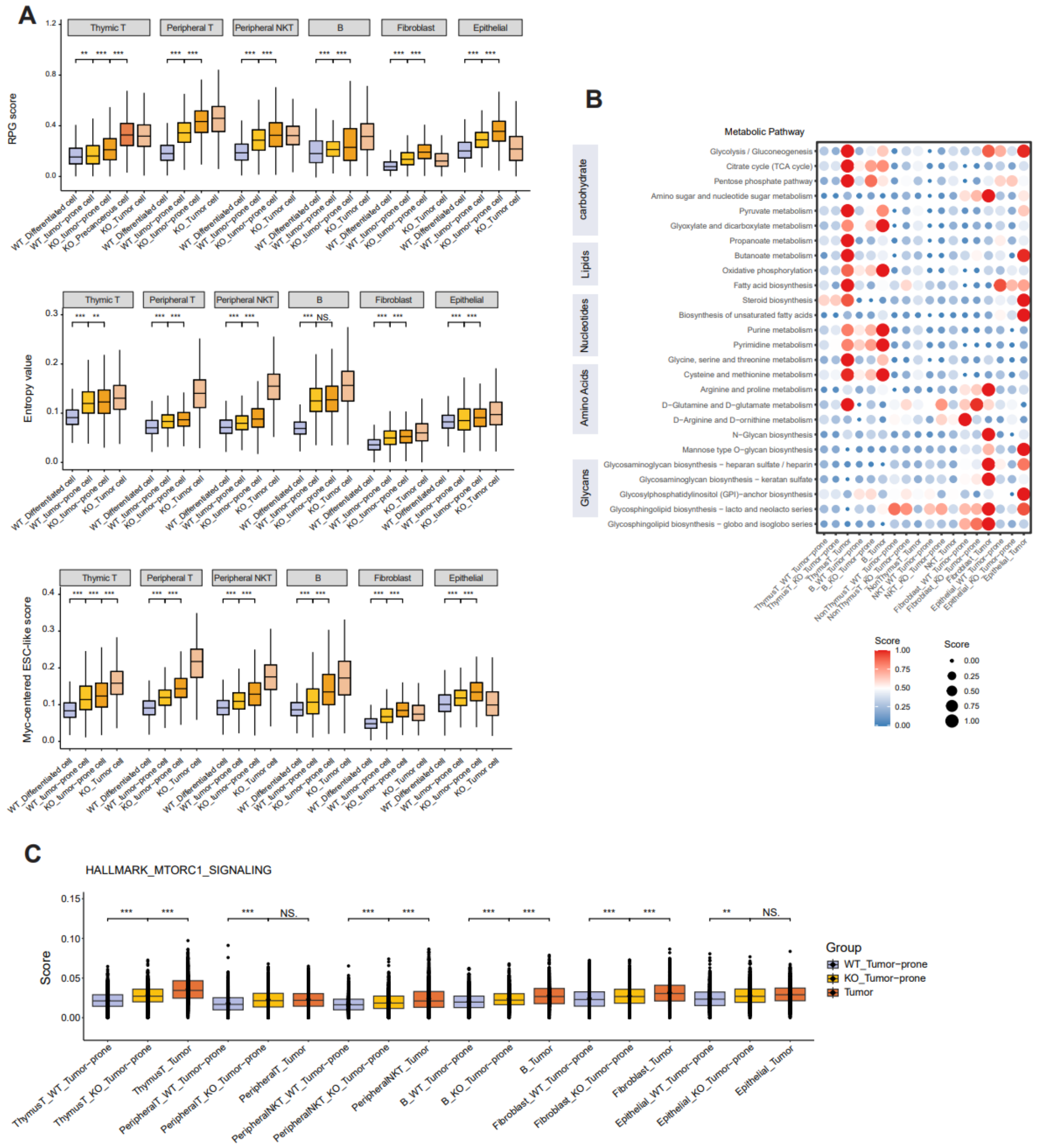

**Fig. S25. The RPG scores and metabolic reprogramming of tumor-prone cells across lineages.**

(**A**) Boxplot showing ribosomal protein gene (RPG) scores (top), entropy values (middle) and Myc-centered ESC-like module scores (bottom) of different cell groups across lineages.

(**B**) Dot plot showing the metabolic pathway activity scores of different cell groups across lineages.

(**C**) Boxplot showing the mTOR pathway scores of different tumor-prone cell groups across lineages.

## Figure S26

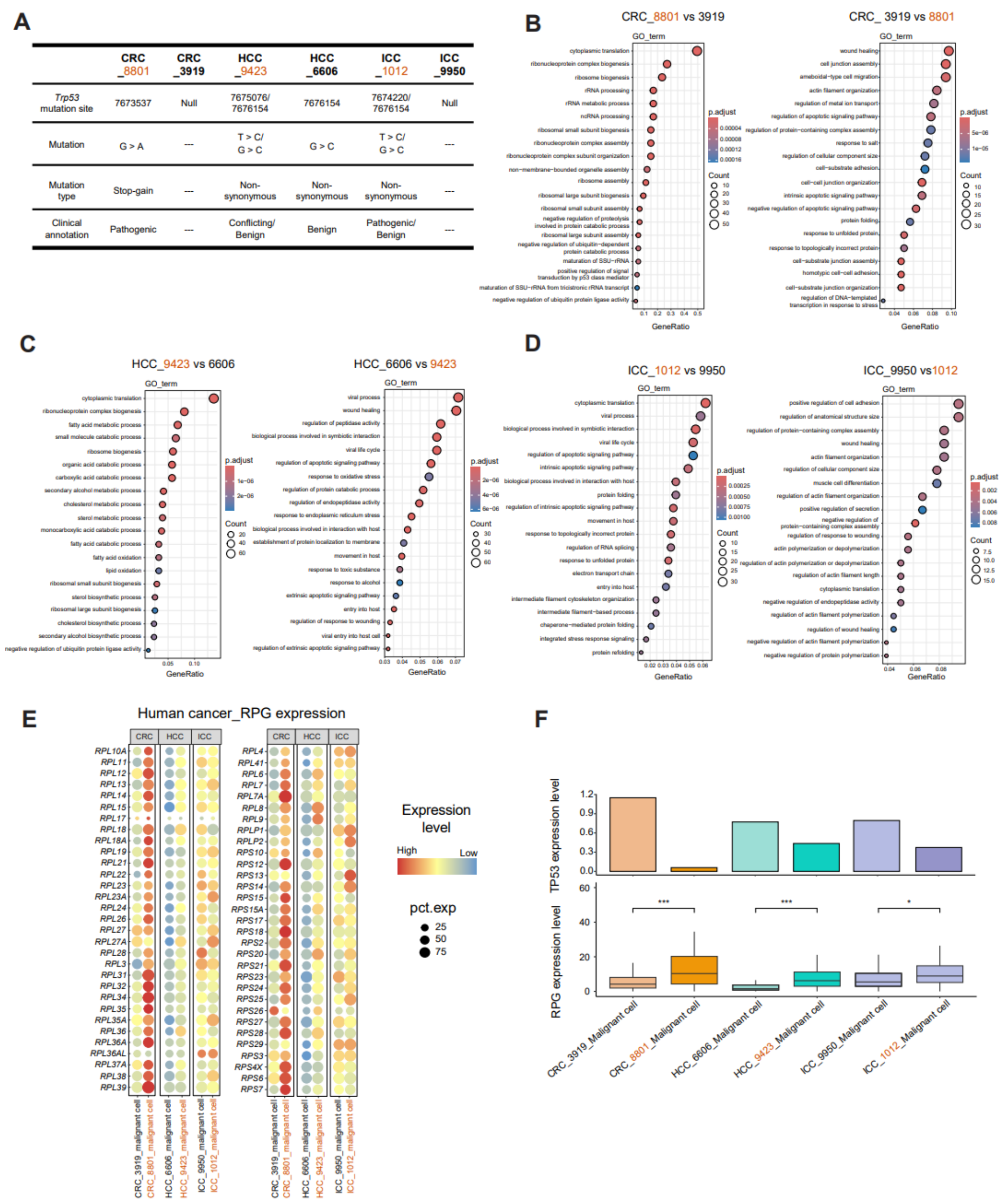


**Fig. S26. The specific RPGs were upregulated in human cancer tissues with *TP53* deleterious mutation.**

(**A**) The table displays the *TP53* mutations and their effects in different cancer samples. (**B**) Dot plot showing GO analysis results for up-regulated (left) and down-regulated (right) genes in cancer cells from CA of CRC8801, compared to cancer cells from CA of CRC3919. Dot size denotes the gene count and color denotes the adjusted p value. (**C**) Dot plot showing GO analysis results for up-regulated (left) and down-regulated

(right) genes in cancer cells from CA of HCC9423, compared to cancer cells from CA of HCC6606. Dot size denotes the gene count and color denotes the adjusted p value.

(**D**) Dot plot showing GO analysis results for up-regulated (left) and down-regulated (right) genes in cancer cells from CA of ICC1012, compared to cancer cells from CA of ICC9950. Dot size denotes the gene count and color denotes the adjusted p value.

(**E**) Heatmap showing individual RPG expression levels across tumor cells of different cancer types and genotypes.

(**F**) Boxplot showing *TP53* gene expression (top) and collective RPGs (Fig. S24E) expression (bottom) across tumor cells of different cancer types.

CA, cancer; ADJ, cancer-adjacent tissue; CRC, colorectal cancer; HCC, hepatocellular carcinoma; ICC, intrahepatic cholangiocarcinoma. Public tumor sequencing data (CRC_8801_CA, HCC_9423_CA and ICC_1012_CA) used in this figure were obtained from the Genome Sequence Archive for Human (GSA-Human) at the National Genomics Data Center, Chinese Academy of Sciences (accessions: HRA006591 and HRA004497; https://ngdc.cncb.ac.cn/gsa-human). Quality control and data preprocessing followed the original studies (Zhang et al., 2024, *National Science Review*, PMCID: PMC11771446).

## Figure S27

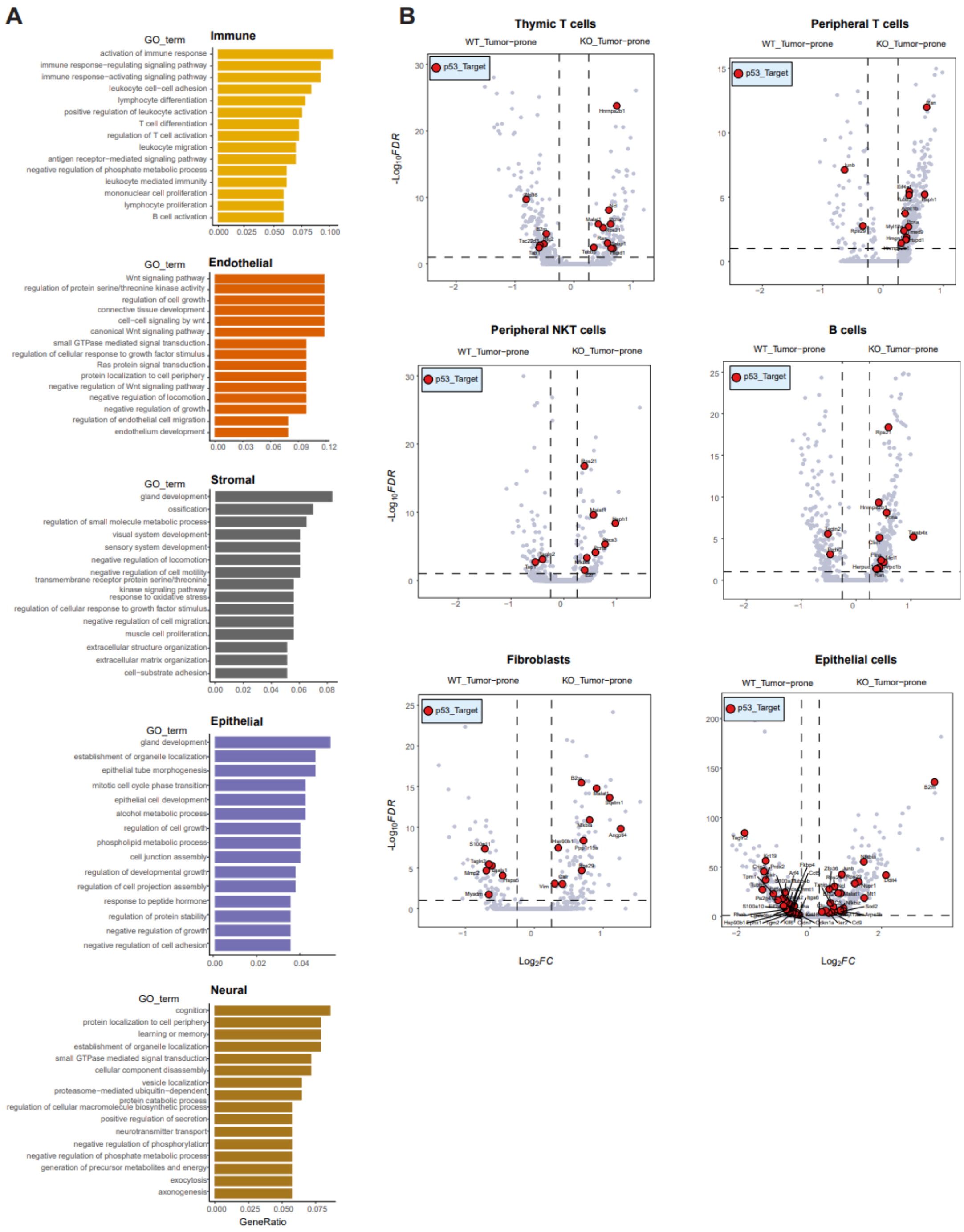


**Fig. S27. The functional analysis of p53 targets.**

(**A**) Bar chart showing representative GO terms enriched in lineage-specific p53 target genes of immune, stromal, epithelial, endothelial, and neural cells based on functional enrichment analysis.

(**B**) Volcano plot showing the differential expressed genes between WT and KO-pre-

neoplastic tumor-prone cells from different lineages. Red color represents the tumor-related p53 target gene subset. WT, wild-type; KO, knockout.

## Figure S28

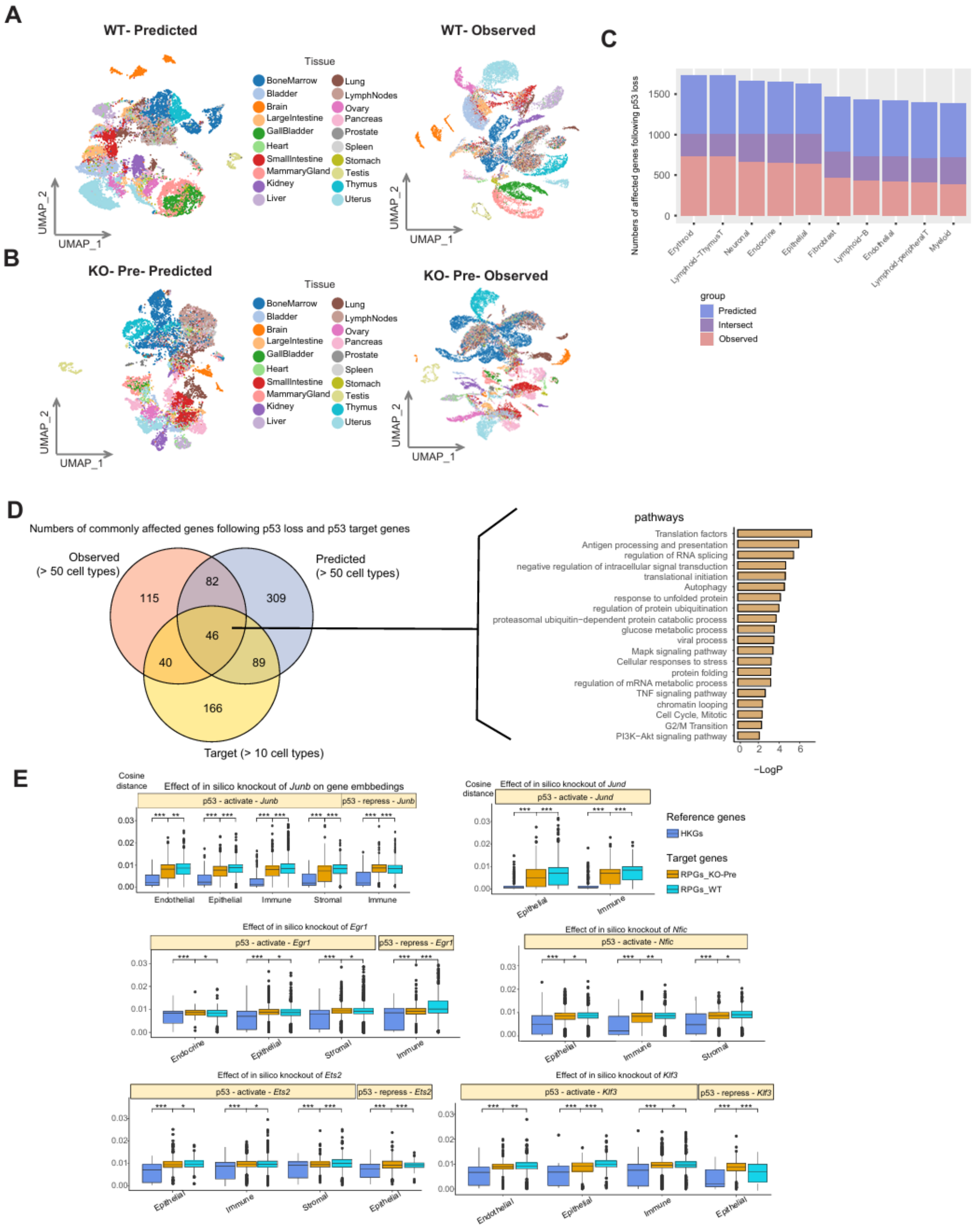


**Fig. S28. The Deep learning analysis of *Trp53* regulatory program.**

(**A**) UMAP plot showing WT cell embeddings of both model-predicted and experimental scRNA data (down sampled to 20,000 cells), colored by tissues.

(**B**) UMAP plot showing KO-pre-neoplastic cell embeddings of both model-predicted and experimental scRNA data (down-sampled to 20,000 cells), colored by tissues.

(**C**) Bar chart showing the number of the intersection of top affected genes upon p53

inactivation across different lineages from prediction and observation.

(**D**) Venn plot showing the number of the intersection of commonly affected genes upon p53 inactivation across cell types from prediction and observation and p53 target genes (left). Bar chart showing the representative signaling pathways enriched in the intersected genes.

(**E**) Boxplot showing the effect of in silico knockout of p53 target genes that can regulate top ribosomal protein genes (RPGs) in different cell lineages (Fig. 6E), measured by cosine distance. Alternations of the expression level of housekeeping genes in both WT and KO-pre-neoplastic cells, and top RPGs in WT/KO-pre-neoplastic cells following the perturbation were delineated, respectively.

WT, wild-type; KO, knockout.

Figure S29

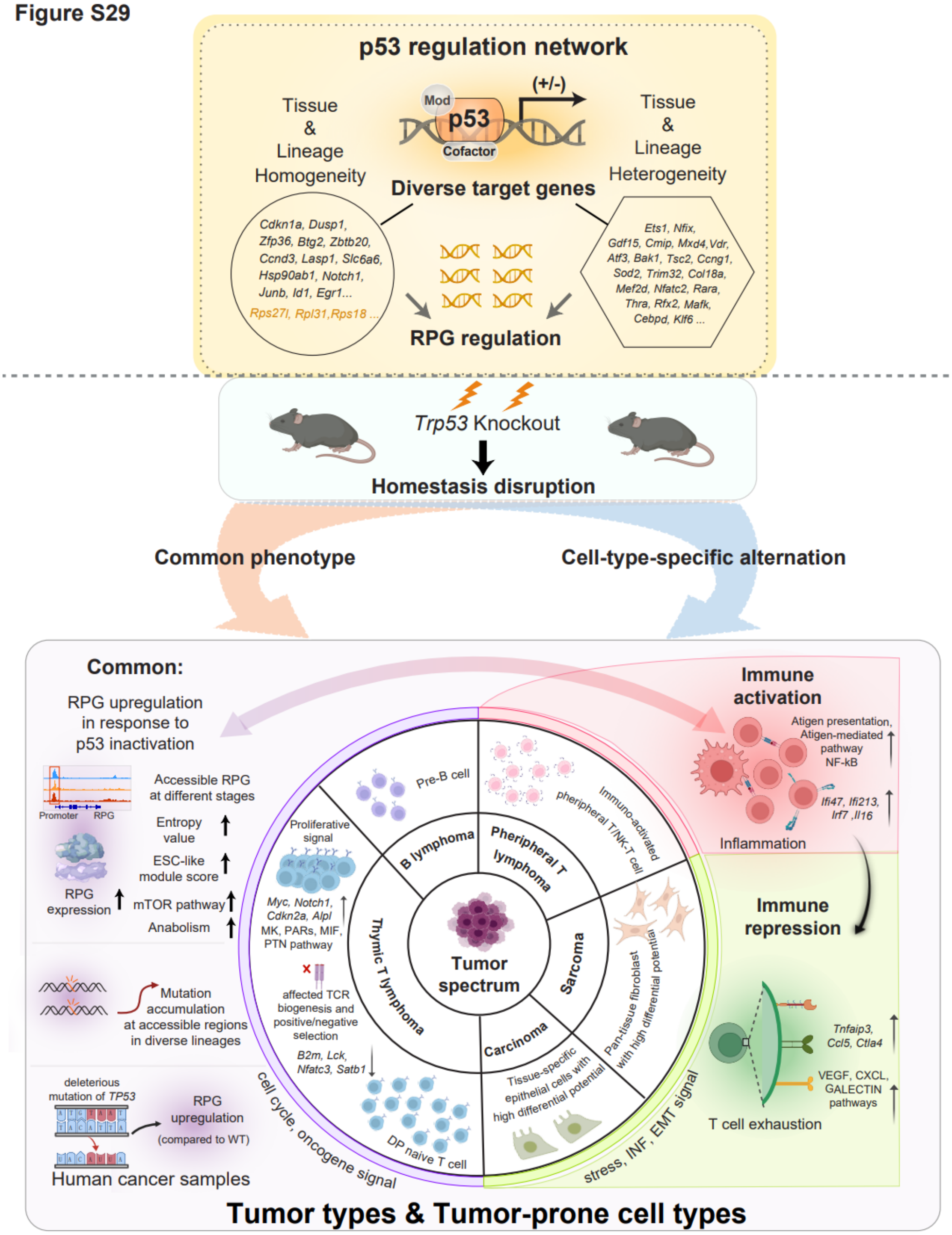


**Fig. S29. A schematic of key findings from our study.**
